# Shedding Light on the Interactions of Hydrocarbon Ester Substituents upon Formation of Dimeric Titanium(IV) Triscatecholates in DMSO Solution

**DOI:** 10.1002/chem.201904639

**Published:** 2020-01-22

**Authors:** A. Carel N. Kwamen, Marcel Schlottmann, David Van Craen, Elisabeth Isaak, Julia Baums, Li Shen, Ali Massomi, Christoph Räuber, Benjamin P. Joseph, Gerhard Raabe, Christian Göb, Iris M. Oppel, Rakesh Puttreddy, Jas S. Ward, Kari Rissanen, Roland Fröhlich, Markus Albrecht

**Affiliations:** ^1^ Institut für Organische Chemie RWTH Aachen University Landoltweg 1 52074 Aachen Germany; ^2^ Institut für Anorganische Chemie RWTH Aachen University Landoltweg 1 52074 Aachen Germany; ^3^ University of Jyväskylä Department of Chemistry P.O. Box 35 Jyväskylä 40014 Finland; ^4^ Organisch-Chemisches Institut Universität Münster Corrensstrasse 40 48149 Münster Germany

**Keywords:** coordination compounds, helicate, solvent effects, thermodynamics, weak interactions

## Abstract

The dissociation of hierarchically formed dimeric triple lithium bridged triscatecholate titanium(IV) helicates with hydrocarbyl esters as side groups is systematically investigated in DMSO. Primary alkyl, alkenyl, alkynyl as well as benzyl esters are studied in order to minimize steric effects close to the helicate core. The ^1^H NMR dimerization constants for the monomer–dimer equilibrium show some solvent dependent influence of the side chains on the dimer stability. In the dimer, the ability of the hydrocarbyl ester groups to aggregate minimizes their contacts with the solvent molecules. Due to this, most solvophobic alkyl groups show the highest dimerization tendency followed by alkenyls, alkynyls and finally benzyls. Furthermore, trends within the different groups of compounds can be observed. For example, the dimer is destabilized by internal double or triple bonds due to π–π repulsion. A strong indication for solvent supported London dispersion interaction between the ester side groups is found by observation of an even/odd alternation of dimerization constants within the series of *n*‐alkyls, *n*‐Ω‐alkenyls or *n*‐Ω‐alkynyls. This corresponds to the interaction of the parent hydrocarbons, as documented by an even/odd melting point alternation.

## Graphical abstract

**A stabilizing effect**: Clear trends can be found in the seemingly chaotic distribution of dimerization constants of hierarchically assembled helicates in DMSO, revealing the stabilizing effect of solvent‐supported London dispersion effects.

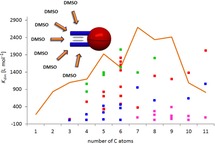

## Introduction

In solids, weak interactions between molecules are most important for the properties of the respective bulk materials. They direct the orientation of molecules towards each other and thus control structures and their properties.^1^ In biological systems weak, interactions are crucial for the spatial arrangement of molecular entities and, thus, the function of for example, biopolymers like polynucleotides or proteins.^2^ Weak noncovalent contacts between molecules can be easily observed in the crystal by using X‐ray diffraction methods.^3^ However, in solution the most dominating interaction a molecule undergoes is the one with the surrounding solvent.^4^ Furthermore, although all weak interactions are based in principle on polarity effects, there is a huge conceptual difference in the nature of for example, hydrogen bonds, π‐based interactions, dispersion interactions or solvophobicity/‐philicity as well as steric effects.^5^

**Figure 1:**
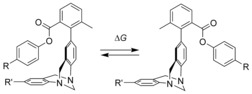
Wilcox molecular torsion balance.

One approach for the study of weak interactions in solution follows a concept which has been promoted in the 1990s by Wilcox. He introduced a simple “molecular torsion balance” to be evaluated by NMR spectroscopy (Figure 1).^6^ Considering that this pioneering work the “Wilcox system” has been frequently used to study different aspects of weak intramolecular interactions.^7^ In addition several other torsion balances following the same basic principle have been described.^8^

**Figure 2:**
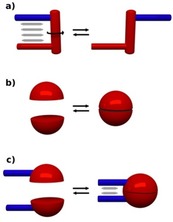
Different concepts for the development of “molecular balances”.

Molecular torsion balances in general are molecules that can adopt two different conformational states with only one having two groups in close proximity for interactions (Figure 2 a). However, it has been pointed out that in solution the weak interactions are dominated/enforced by cohesive solvent effects. This means that the differences in the energy of the two states of the balance are mainly due to the minimization or maximization of the contact surface with the solvent.^9^

The homo‐ or heterodimerization of appropriate species may serve as a tool to develop alternative kinds of molecular balances (Figure 2 b,c). If the energetics of the central dimerizing unit are well understood, it is possible to study the contribution of side‐chain interactions on the dimerization. This has been utilized by Schneider,^10^ Cockroft^11^ or Schreiner^12^ in order to evaluate interactions even as weak as dispersion forces in solution.^13^

**Scheme 1:**
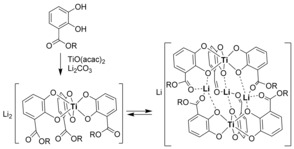
Hierarchical formation of dimeric triple‐lithium bridged titanium(IV) catecholate helicates and the equilibrium between monomer and dimer structures observed in solution.

Since 2005, we have been studying the chemistry of hierarchically formed helicates,^14^ which are essentially dimeric triple‐lithium bridged bis(titanium(IV) triscatecholates).^15^ In solution, a monomer–dimer equilibrium can be observed and its energetics can be accurately determined by NMR spectroscopy (Scheme 1).^16^

In a recent study, we could show that alkyl ester substituents have a distinct influence on the dimerization behaviour in DMSO, which in many cases can be explained by steric, electronic and solvation effects of the side chains. For a special example with “space filling” isopropyl ester groups, it even was possible to get a strong indication of the influence of London dispersion interactions as an important driving force to stabilize the dimer.^17^

**Figure 3:**
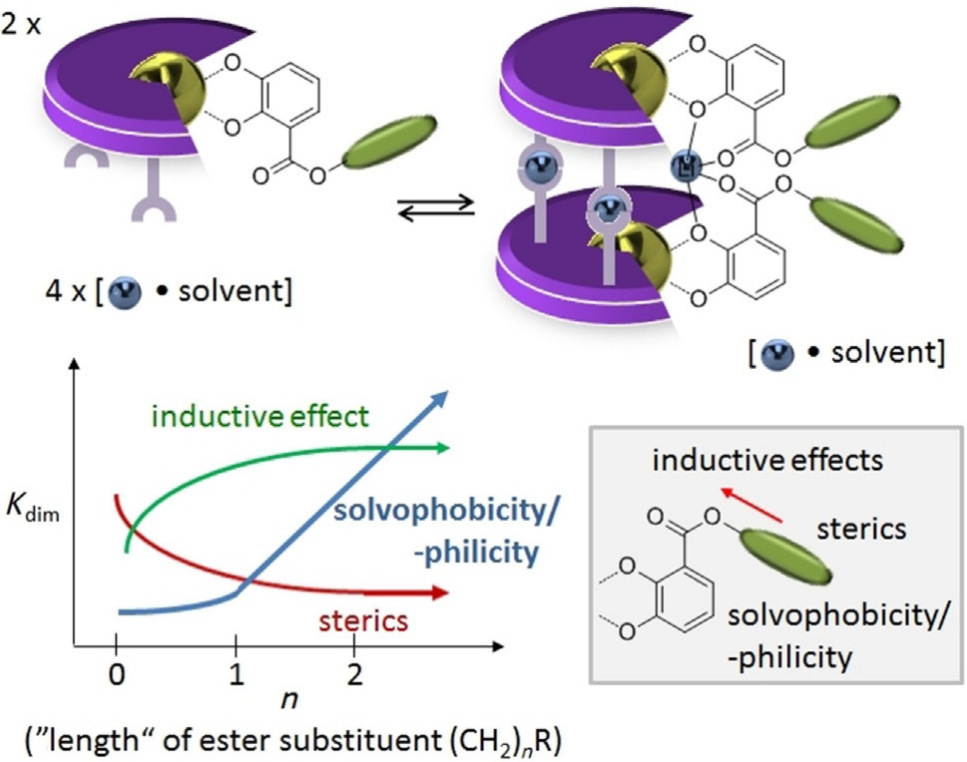
Different effects influencing the dimerization behavior to form hierarchically assembled helicates with hydrocarbyl ester substituted ligands in solution.

In the present study, we aim to use hierarchical helicates for the estimation of solvophobic/‐philic effects of hydrocarbyl substituents in DMSO to be able to determine the relative strength of hydrocarbyl solvation depending on small structural changes. Figure 3 schematically summarizes effects which are important during the dimerization process.

In principle, the stability of the main core of the hierarchical helicates depends more on the binding strength of lithium in the interior of the central complex moiety than compared to the solvation of lithium cation. In this case, some main factors play an important role:

1) The carbonyl moiety attached to the catecholate is highly influential. Esters are the best donors for lithium coordination [*K*_dim_ ${}^{[D{}_{4}]\mathrm{MeOH}}$ (methyl ester)=32 000 L mol^−1^] compared to ketones [*K*_dim_ ${}^{[D{}_{4}]\mathrm{MeOH}}$ (methyl ketone)=3.700 L mol^−1^] and the even weaker aldehyde [*K*_dim_ ${}^{[D{}_{4}]\mathrm{MeOH}}$ =10 L mol^−1^].^16^

2) The ability of the solvent to solvate lithium cations is crucial to destabilize the dimers. Solvents that strongly bind lithium like DMSO easily remove the lithium from the dimer and lead to a high amount of monomer in solution [*K*_dim_ ${}^{[D{}_{6}]\mathrm{DMSO}}$ (methyl ester)=175 L mol^−1^]^17^, whereas less well coordinating solvents like [D_4_]MeOH [*K*_dim_ (methyl ester)=32 000 L mol^−1^], [D_3_]CH_3_CN [*K*_dim_ (methyl ester)=47 000 L mol^−1^] or [D_6_]acetone [*K*_dim_ (methyl ester)=65 000 L mol^−1^] populate the dimer.

3) The kind of central metal ion controls the charge of the triscatecholate complexes. In case of titanium(IV) ions the triscatecholate complex possesses a double negative charge while in case of gallium(III) the complex is triple negative. Due to the strong electrostatic attraction of the lithium cations gallium(III) forms much more stable dimers [*K*_dim_ ${}^{[D{}_{4}]\mathrm{MeOH}}$ (aldehyde)=200 000 L mol^−1^] in solution compared to titanium(IV) [*K*_dim_ ${}^{[D{}_{4}]\mathrm{MeOH}}$ (aldehyde)=10 L mol^−1^].^16^ A study with related 8‐oxoquinolinate ligands reveals that negatively charged monomeric complexes are required to form the dimers. Charge neutral monomers do not form dimers.^18^

4) Substituents at the aromatic units of the catechol may act as electron donors or acceptors and, thus, alter the donor ability of the catecholate as well as the carbonyl oxygen atoms towards lithium cations.^19^

5) Substituents at the carbonyl of the ligand may destabilize the dimer due to their bulkiness.^20^ However, one borderline example has been found in which the dimer is highly favored by a bulky ester side chain due to destabilization of the monomer.^21^

6) The carbonyl substituents have different inductive donor or acceptor abilities and thus modulate the electron density at the carbonyl oxygen. For example, due to the stronger inductive donor effect the ethyl ester substituted complex shows a higher dimer stability (*K*_dim_ ${}^{[D{}_{6}]\mathrm{DMSO}}$ =830 L mol^−1^) compared to the methyl ester (*K*_dim_ ${}^{[D{}_{6}]\mathrm{DMSO}}$ =175 L mol^−1^).^17^

7) In the dimer the contact surface of side chains with the solvent is minimized and thus solvophobic effects will favour the dimer.^17^

8) Weak attractive “through‐space” interactions between the substituents (e.g., dispersion forces) may be a stabilizing factor in favor of the dimer. However, such weak interactions in most cases are hidden by the stronger ones described before.^17^

**Figure 4:**
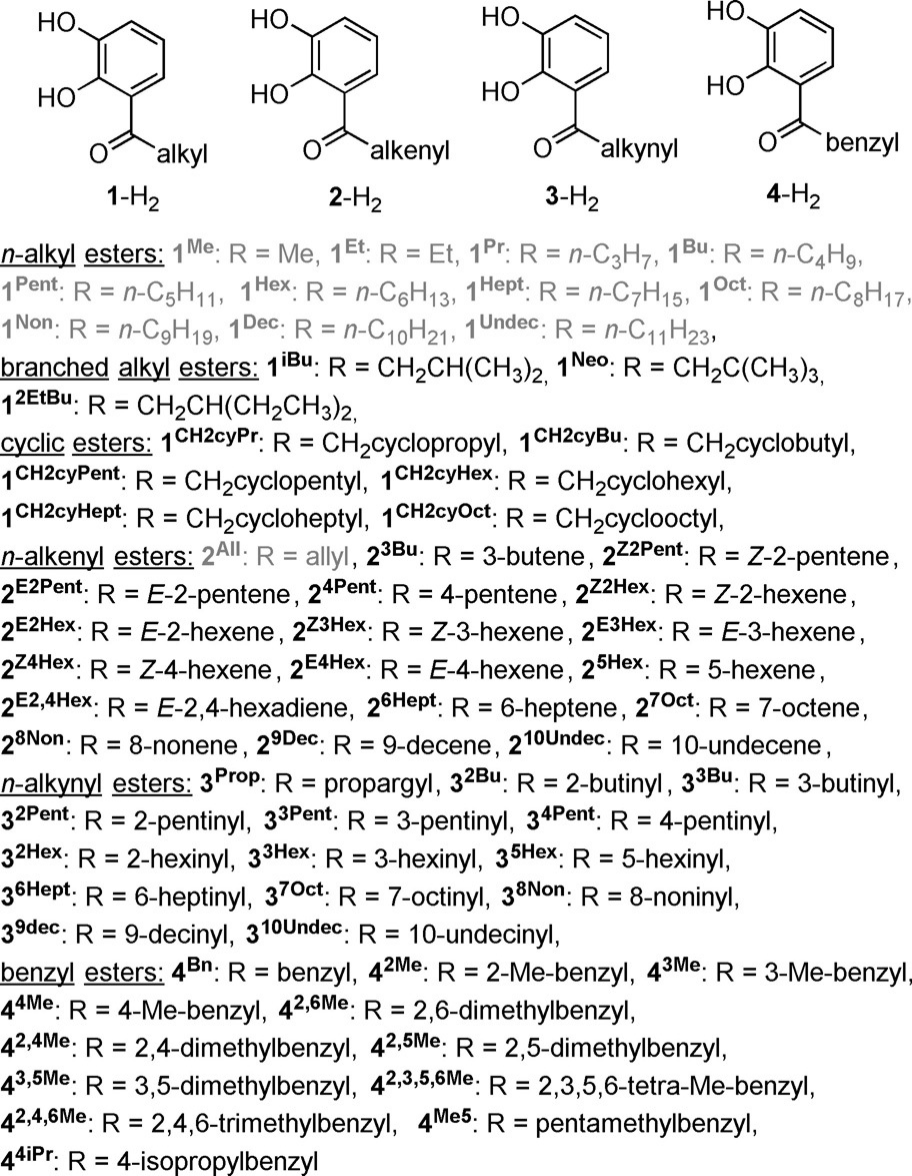
Ester catechol derivatives for hierarchically assembled helicates Li[Li_3_(**L**_3_Ti)_2_]. The derivatives shown in grey have already been described earlier.^16^, ^17^

In the present study the focus will be on the influence of primary hydrocarbyl substituents at ester catecholate ligands on the dimerization in DMSO solution as described under points 5–7 and the influence of the “external” cation will be briefly discussed as well. The influences of steric as well as inductive effects are minimized by choosing primary ester substituents with at least three carbon atoms in order to focus on the solvent influence. As shown in Figure 3, solvent interactions become strong with groups that are not buried in the groove of the helicate. At the same time, steric and inductive effects are minimized in this case. The focus will be on alkyl‐, alkenyl‐ and alkynyl‐ as well as benzyl‐substituted catechol esters as ligands (Figure 4).

## Results and Discussion

### Computational considerations

It has been mentioned above that in this study, in order to focus on the solvent influence, both steric and inductive effects are minimized by choosing primary OCH_2_R ester substituents.

**Figure 5:**
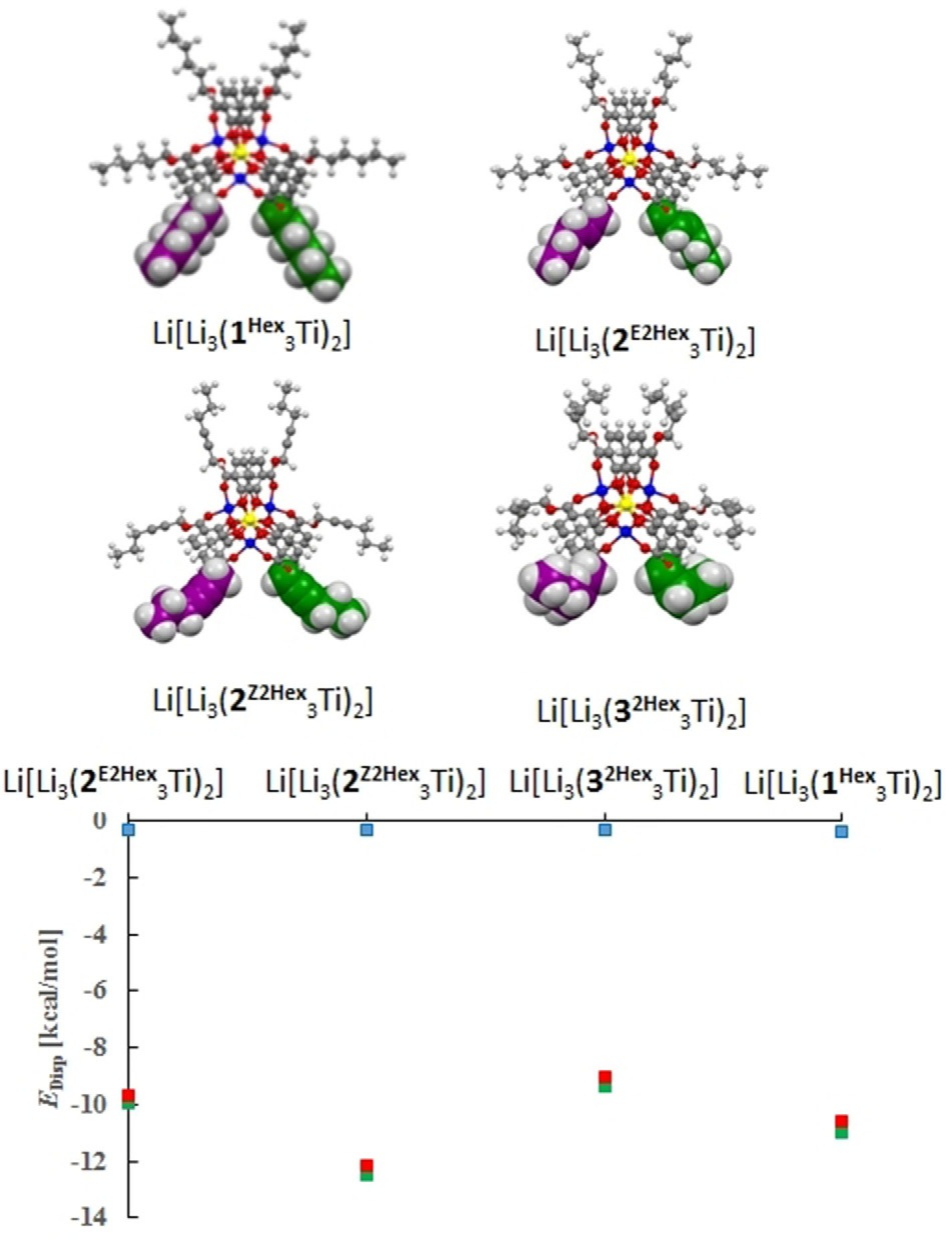
Minimized structures (RHF 6‐31G(d)) of Li[Li_3_(**1^Hex^**_3_Ti)_2_], Li[Li_3_(**2^Z2Hex^**_3_Ti)_2_], Li[Li_3_(**2^E2Hex^**_3_Ti)_2_] and Li[Li_3_(**3^2Hex^**_3_Ti)_2_] as well as the computed intramolecular dispersion energies for the dimeric complexes *E*_Disp B_ (green) as the interaction between neighboring aromatic catecholates and ester side chains of the two complex units. *E*_Disp C_ (red) is reported for interaction between the ester substituents of the first and aromatic catecholates of the second complex unit and the interaction between the aromatic catecholates of the two units. *E*_Disp A_ (blue) is reported for the interaction between neighboring ester side chains of the units.

Initially, some computational investigations on the selected dimeric complexes Li[Li_3_(**1^Hex^**_3_Ti)_2_], Li[Li_3_(**2^Z2Hex^**_3_Ti)_2_], Li[Li_3_(**2^E2Hex^**_3_Ti)_2_] and Li[Li_3_(**3^2Hex^**_3_Ti)_2_] have been performed.^22^ The starting geometries were built by using the crystal structure of Li[Li_3_(**1^Me^**_3_Ti)_2_] as framework and subsequently optimized (RHF 3–21G*). The resulting structures were then further refined employing a larger basis set (RHF 6‐31G*). Three dispersion interaction energies *E*_Disp A_, *E*_Disp B_ and *E*_Disp C_ were calculated.^23^ *E*_Disp A_ (blue squares in Figure 5) measures only the weak interactions between the hydrocarbon substituents of the ester side chains of the two complex units in the dimer. *E*_Disp B_ (green squares in Figure 5) includes the ring fragments of the aromatic catecholates in addition to the hydrocarbon substituents included in the former calculation. *E*_Disp C_ (red squares in Figure 5) is defined as the difference between *E*_Disp A_ and *E*_Disp B_ and, thus, measures the weak interactions between the ring fragments of one complex unit and the ring fragments as well as the side chains of the other. It was found that in the gas phase, as expected, no significant dispersion interactions occur between the hydrocarbon side chains of the ester substituents in case of primary esters. However, strong London dispersion interactions between the substituents and aromatic catecholate units significantly contribute to the stability of the dimer (Figure 5).

### Experimental studies

The catechol ester ligands as well as their complexes Li[Li_3_(**L**_3_Ti)_2_] were prepared as described before.^16^, ^17^, ^24^ Monomers as well as dimers were observed by proton NMR spectroscopy in [D_6_]DMSO at a concentration of 2×10^−3^ mol L^−1^ and the corresponding dimerization constants were obtained by integration. For the determination of reliable data it is essential that the quality of the [D_6_]DMSO used in all studies is the same. The water content of the used samples was determined to be 0.12±0.04 mol L^−1^.^17^ In this context, it also would be of interest to study heteroleptic complexes to evaluate interactions between different kinds of substituents. However, mixed‐ligand complexes only can be prepared as mixtures with statistical composition, so that no reliable data can be obtained on inter‐substituent interactions.16d Attempts to specifically obtain heterodimers by mixing of “complementary” titanium(IV) triscatecholates were not successful yet.

### The role of the “external” cation

Prior to a systematic study of the substituent effects, the influence of the “external cation” on the dimerization constant has been determined.

Therefore, a series of complex salts M[Li_3_(**1^Me^**_3_Ti)_2_] (M=Li, Na, K, Rb, Cs) with the methyl ester catecholates was prepared and the dimerization constants have been measured in [D_6_]DMSO to be *K*_dim_=175 L mol^−1^ (M=Li), 40 L mol^−1^ (M=Na, K, Cs) and 50 L mol^−1^ (M=Rb). All dimerization constants with a cation M different to lithium are very similar but lower than the one obtained for the all‐lithium salt. Thus, the fourth cation does not have a direct influence on the dimerization process. In case of an external lithium cation, the effective concentration of lithium is higher and the formation of the dimer is favored.

### The influence of the ester substituents

All complexes Li[Li_3_(**L**_3_Ti)_2_] with ligands **1**–**4** shown in Figure 4 were prepared and spectroscopically characterized.

### Thermodynamic investigations and structural considerations

**Figure 6:**
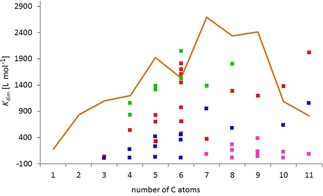
Distribution of the dimerization constants of primary hydrocarbyl substituted ester complexes Li[Li_3_(**L**_3_Ti)_2_] in [D_6_]DMSO depending on the number of C‐atoms of the substituent with branched alkyl (green), *n*‐alkenyl (red), *n*‐alkynyl (blue) and benzyl derivatives (magenta). The already reported data of the *n*‐alkanes are used as standard and are shown as an orange line.^17^

The dimerization constants of all compounds listed in Figure 4 have been determined in [D_6_]DMSO under the standardized conditions. As a rough measure of the “size” of the ester substituents the number of carbon atoms in the ester side chain has been selected. In Figure 6, the obtained dimerization constants of the monomer dimer equilibria are listed against the number of the carbon atoms of the ligand ester side groups (see also Table 1). On the first view, the distribution of data points seems to be more or less chaotic. However, clear trends are observed upon a more detailed look. For example, it is found that the possible maximum of *K*_dim_ increases with the “size” of the side chain.

**Table 1:** Dimerization constants *K*_dim_ [L mol^−1^] of the complexes Li[Li_3_(**L**_3_Ti)_2_] in [D_6_]DMSO at room temperature.

**L**=	*K*_dim_ [L/mol]	**L**=	*K*_dim_ [L mol^− 1^]
**1^Me^**	175±20^[a]^	**1^Et^**	830±100^[a]^
**1^Pr^**	1095±135^[a]^	**1^Bu^**	1195±145^[a]^
**1^Pent^**	1920±240^[a]^	**1^Hex^**	1530±190^[a]^
**1^Hept^**	2690±345^[a]^	**1^Oct^**	2330±300^[a]^
**1^Non^**	2410±300^[a]^	**1^Dec^**	1080±125^[a]^
**1^Undec^**	810±90^[a]^	**1** ^**iBu**^	1050±130
**1^Neo^**	1380±170	**1^2EtBu^**	1530±190
**1^CH2^** ^**cyPr**^	830±100	**1^CH2^** ^**cyBu**^	1310±160
**1^CH2^** ^**cyPent**^	2040±260	**1^CH2^** ^**cyHex**^	1380±170
**1^CH2^** ^**cyHept**^	2600±330	**1^CH2^** ^**cyOct**^	4960±650
**2^All^**	40±5	**2^3Bu^**	540±65
**2^Z2Pent^**	700±80	**2^E2Pent^**	330±35
**2^4Pent^**	930±110	**2^Z2Hex^**	970±115
**2^E2Hex^**	710±85	**2^Z3Hex^**	1610±200
**2^E3Hex^**	1450±180	**2^Z4Hex^**	1700±215
**2^E4Hex^**	1530±190	**2^5Hex^**	1810±225
**2^E2, 4Hex^**	480±40^[b]^	**2^6Hept^**	370±40
**2^7Oct^**	1290±160	**2^8Non^**	1200±145
**2^9Dec^**	1380±170	**2^10Undec^**	2020±255
**3^Prop^**	10±1	**3^2Bu^**	20±2
**3^3Bu^**	175±20	**3^2Pent^**	30±3
**3^3Pent^**	230±25	**3^4Pent^**	420±50
**3^2Hex^**	20±2	**3^3Hex^**	360±40
**3^5Hex^**	455±50	**3^6Hept^**	950±115
**3^7Oct^**	580±65	**3^8Non^**	750±90
**3^9Dec^**	640±75	**3^10Undec^**	1060±130
**4^Bn^**	90±8	**4^2Me^**	20±2
**4^3Me^**	270±30	**4^4Me^**	170±20
**4^2, 6Me^**	50±4	**4^2, 4Me^**	140±15
**4^2, 5Me^**	80±8	**4^3, 5Me^**	390±45
**4^2, 3, 5, 6Me^**	90±9	**4^2, 4, 6Me^**	20±2
**4^Me5^**	110±10	**4^4^** ^**iPr**^	130±15

[a] Ref. 17; [b] Ref. 16d.

Figure 6 shows the *K*_dim_ of the *n*‐alkane derivatives as orange line.^17^ The regions in which *K*_dim_ of the branched primary alkyl (green), *n*‐alkenyl (red), *n*‐alkynyl (blue) and benzyl derivatives (magenta) are located reveal that the *n*‐alkyl groups possess the highest dimerization tendency closely followed by the branched primary alkyl moieties with decreasing *K*_dim_ for *n*‐alkenes>*n*‐alkynes>benzyl esters.

### Complexes Li[Li_3_(1_3_Ti)_2_] with alkyl substituted esters

The *n*‐alkyl ester derivatives of Li[Li_3_(**1**_3_Ti)_2_] and some crystal structures thereof have been described before.^17^ The trend of the dimer stability was explained by an electronic inductive effect increasing from the methyl Li[Li_3_(**1^Me^**_3_Ti)_2_] to ethyl Li[Li_3_(**1^Et^**_3_Ti)_2_] and propyl ester Li[Li_3_(**1^Pr^**_3_Ti)_2_]. With longer chain length this effect can be neglected. However, with longer alkyls the chain reaches out into the solvent leading to strong solvophobic effects favoring aggregation with a maximum of *K*_dim_ for the heptyl ester Li[Li_3_(**1^Hept^**_3_Ti)_2_]. With even longer chain length *K*_dim_ drops again due to an unfavorable entropic contribution.^17^

**Figure 7:**
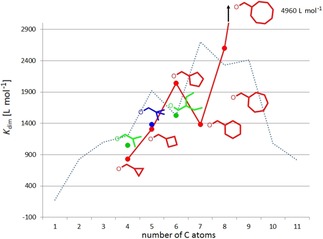
Dimerization constants of primary branched alkyl derivatives of Li[Li_3_(**L**_3_Ti)_2_] in comparison to the corresponding *n*‐alkyl esters (dotted blue line).

Following this, branched primary alkyl esters have been investigated (Figure 7). Hereby, the isobutyl substituted Li[Li_3_(**1** ^***i*****Bu**^_3_Ti)_2_] (*K*_dim_=1050 L mol^−1^) behaves very similar in its stability in [D_6_]DMSO to the corresponding *n*‐propyl Li[Li_3_(**1^Pr^**_3_Ti)_2_] (*K*_dim_=1100 L mol^−1^)^17^ and close to the *n*‐butyl derivative Li[Li_3_(**1^Bu^**_3_Ti)_2_] (*K*_dim_=1200 L mol^−1^).^17^ The dimerization trend increases switching to the neopentyl Li[Li_3_(**1^Neo^**_3_Ti)_2_] (*K*_dim_=1380 L mol^−1^) and then to the 2‐ethylbutyl ester Li[Li_3_(**1^2E*t*Bu^**_3_Ti)_2_] (*K*_dim_=1530 L mol^−1^). The increase of the dimerization constants is attributed to a growing contact surface with the solvent resulting in a stronger influence of the solvophobicity of alkyl groups towards DMSO enforcing alkyl aggregation.

A similar trend is also observed with the cycloalkyl methyl ester substituted complexes. The *K*_dim_ value raises dramatically from the cyclopropyl methyl Li[Li_3_(**1^CH2^** ^**cyPr**^_3_Ti)_2_] (*K*_dim_=830 L mol^−1^) to the cyclooctyl methyl derivative Li[Li_3_(**1^CH2^** ^**cyOct**^_3_Ti)_2_] (*K*_dim_=4960 L mol^−1^). The extraordinarily high dimer stability of the latter is attributed to the large “volume” of the cyclooctyl group opening up the possibility of strong substituent–substituent interactions, thus dramatically reducing the contact with solvent molecules upon dimerization. The *K*_dim_ of Li[Li_3_(**1^CH2^** ^**cyBu**^_3_Ti)_2_] (*K*_dim_=1310 L mol^−1^) is in the same region as the one observed for the neopentyl ester Li[Li_3_(**1^Neo^**_3_Ti)_2_], indicating a similar “size and shape” of the two different substituents. In addition, a drop of *K*_dim_ within the series of cycloalkyl methyl derivatives is observed for the cyclohexyl methyl complex Li[Li_3_(**1^CH2^** ^**cyHex**^_3_Ti)_2_] (*K*_dim_=1380 L mol^−1^). The same decrease of *K*_dim_ has already been observed for the analogous secondary ester of cyclohexanol within the series of cycloalkanol esters.^17^ This is attributed to the special conformational properties of the cyclohexyl group related to ring inversion.

### Complexes Li[Li_3_(2_3_Ti)_2_] with alkenyl substituted esters

**Figure 8:**
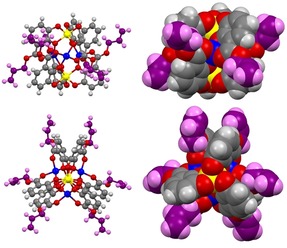
Structure of the anion [Li_3_(**2^All^**_3_Ti)_2_]^−^ as found in the crystal. Top: “side view” (orthogonal to the Ti–Ti axis), bottom: “top view” down the Ti–Ti axis, left: ball and stick representation, right CPK representation. Yellow: Ti, blue: Li, grey: C, white: H, red: O, the allyl groups are shown in purple.

A series of alkenyl derivatives Li[Li_3_(**2**_3_Ti)_2_] has been prepared and characterized. It was possible to obtain a crystal structure of Na[Li_3_(**2^All^**_3_Ti)_2_] (Figure 8).

The core of the dimer is similar as described before for analogous hierarchically formed helicates.^16^, ^17^, ^18^, ^19^, ^20^, ^21^ It is noticed that there is no direct contact between the allyl groups (closest distance: 5.3 Å). The methylene units are still buried in the groove of the helicate showing short distances to the neighboring catecholates (CH⋅⋅⋅C_arom_=2.9–3.3 Å), whereas the terminal alkene slightly sticks out of the vicinity of the dinuclear coordination compound. However, close distances between the internal sp^2^ hybridized C_alkene_ and C_catechol_ atoms as low as 3.5 Å are observed and should result in some π–π repulsion.^25^

**Figure 9:**
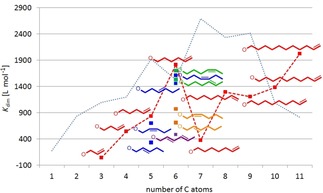
Dimerization constants of *n*‐alkenyl derivatives Li[Li_3_(**2**_3_Ti)_2_] in comparison to the corresponding *n*‐alkyl esters (dotted blue line).

The dimerization constants of the alkene complexes Li[Li_3_(**2**_3_Ti)_2_] are presented in Table 1 and in Figure 9.

In general, alkanes are nearly insoluble in DMSO (e.g., *n*‐pentane: 3.5 g L^−1^ at 20–30 °C), whereas the corresponding alkenes show a higher solubility (e.g., pentenes: 71 g L^−1^ at 20–30 °C).^26^ This already gives a rough indication of the relative solvophobicity of alkanes versus alkenes in DMSO.

The difference can be observed in comparing the stability of dimeric *n*‐alkene substituted complexes Li[Li_3_(**2**_3_Ti)_2_] with the corresponding *n*‐alkanes Li[Li_3_(**1**_3_Ti)_2_]. As a trend the alkenyl ester helicates possess lower dimerization constants as the corresponding alkanyl groups in [D_6_]DMSO. Due to the lower solvophobicity of the alkenyl groups, the dimer is less stabilized than the monomer or the other way around the monomer is better solvated in case of alkenyls than in case of alkyls.

The following trends are observed within the series of alkenyl esters Li[Li_3_(**2**_3_Ti)_2_]:

1) The red squares in Figure 9 represent the dimerization constants of the *n*‐alkenyl substituted complexes Li[Li_3_(**2**_3_Ti)_2_] with terminal double bonds. The simplest, the allyl derivative Li[Li_3_(**2^All^**_3_Ti)_2_] shows a very low K_dim_=40 L mol^−1^ (for comparison Li[Li_3_(**1^Pr^**_3_Ti)_2_] *K*_dim_=1095 L mol^−1^). This can not only be attributed to the lower solvophobicity of the allyl compared to the propyl group. It is rather expected that there is a strong repulsion between the alkene unit and the neighboring catechol of the second complex moiety (π–π repulsion,^27^ see also discussion of crystal structure). The trend of relatively low dimerization constants compared to the alkanes is also observed for the other complexes with 2‐alkenyl substituents (Li[Li_3_(**2^E2Pent^**_3_Ti)_2_]: *K*_dim_=330 L mol^−1^, Li[Li_3_(**2^Z2Pent^**_3_Ti)_2_]: *K*_dim_=700 L mol^−1^, Li[Li_3_(**2^E2Hex^**_3_Ti)_2_]: *K*_dim_=710 L mol^−1^, and Li[Li_3_(**2^Z2Hex^**_3_Ti)_2_]: *K*_dim_=970 L mol^−1^).

2) Starting from the allyl derivative, the dimerization constants of the helicates with terminal double bonds increase with the chain length up to *K*_dim_=1810 L mol^−1^ for Li[Li_3_(**2^5Hex^**_3_Ti)_2_]. Similar trends are observed for shifting of the double bond “outwards” from 2‐pentenyl Li[Li_3_(**2^Z2Pent^**_3_Ti)_2_] and Li[Li_3_(**2^E2Pent^**_3_Ti)_2_] to 3‐hexenyl esters Li[Li_3_(**2^Z3Hex^**_3_Ti)_2_] and Li[Li_3_(**2^E3Hex^**_3_Ti)_2_] (Figure 8).

Li[Li_3_(**2^6Hept^**_3_Ti)_2_] (K_dim_=370 L mol^−1^) shows a dramatic decrease of the dimerization tendency which rises again for Li[Li_3_(**2^7Oct^**_3_Ti)_2_] (*K*_dim_=1290 L mol^−1^). For the 8‐nonene Li[Li_3_(**2^8Non^**_3_Ti)_2_] (*K*_dim_=1200 L mol^−1^) as well as the 9‐decene complex Li[Li_3_(**2^9Dec^**_3_Ti)_2_] (*K*_dim_=1380 L mol^−1^), the constants have a similar value, whereas they rise to *K*_dim_=2020 L mol^−1^ for Li[Li_3_(**2^10Undec^**_3_Ti)_2_].

The high dimerization constant of Li[Li_3_(**2^10Undec^**_3_Ti)_2_] may be explained considering an enthalpy/entropy compensation.^17^ With short alkane chains, the dimerization constants rise due to their solvophobicity. At the same time, the repulsion between the double bonds and the catecholates is reduced with every methylene unit added in the substituent. With long chain length (nonene, decene), the solvophobicity results in chain aggregation and entropically originated reduction of *K*_dim_. In the undecenyl complex Li[Li_3_(**2^10Undec^**_3_Ti)_2_], the double bond is far away from the central helicate and can interact well with the solvent resulting in an increase of the dimerization constant due to a lower influence of the unfavorable entropic contribution at the terminus of the ester chain.

The heptenyl derivative Li[Li_3_(**2^6Hept^**_3_Ti)_2_] is exceptional in the series of Ω‐*n*‐alkenes. However, a similar drop of K_dim_ has been observed with *n*‐hexyl in the series of *n*‐alkanes.

3) In case of internal double bonds, it is found that the *E*‐configurated derivatives show a lower *K*_dim_ as observed for the *Z*‐isomers (e.g., Li[Li_3_(**2^E2Pent^**_3_Ti)_2_]: *K*_dim_=330 L mol^−1^ L, Li[Li_3_(**2^Z2Pent^**_3_Ti)_2_]: *K*_dim_=700 L mol^−1^).

### Complexes Li[Li_3_(3_3_Ti)_2_] with alkynyl substituted esters

**Figure 10:**
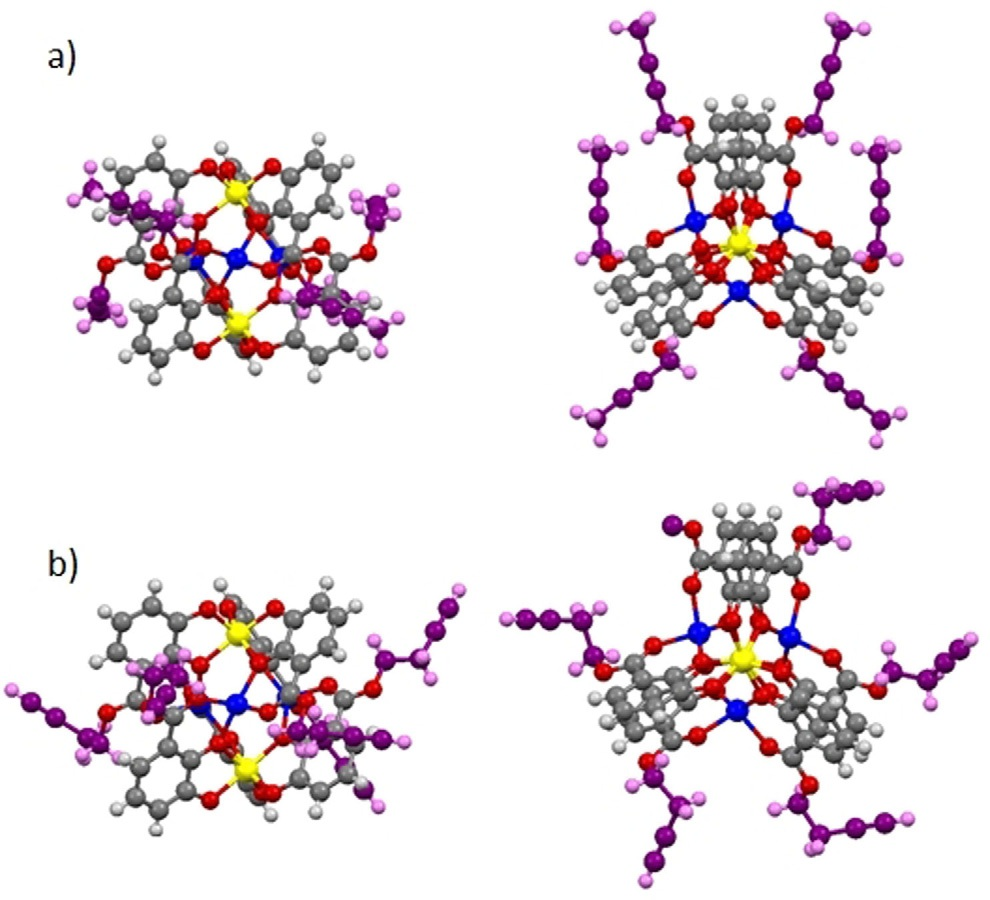
Structure of the anions [Li_3_(**3^2Bu^**_3_Ti)_2_]^−^ (a) and [Li_3_(**3^3Bu^**_3_Ti)_2_]^−^ (b) as found in the crystal. Left: “side view”, right: “top view” down the Ti–Ti axis. Yellow: Ti, blue: Li, grey: C, white: H, red: O, the allyl groups are shown in purple.

Alkynyl ester substituted complexes Li[Li_3_(**3**_3_Ti)_2_] were prepared and the crystal structures of [Li_3_(**3^2Bu^**_3_Ti)_2_]^−^ and [Li_3_(**3^3Bu^**_3_Ti)_2_]^−^ have been determined (Figure 10). Again, the central triple lithium bridged triscatecholate titanium(IV) complex units possess the same structural features as observed for many related examples before.^16^, ^17^, ^18^, ^19^, ^20^, ^21^ However, the strict separation of neighboring alkynyl substituents in the crystal structure is remarkable. Close contacts of the internal methylene groups of the side chains with the catecholate aromatic units force the outer part of the alkynyl substituents away from each other. Closest H⋅⋅⋅H contacts are observed for [Li_3_(**3^2Bu^**_3_Ti)_2_]^−^: *d*(OCH_2_⋅⋅⋅H_2_CO)=5.2 Å and *d*(OCH_2_⋅⋅⋅H_3_C)=3.5 Å and for [Li_3_(**3^3Bu^**_3_Ti)_2_]^−^: *d*(OCH_2_⋅⋅⋅H_2_CO)=3.6 Å.

As described in the computational considerations, dispersion effects between the ester substituents and the catechols seem to stabilize the dimer while corresponding interactions between the side chains are negligible.

However, the alkyl–aryl interactions are weakened in case of neighboring electron clouds at the ester (double or triple bonds). The proximity of the π systems results in some degree of repulsion between the π electrons.

**Figure 11:**
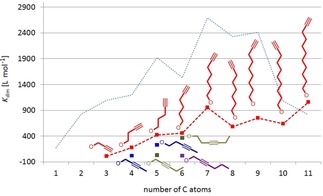
Dimerization constants in [D_6_]DMSO of n‐alkynyl derivatives Li[Li_3_(**3**_3_Ti)_2_] in comparison to the corresponding *n*‐alkyl esters (blue dotted line).

The determination of the dimerization constants of the alkynes reveals lower constants as observed for the alkenes corresponding to the lower solvophobicity of alkynes in DMSO (Figure 11). For the alkynes with terminal alkyne moieties an increase of the dimerization constant with the chain length is observed. As observed for the corresponding alkenes, no drop of the dimerization constants is found with longer chain length. The destabilizing entropic contribution to *ΔG* which is observed for the *n*‐alkanes^17^ seems not to be present in case of terminal “solvophilic” groups.

It is remarkable that all complexes with triple bond in 2‐position, Li[Li_3_(**3^Prop^**_3_Ti)_2_], Li[Li_3_(**3^2Bu^**_3_Ti)_2_], Li[Li_3_(**3^2Pent^**_3_Ti)_2_] and Li[Li_3_(**3^2Hex^**_3_Ti)_2_], show about the same dimerization constant (*K*_dim_=20–30 L mol^−1^). The same is observed at higher values for the 3‐alkynes Li[Li_3_(**3^3Bu^**_3_Ti)_2_], Li[Li_3_(**3^3Pent^**_3_Ti)_2_] (*K*_dim_=230 L mol^−1^) and Li[Li_3_(**3^3Hex^**_3_Ti)_2_] (*K*_dim_=360 L mol^−1^). This indicates that here the π–π repulsion between alkynes or between alkyne and catechol is a dominating interaction for the stability of the dimers. However, this destabilizing effect decreases with the distance of the triple bond to the central helicate moiety.

### Complexes Li[Li_3_(4_3_Ti)_2_] with benzyl substituted esters

**Figure 12:**
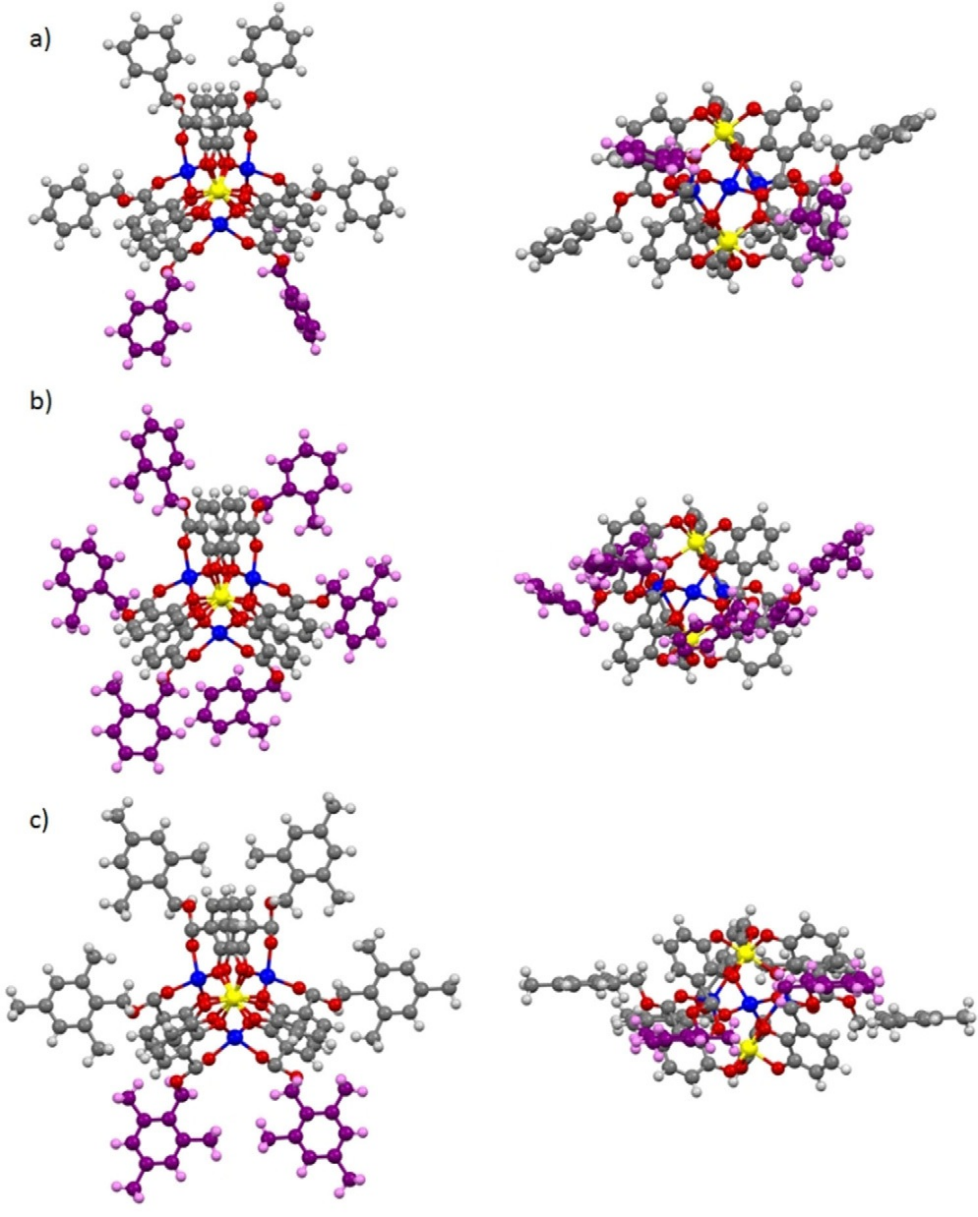
Structure of the anions [Li_3_(**4^Bn^**_3_Ti)_2_]^−^ (a), [Li_3_(**4^2Me^**_3_Ti)_2_]^−^ (b) and [Li_3_(**4^2^** ^, **4**, **6Me**^_3_Ti)_2_]^−^ (c) as found in the crystal. For [Li_3_(**4^2^** ^, **4**, **6Me**^_3_Ti)_2_]^−^ only one of the disordered enantiomers is shown. Left: “side view”, right: “top view” down the Ti–Ti axis. Yellow: Ti, blue: Li, grey: C, white: H, red: O, two benzyl groups are shown in purple.

The benzyl ester complexes Li[Li_3_(**4**_3_Ti)_2_] have been prepared and the derivatives Li[Li_3_(**4^Bn^**_3_Ti)_2_], Na[Li_3_(**4^2Me^**_3_Ti)_2_] and Li[Li_3_(**4^2, 4, 6Me^**_3_Ti)_2_] were structurally characterized (Figure 12). In the crystal of Li[Li_3_(**4^2, 4, 6Me^**_3_Ti)_2_] the enantiomeric left (*ΛΛ*) and right handed forms (*ΔΔ*) are severely disordered (see Supporting Information). For [Li_3_(**4^Bn^**_3_Ti)_2_]^−^, it is observed that the benzyl groups seem to avoid contact to each other, whereas again close contact is found between the benzylic position and catechol units of the other complex moiety. Distances CH_2_⋅⋅⋅C_cat_ as low as *d*=2.82 Å are observed. In [Li_3_(**4^2Me^**_3_Ti)_2_]^−^, all six methylbenzyl units adopt different orientations showing the that no interaction occurs between those groups which would induce some preferential orientation. For [Li_3_(**4^2, 4, 6Me^**_3_Ti)_2_]^−^, all six aromatics are parallel to the plane of the lithium atoms showing close CH_Me_⋅⋅⋅CH_Me_ contacts of 2.9 Å.

**Figure 13:**
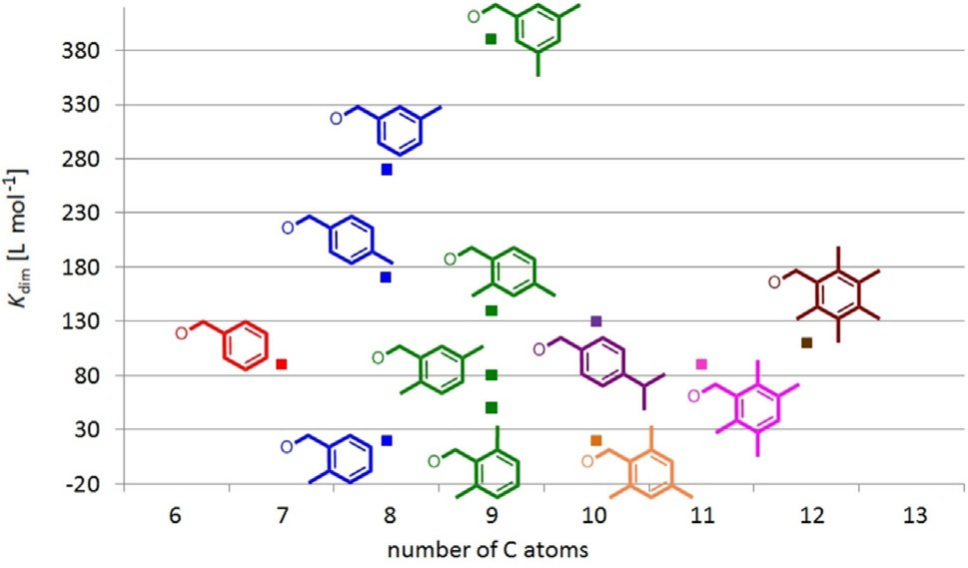
Dimerization constants in [D_6_]DMSO of benzyl derivatives Li[Li_3_(**4**_3_Ti)_2_].

Simple aromatic compounds (benzene, mesitylene) are miscible with DMSO.^26^ Aromatic units therefore do not show solvophobic behavior in this solvent. Consequently, the dimerization constants of the hierarchical helicates Li[Li_3_(**4**_3_Ti)_2_] drop dramatically showing that the side chains of the monomer are well solvated and do not tend to aggregate (Figure 13). Somewhat surprisingly are the dimerization constants of the complexes with methyl groups in 3‐position of the aromatic units in Li[Li_3_(**4^3Me^**_3_Ti)_2_] and Li[Li_3_(**4^3, 5Me^**_3_Ti)_2_], which indicate that here some weak attractions may occur between neighboring substituents.

## Conclusions

In this work, a systematic approach to study solvophobic effects in DMSO as a weak interaction of the solvent with hydrocarbyl ester groups of hierarchically formed helicates is described. In order to minimize steric effects only primary alcohols are introduced as esters.

**Figure 14:**
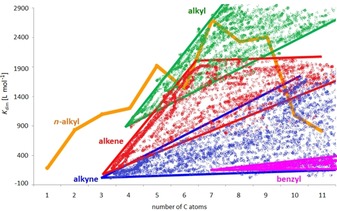
Domains in which the dimerization constants of alkyl (green), alkenyl (red), alkynyl (blue) and benzyl ester (magenta) substituted complexes Li[Li_3_(**L**_3_Ti)_2_] are observed.

One general trend is immediately clear when investigating the dimerization constants of the many examples prepared in this study. Alkyl esters in general possess higher dimerization constants in [D_6_]DMSO than alkenes, followed by alkynes and finally benzyl groups. This strongly correlates with the solubility of the respective compound classes in DMSO (alkanes<alkenes<alkynes<aromatics, Figure 14).^26^

Within the different classes of compounds more subtle trends can be observed like better dimer stabilization in case of *Z*‐alkenes compared to *E*‐alkenes. It is also found that, in case of internal double or triple bonds, destabilization occurs, which is due to π–π repulsion between the substituents and between the substituents and catechol aromatics.

**Figure 15:**
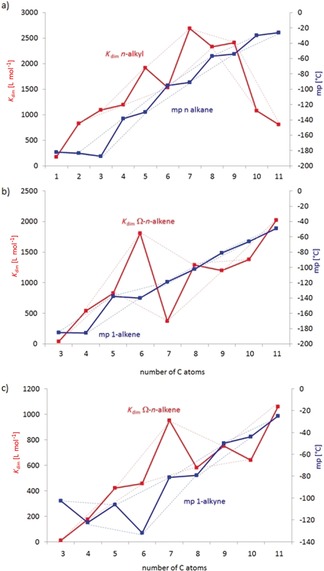
Comparison of the progression of the dimerization constants of *n*‐alkyl (a), Ω‐*n*‐alkenyl (b) and Ω‐*n*‐alkynyl (c) substituted complexes Li[Li_3_(**L**_3_Ti)_2_] (red lines) with the melting points of the corresponding hydrocarbons (blue lines). The dashed lines connect neighboring pairs of even or odd numbered side chains.

However, some additional weak interactions, which are not obvious, seem to be hidden under the ones described above. The melting points of *n*‐alkanes as well as terminal *n*‐alkenes and *n*‐alkynes show some even/odd alternating behavior with progression of the chain length. This is explained by different molecular packing (chain–chain interaction) of the compounds in the 3D crystal lattice.^28^ The shape of the melting point curves of the alkanes, alkenes or alkynes are different to the ones of the corresponding catechol ester complexes (Figure 15). However, observation of a corresponding even/odd alternating behavior of the dimerization constants of the *n*‐hydrocarbyl derivatives (although in some cases opposite to the melting point behavior of the parent compounds) indicates some significant interactions between the alkyl chains depending on the even versus odd number of carbon atoms.

Although computational gas‐phase models indicate virtually no interaction between the substituents, in DMSO solution solvophobicity compresses the hydrocarbyl chains together and, thus, stabilizes the dimer. Thus, in the present case, we directly observe solvent‐supported London dispersion interactions between neighboring hydrocarbyl groups.

## Experimental Section

### Crystallographic data

CCDC https://www.ccdc.cam.ac.uk/services/structures?id=doi:10.1002/chem.201904639 {[Li_3_(**2^All^**_3_Ti)_2_]^−^, [Li_3_ **3^2Bu^**_3_Ti)_2_]^−^, [Li_3_ **3^3Bu^**_3_Ti)_2_]^−^, C_84_H_60_Li_3_O_24_Ti_2_, Li[Li_3_(**4^2,4,6Me^**_3_Ti)_2_], Na[Li_3_(**4^2Me^**_3_Ti)_2_]} contain the supplementary crystallographic data for this paper. These data are provided free of charge by http://www.ccdc.cam.ac.uk/.

## Conflict of interest

The authors declare no conflict of interest.

## Supporting information

As a service to our authors and readers, this journal provides supporting information supplied by the authors. Such materials are peer reviewed and may be re‐organized for online delivery, but are not copy‐edited or typeset. Technical support issues arising from supporting information (other than missing files) should be addressed to the authors.

Supplementary
