# Supplementary material for: Shedding Light on the Interactions of Hydrocarbon Ester Substituents upon Formation of Dimeric Titanium(IV) Triscatecholates in DMSO Solution
Source: Chemistry. 2020 Jan 22;26(6):1396–405. doi: 10.1002/chem.201904639 (PMC7027801; doi:10.1002/chem.201904639)
Supplement: Supplementary file 1 — Supplementary [file CHEM-26-1396-s001.pdf]

CHEMISTRY
A European Journal

Supporting Information

Shedding Light on the Interactions of Hydrocarbon Ester Substituents upon Formation of Dimeric Titanium(IV) Triscatecholates in DMSO Solution

A. Carel N. Kwamen,[a] Marcel Schlottmann,[a] David Van Craen,[a] Elisabeth Isaak,[a] Julia Baums,[a] Li Shen,[a] Ali Massomi,[a] Christoph Räuber,[a] Benjamin P. Joseph,[a] Gerhard Raabe,[a] Christian Göb,[b] Iris M. Oppel,[b] Rakesh Puttreddy,[c] Jas S. Ward,[c] Kari Rissanen,[c] Roland Fröhlich,[d] and Markus Albrecht*[a]

chem_201904639_sm_miscellaneous_information.pdf

Table of Content

1. Materials and Methods S2
2. Preparation of ligands and complexes S2
3. X-ray rystallographic studies S40
4. Computational considerations S42
5. References S43

1. Materials and Methods

2,3-Dihydroxybenzoic acid was obtained from Sigma Aldrich, thionyl chloride from Acros Organics, triethylamine from Alfa Aesar. All solvents were used after distillation. DMSO-d_6 (99.9%, with 0.05% TMS as internal standard) was purchased from Cambridge Isotope Laboratories. NMR spectra were obtained from Mercury 300 as well as Varian VNMRS 400 and 600 NMR spectrometers. The compounds were analytical characterized on a LTQ Orbitrap XL for ESI-MS. IR measurements were done on a Perkin-Elmer 100 spectrometer and elemental analysis was measured with a Heraeus CHN-O-Rapid. Melting points were determined with a BÜCHI B-540 melting point instrument and are reported uncorrected.

General esterification procedure:

2,3-Dihydroxybenzoic acid (1 eq.) is converted into the corresponding acid chloride via refluxing in thionyl chloride (30 eq.) for 3 hours. The excess of thionyl chloride is removed under reduced pressure and the remaining acid chloride is used without further purification in the next step. The acid chloride is dissolved in chloroform (4/5 of the total amount of chloroform used in this step) and a mixture of the corresponding alcohol (5 eq.) and triethylamine (5 eq.) dissolved in chloroform (1/5) is added to the acid chloride solution (total concentration of chloroform = 0.2 M). The reaction mixture is washed with saturated $NaHCO_3$ solution after refluxing overnight. The product is obtained after drying with $MgSO_4$, removal of the solvent, and purification via column chromatography.

General complexation procedure:

Catechol ester ligand (3 eq.) is mixed with $TiO(acac)_2$ (1 eq.) and Li_2CO_3 (1 eq.) and dissolved in methanol. The pure complexes are obtained after stirring this solution for one day and removal of the solvent. No further purification is necessary due to quantitative complexation.

2. Preparation of ligands and complexes

Variation of the cation M in M[$Li_3(1^{Me}{}_3Ti)_2$] (M = Na, K, Rb, Cs)

Na[$Li_3(L^{Me}{}_3Ti)_2$]

The complex is obtained from ligand $\mathbf{1}^{\mathbf{Me}}$-H_2 (100.0 mg, 0.59 mmol) in methanol (25 mL) following the general procedure with a 1:3 mixture of Na_2CO_3 and Li_2CO_3 as base as a red solid (quantitative). **^{1}H NMR** (400 MHz, DMSO-d_6): Dimer (minor component): δ = 6.98 (dd, J = 7.9, 1.6 Hz, 1H, $H_{arom.}$), 6.55 (t, J = 7.9 Hz, 1H, $H_{arom.}$), 6.46 (dd, J = 7.9, 1.6 Hz, 1H, $H_{arom.}$), 2.96 (s, 3H, OCH_3) ppm. Monomer (major component): 6.81 (dd, J = 7.8, 1.6 Hz, 1H, $H_{arom.}$), 6.31 (t, J = 7.8 Hz, 1H, $H_{arom.}$), 6.17 (dd, J = 7.8, 1.6 Hz, 1H, $H_{arom.}$), 3.68 (s, 3H, OCH_3) ppm. **MS** (negative ESI-MS, MeOH): m/z (%) = 1113.1090 (100, [M−Na^+], $C_{48}H_{36}O_{24}Li_3Ti_2^-$, calcd. 1113.1041). **IR** (in KBr): $\tilde{\nu}$ (cm^{-1}) = 3400, 2951, 2289, 2042, 1677, 1440, 1303, 1251, 1198, 1065, 1017, 913, 849, 802, 740, 678. **Elemental analysis** $C_{48}H_{36}O_{24}Li_3NaTi_2 \cdot 4\ H_2O$: calcd. C = 47.71 %, H = 3.67 %; found C = 47.94 %, H = 3.78 %.

K[$Li_3(L^{Me}{}_3Ti)_2$]

The complex is obtained from ligand $\mathbf{1}^{\mathbf{Me}}$-H_2 (100.0 mg, 0.59 mmol) in methanol (25 mL) using the general procedure with a 1:3 mixture of K_2CO_3 and Li_2CO_3 as base as a red solid (quantitative). **^{1}H NMR** (400 MHz, DMSO-d_6): Dimer (minor component): δ = 6.98 (dd, J = 7.9, 1.6 Hz, 1H, $H_{arom.}$), 6.55 (t,

J = 7.9 Hz, 1H, $H_{arom.}$), 6.46 (dd, J = 7.9, 1.6 Hz, 1H, $H_{arom.}$), 2.96 (s, 3H, OCH_3) ppm. Monomer (major component): δ = 6.82 (d, J = 7.8 Hz, 1H, $H_{arom.}$), 6.30 (t, J = 7.8 Hz, 1H, $H_{arom.}$), 6.18 (d, J = 7.8 Hz, 1H, $H_{arom.}$), 3.68 (s, 3H, OCH_3) ppm. **MS** (negative ESI-MS, MeOH): m/z (%) = 1113.1018 (100, [M−K^+], $C_{48}H_{36}O_{24}Li_3Ti_2^-$, calcd. 1113.1041). **IR** (in KBr): ṽ (cm^{-1}) = 3407, 2951, 2321, 2082, 2042, 1901, 1678, 1441, 1304, 1252, 1200, 1065, 1018, 914, 850, 803, 740, 680. **Elemental analysis** $C_{48}H_{36}O_{24}Li_3KTi_2 \cdot 4\ H_2O$: calcd. C = 47.08 %, H = 3.62 %; found C = 47.33 %, H = 3.85 %.

Rb[$Li_3(L^{Me}{}_3Ti)_2$]

The complex is obtained from ligand **1^{Me}**-H_2 (100.0 mg, 0.59 mmol) in methanol (25 mL) following the general procedure with a 1:3 mixture of Rb_2CO_3 and Li_2CO_3 as base as a red solid (quantitative). **^{1}H NMR** (300 MHz, DMSO-d_6): Dimer (minor component): δ = 6.98 (dd, J = 7.9, 1.7 Hz, 1H, $H_{arom.}$), 6.55 (t, J = 7.9 Hz, 1H, $H_{arom.}$), 6.46 (dd, J = 7.9, 1.7 Hz, 1H, $H_{arom.}$), 2.96 (s, 3H, OCH_3) ppm. Monomer (major component): δ = 6.84 (dd, J = 7.8, 1.6 Hz, 1H, $H_{arom.}$), 6.31 (t, J = 7.8 Hz, 1H, $H_{arom.}$), 6.19 (dd, J = 7.8, 1.6 Hz, 1H, $H_{arom.}$), 3.67 (s, 3H, OCH_3) ppm. **MS** (positive ESI-MS, MeOH): m/z (%) = 1282.9279 (100, [M+Rb^+], $C_{48}H_{36}O_{24}Li_3Rb_2Ti_2^+$, calcd. 1282.9266). **IR** (in KBr): ṽ (cm^{-1}) = 3410, 2951, 2316, 2117, 1896, 1677, 1593, 1560, 1439, 1345, 1302, 1251, 1198, 1153, 1064, 1018, 913, 849, 801, 740, 679. **Elemental analysis** $C_{48}H_{36}O_{24}Li_3RbTi_2 \cdot 3\ H_2O$: calcd. C = 46.02 %, H = 3.38 %; found C = 45.95 %, H = 3.39 %.

Cs[$Li_3(L^{Me}{}_3Ti)_2$]

The complex is obtained from the corresponding ligand **L^{Me}**-H_2 (100.0 mg, 0.59 mmol) in methanol (25 mL) with the general procedure with a 1:3 mixture of Cs_2CO_3 and Li_2CO_3 as base as a red solid (quantitative). **^{1}H NMR** (400 MHz, DMSO-d_6): Dimer (minor component): δ = 6.98 (dd, J = 7.9, 1.6 Hz, 1H, $H_{arom.}$), 6.55 (t, J = 7.9 Hz, 1H, $H_{arom.}$), 6.46 (dd, J = 7.9, 1.6 Hz, 1H, $H_{arom.}$), 2.96 (s, 3H, OCH_3) ppm. Monomer (major component): δ = 6.82 (d, J = 7.8 Hz, 1H, $H_{arom.}$), 6.30 (t, J = 7.8 Hz, 1H, $H_{arom.}$), 6.18 (d, J = 7.8 Hz, 1H, $H_{arom.}$), 3.67 (s, 3H, OCH_3) ppm. **MS** (negative ESI-MS, MeOH): m/z (%) = 1113.1080 (100, [M−Cs^+], $C_{48}H_{36}O_{24}Li_3Ti_2^-$, calcd. 1113.1041). **IR** (in KBr): ṽ (cm^{-1}) = 3428, 2952, 2324, 2112, 1902, 1678, 1439, 1302, 1251, 1107, 1153, 1064, 1017, 913, 849, 801, 741, 678. **Elemental analysis** $C_{48}H_{36}O_{24}Li_3CsTi_2 \cdot 2\ H_2O$: calcd. C = 44.96 %, H = 3.14 %; found C = 45.13 %, H = 3.07 %.

Alkyl ester derivatives

Ligands 1-H_2

Isobutyl-2,3-dihydroxybenzoate (1^{iBu}-H_2):

The synthesis of the ligand was achieved with isobutyl alcohol (1.30 g, 17.5 mmol) according to the general procedure. Purification via column chromatography (pentane/ethyl acetate 20:1, R_f = 0.35) gives the product as a colorless oil (81 %, 597 mg, 2.84 mmol). **^{1}H NMR** (600 MHz, $CDCl_3$): δ = 10.99 (s, 1H, OH), 7.39 (dd, 1H, J = 8.0, 1.5 Hz, $H_{arom.}$), 7.11 (dd, 1H, J = 8.0, 1.5 Hz, $H_{arom.}$), 6.80 (t, 1H, J = 8.0 Hz, $H_{arom.}$), 5.65 (s, 1H, OH), 4.13 (d, 2H, J = 6.7 Hz, OCH_2), 2.15-2.05 (m, 1H, CH_2CH), 1.03 (d, 6H, J = 6.7 Hz, CH_3) ppm. **^{13}C NMR** (151 MHz, $CDCl_3$): δ = 170.36 (CO_2CH_2), 148.89 ($C_{arom.}$), 145.01 ($C_{arom.}$), 120.47 ($C_{arom.}$), 119.67 ($C_{arom.}$), 119.14 ($C_{arom.}$), 112.63 ($C_{arom.}$), 71.45 (OCH_2), 27.78 (CH_2CH), 19.08 (CH_3) ppm. **MS** (positive ESI-MS, MeOH, acidified): m/z (%) = 233.0780 (100, [M+Na^+], $C_{11}H_{14}O_4Na^+$, calcd. 233.0784). **IR** (KBr): ṽ (cm^{-1}) = 3466, 3136, 2961, 2731, 2326, 2095, 1997, 1915, 1669, 1466, 1383,

1302, 1263, 1149, 1066, 999, 839, 750. **Elemental analysis** $C_{11}H_{14}O_4$: calcd. C = 62.85 %, H = 6.71 %; found C = 63.24 %, H = 6.99 %.

Neopentyl-2,3-dihydroxybenzoate (1^{Neo}-H_2):

The ligand is synthesized with neopentyl alcohol (1.54 g, 17.5 mmol) according to the general procedure. Column chromatography (pentane/ethyl acetate 20:1, R_f = 0.28) results in the product as a colorless solid (73 %, 575 mg, 2.56 mmol). **M.p.**: 71 C – 75 °C (last solvent used: DCM). ^{1}**H NMR** (600 MHz, $CDCl_3$): δ = 10.97 (bs, 1H, O*H*), 7.40 (dd, 1H, *J* = 8.0, 1.5 Hz, $H_{arom.}$), 7.11 (dd, 1H, *J* = 8.0, 1.5 Hz, $H_{arom.}$), 6.81 (t, 1H, *J* = 8.0 Hz, $H_{arom.}$), 5.71 (bs, 1H, O*H*), 4.04 (s, 2H, OCH_2), 1.04 (s, 9H, CH_3) ppm. ^{13}**C NMR** (151 MHz, $CDCl_3$): δ = 170.36 (CO_2CH_2), 148.91 ($C_{arom.}$), 145.06 ($C_{arom.}$), 120.39 ($C_{arom.}$), 119.71 ($C_{arom.}$), 119.17 ($C_{arom.}$), 112.66 ($C_{arom.}$), 74.63 (OCH_2), 31.57 (CH_2C), 26.48 (CH_3) ppm. **MS** (positive ESI-MS, MeOH, acidified): *m/z* (%) = 247.0932 (100, [M+Na^+], $C_{12}H_{16}O_4Na^+$, calcd. 247.0941). **IR** (KBr): ṽ (cm^{-1}) = 3489, 3128, 2956, 2317, 2201, 2159, 2097, 1917, 1742, 1662, 1463, 1373, 1311, 1267, 1229, 1151, 1065, 995, 925, 842, 796, 747, 696. **Elemental analysis** $C_{12}H_{16}O_4$: calcd. C = 64.27 %, H = 7.19 %; found C = 64.74 %, H = 7.44 %.

2-Ethyl-1-butyl-2,3-dihydroxybenzoate (1^{2EtBu}-H_2):

2-Ethyl-1-butanol (1.79 g, 17.5 mmol) is used for the esterification according to the general procedure. The product is obtained via column chromatography (pentane/ethyl acetate 25:1, R_f = 0.23) as a colorless oil (77 %, 0.641 g, 2.69 mmol). ^{1}**H NMR** (600 MHz, $CDCl_3$): δ = 11.00 (s, 1H, O*H*), 7.36 (dd, 1H, *J* = 8.0, 1.5 Hz, $H_{arom.}$), 7.12-7.09 (m, 1H, $H_{arom.}$), 6.80 (t, 1H, *J* = 8.0 Hz, $H_{arom.}$), 5.62 (s, 1H, O*H*), 4.28 (d, 2H, *J* = 5.7 Hz, OCH_2), 1.70-1.63 (m, 1H, CH_2CH), 1.49-1.42 (m, 4H, CH_2CH_3), 0.95 (t, 6H, *J* = 7.5 Hz, CH_3) ppm. ^{13}**C NMR** (151 MHz, $CDCl_3$): δ = 170.47 (CO_2CH_2), 148.87 ($C_{arom.}$), 145.01 ($C_{arom.}$), 120.47 ($C_{arom.}$), 119.62 ($C_{arom.}$), 119.13 ($C_{arom.}$), 112.68 ($C_{arom.}$), 67.45 (OCH_2), 40.36 (CH_2CH), 23.41 (CH_2CH_3), 11.08 (CH_3) ppm. **MS** (positive ESI-MS, MeOH, acidified): *m/z* (%) = 261.1093 (77, [M+Na^+], $C_{13}H_{18}O_4Na^+$, calcd. 261.1097). **IR** (KBr): ṽ (cm^{-1}) = 3454, 3111, 2938, 2345, 2093, 1668, 1456, 1273, 1151, 743. **Elemental analysis** $C_{13}H_{18}O_4$: calcd. C = 65.53 %, H = 7.61 %; found C = 65.80 %, H = 7.71 %.

Cyclopropylmethyl-2,3-dihydroxybenzoate ($1^{CH2cyPr}$):

Cyclopropyl methanol (1.26 g, 17.5 mmol) is converted according to the general procedure and the product is obtained after column chromatography (pentane/ethyl acetate 18:1, R_f = 0.18) as colorless solid (55 %, 399 mg, 1.92 mmol). **M.p.**: 50°C – 54°C (last used solvent: DCM). ^{1}**H NMR** (600 MHz, $CDCl_3$): δ = 10.98 (s, 1H, O*H*), 7.42 (dd, 1H, *J* = 8.0, 1.4 Hz, $H_{arom.}$), 7.10 (dd, 1H, *J* = 8.0, 1.4 Hz, $H_{arom.}$), 6.80 (t, 1H, *J* = 8.0 Hz, $H_{arom.}$), 5.63 (s, 1H, O*H*), 4.19 (d, 2H, *J* = 7.3 Hz, OCH_2), 1.30-1.23 (m, 1H, CH_2CH), 0.66-0.62 (m, 2H, $H_{cycl.}$), 0.39-0.36 (m, 2H, $H_{cycl.}$) ppm. ^{13}**C NMR** (151 MHz, $CDCl_3$): δ = 170.43 (CO_2CH_2), 148.87 ($C_{arom.}$), 144.98 ($C_{arom.}$), 120.67 ($C_{arom.}$), 119.64 ($C_{arom.}$), 119.10 ($C_{arom.}$), 112.68 ($C_{arom.}$), 70.36 (OCH_2), 9.71 (CH_2CH), 3.42 (2×$C_{cycl.}$) ppm. **MS** (positive ESI-MS, MeOH): *m/z* (%) = 209.0808 (90, [M+H^+], $C_{11}H_{13}O_4^+$, calcd. 209.0808). **IR** (KBr): ṽ (cm^{-1}) = 3839, 3463, 3184, 3084, 3010, 2949, 2659, 2323, 2104, 1995, 1863, 1734, 1667, 1466, 1410, 1356, 1299, 1264, 1147, 1066, 980, 840, 751. **Elemental analysis** $C_{11}H_{12}O_4$: calcd. C = 63.45 %, H = 5.81 %; found C = 63.89 %, H = 6.08 %.

Cyclobutylmethyl-2,3-dihydroxybenzoate ($1^{CH2cyBu}$):

The synthesis of the ligand is achieved with cyclobutyl methanol (1.51 g, 17.5 mmol) according to the general procedure. Purification via column chromatography (pentane/ethyl acetate 18:1, R_f = 0.15) results in the product as colorless oil (83 %, 646 mg, 2.91 mmol). **^{1}H NMR** (600 MHz, $CDCl_3$): δ = 10.98 (s, 1H, O*H*), 7.38 (dd, 1H, *J* = 8.0, 1.3 Hz, $H_{arom.}$), 7.10 (dd, 1H, *J* = 8.0, 1.3 Hz, $H_{arom.}$), 6.80 (t, 1H, *J* = 8.0 Hz, $H_{arom.}$), 5.63 (s, 1H, O*H*), 4.32 (d, 2H, *J* = 6.6 Hz, OCH_2), 2.81-2.72 (m, 1H, CH_2CH), 2.17-2.09 (m, 2H, $H_{cycl.}$), 2.02-1.83 (m, 4H, $H_{cycl.}$) ppm. **^{13}C NMR** (151 MHz, $CDCl_3$): δ = 170.46 (CO_2CH_2), 148.86 ($C_{arom.}$), 145.01 ($C_{arom.}$), 120.55 ($C_{arom.}$), 119.65 ($C_{arom.}$), 119.11 ($C_{arom.}$), 112.63 ($C_{arom.}$), 69.12 (OCH_2), 33.98 (CH_2CH), 24.70 ($2\times C_{cycl.}$), 18.45 ($C_{cycl.}$) ppm. **MS** (positive ESI-MS, MeOH): *m/z* (%) = 245.0786 (100, [$M+Na^+$], $C_{12}H_{14}O_4Na^+$, calcd. 245.0784). **IR** (KBr): $\tilde{\nu}$ (cm^{-1}) = 3882, 3463, 3137, 2951, 2661, 2325, 2094, 1996, 1913, 1667, 1465, 1393, 1263, 1149, 1063, 987, 907, 840, 750. **Elemental analysis** $C_{12}H_{14}O_4$: calcd. C = 64.85 %, H = 6.35 %; found C = 65.03 %, H = 6.66 %.

Cyclopentylmethyl-2,3-dihydroxybenzoate ($1^{CH2cyPent}$):

Cyclopentyl methanol (1.75 g, 17.5 mmol) is converted to the corresponding ligand using the general synthetic procedure and obtained after column chromatography (pentane/ethyl acetate 20:1, R_f = 0.20) as colorless oil (74 %, 615 mg, 2.61 mmol). **^{1}H NMR** (600 MHz, $CDCl_3$): δ = 10.99 (s, 1H, O*H*), 7.37 (dd, 1H, *J* = 8.0, 1.4 Hz, $H_{arom.}$), 7.10 (dd, 1H, *J* = 8.0, 1.4 Hz, $H_{arom.}$), 6.80 (t, 1H, *J* = 8.0 Hz, $H_{arom.}$), 5.63 (s, 1H, O*H*), 4.24 (d, 2H, *J* = 7.0 Hz, OCH_2), 2.40-2.31 (m, 1H, CH_2CH), 1.87-1.80 (m, 2H, $H_{cycl.}$), 1.71-1.55 (m, 4H, $H_{cycl.}$), 1.39-1.31 (m, 2H, $H_{cycl.}$) ppm. **^{13}C NMR** (151 MHz, $CDCl_3$): δ = 170.43 (CO_2CH_2), 148.87 ($C_{arom.}$), 145.00 ($C_{arom.}$), 120.53 ($C_{arom.}$), 119.63 ($C_{arom.}$), 119.11 ($C_{arom.}$), 112.67 ($C_{arom.}$), 69.36 (OCH_2), 38.48 (CH_2CH), 29.34 ($2\times C_{cycl.}$), 25.34 ($2\times C_{cycl.}$) ppm. **MS** (positive ESI-MS, MeOH, acidified): *m/z* (%) = 259.0943 (100, [$M+Na^+$], $C_{13}H_{16}O_4Na^+$, calcd. 259.0941). **IR** (KBr): $\tilde{\nu}$ (cm^{-1}) = 3454, 3130, 2946, 2079, 1910, 1668, 1461, 1271, 1148, 991, 842, 743. **Elemental analysis** $C_{13}H_{16}O_4$: calcd. C = 66.09 %, H = 6.83 %; found C = 66.18 %, H = 6.88 %.

Cyclohexylmethyl-2,3-dihydroxybenzoate ($1^{CH2cyHex}$):

Cyclohexyl methanol (2.00 g, 17.5 mmol) is esterified according to the general procedure. Purification with column chromatography (pentane/ethyl acetate 22:1, R_f = 0.32) results in the product as colorless solid (76 %, 669 mg, 2.67 mmol). **M.p.:** 80°C – 82°C (last used solvent: DCM). **^{1}H NMR** (600 MHz, $CDCl_3$): δ = 10.99 (s, 1H, O*H*), 7.38 (dd, 1H, *J* = 8.0, 1.4 Hz, $H_{arom.}$), 7.10 (dd, 1H, *J* = 8.0, 1.4 Hz, $H_{arom.}$), 6.79 (t, 1H, *J* = 8.0 Hz, $H_{arom.}$), 5.65 (s, 1H, O*H*), 4.16 (d, 2H, *J* = 6.3 Hz, OCH_2), 1.85-1.73 (m, 5H, $H_{cycl.}$), 1.73-1.67 (m, 1H, $H_{cycl.}$), 1.33-1.24 (m, 2H, $H_{cycl.}$), 1.24-1.16 (m, 1H, $H_{cycl.}$), 1.11-1.02 (m, 2H, $H_{cycl.}$) ppm. **^{13}C NMR** (151 MHz, $CDCl_3$): δ = 170.40 (CO_2CH_2), 148.87 ($C_{arom.}$), 145.01 ($C_{arom.}$), 120.51 ($C_{arom.}$), 119.64 ($C_{arom.}$), 119.11 ($C_{arom.}$), 112.67 ($C_{arom.}$), 70.52 (OCH_2), 37.11 ($C_{cycl.}$), 29.64 ($2\times C_{cycl.}$), 26.29 ($C_{cycl.}$), 25.63 ($2\times C_{cycl.}$) ppm. **MS** (negative ESI-MS, MeOH, acidified): *m/z* (%) = 249.1178 (49, [$M-H^+$], $C_{14}H_{17}O_4^-$, calcd. 249.1132). **IR** (KBr): $\tilde{\nu}$ (cm^{-1}) = 3470, 3098, 2923, 2333, 2048, 1657, 1454, 1255, 1160, 990, 844, 729. **Elemental analysis** $C_{14}H_{18}O_4$: calcd. C = 67.18 %, H = 7.25 %; found C = 67.40 %, H = 7.41 %.

Cycloheptylmethyl-2,3-dihydroxybenzoate ($1^{CH2cyHept}$-H_2):

The ligand is synthesized with cycloheptyl methanol (647 mg, 5.05 mmol) according to the general procedure. Column chromatography (pentane/ethyl acetat 5:1, R_f = 0.45) results in the product as

colorless oil (18 %, 48 mg, 0.2 mmol). ^{1}**H NMR** (400 MHz, $CDCl_3$): δ = 10.99 (s, 1H, O*H*), 7.38 (dd, 1H, *J* = 8.0, 1.6 Hz, $H_{arom.}$), 7.11 (m, 1H, $H_{arom.}$), 6.80 (t, 1H, *J* = 8.0 Hz, $H_{arom.}$), 5.66 (s, 1H, O*H*), 4.06 (d, 2H, OCH_2), 2.04-1.93 (m, 1H, CH_2CH), 1.84-1.46 (m, 10H, $H_{cycl.}$), 1.37-1.26 (m, 2H, $H_{cycl.}$) ppm. ^{13}**C NMR** (101 MHz, $CDCl_3$): δ = 170.40 (CO_2CH_2), 148.85 ($C_{arom.}$), 145.00 ($C_{arom.}$), 120.46 ($C_{arom.}$), 119.62 ($C_{arom.}$), 119.09 ($C_{arom.}$), 112.65 ($C_{arom.}$), 70.54 (OCH_2), 38.54 (CH_2CH) , 30.92 (2x$C_{cycl.}$), 28.45 (2x$C_{cycl.}$), 26.22 (2x$C_{cycl.}$) ppm. **MS** (EI, 70 eV): *m/z* (%) = 264.1 (100, [M^+], $C_{15}H_{20}O_4^+$)). **IR** (KBr): ṽ (cm^{-1}) = 3431, 2925, 2253, 2127, 1657, 1465, 1385, 1316, 1234, 1158, 1025, 825, 764, 628

Cyclooctylmethyl-2,3-dihydroxybenzoate ($1^{CH2cyOct}$-H_2):

The ligand is synthesized with cyclooctyl methanol (830 mg, 5.84 mmol) according to the general procedure. Column chromatography (pentane/ethyl acetat 5:1, R_f = 0.50) results in the product as colorless oil (20 %, 56 mg, 0.2 mmol). ^{1}**H NMR** (400 MHz, $CDCl_3$): δ = 10.99 (s, 1H, O*H*), 7.37 (dd, 1H, *J* = 8.0, 1.5 Hz, $H_{arom.}$), 7.10 (dd, 1H, *J* = 8.0, 1.5 Hz, $H_{arom.}$), 6.80 (t, 1H, *J* = 8.0 Hz, $H_{arom.}$), 5.65 (s, 1H, O*H*), 4.06 (d, 2H, OCH_2), 7.04-1.98 (m, 1H, CH_2CH), 1.78-1.35 (m, 14H, $H_{cycl.}$) ppm. ^{13}**C NMR** (101 MHz, $CDCl_3$): δ = 170.40 (CO_2CH_2), 148.86 ($C_{arom.}$), 145.01 ($C_{arom.}$), 120.46 ($C_{arom.}$), 119.65 ($C_{arom.}$), 119.10 ($C_{arom.}$), 112.66 ($C_{arom.}$), 70.93 (OCH_2), 36.78 (CH_2CH), 29.20 (2x$C_{cycl.}$), 26.84 (2x$C_{cycl.}$), 26.28 (2x$C_{cycl.}$), 25.27 ($C_{cycl.}$) ppm. **MS** (EI, 70 eV): *m/z* (%) = 278.1 (100, [M^+], $C_{16}H_{22}O_4^+$). **IR** (KBr): ṽ (cm^{-1}) = 3151, 2919, 2855, 2256, 2123, 1910, 1668, 1590, 1464, 1393, 1308, 1233, 1155, 998, 824, 752. **Elemental analysis** $C_{16}H_{22}O_4$: calcd. C = 69.04 %, H = 7.97 %; found C = 69.13 %, H = 8.08 %.

Complexes Li[$Li_3(1_3Ti)_2$]

Li[$Li_3(1^{iBu}{}_3Ti)_2$]:

The complex is synthesized with ligand $\mathbf{1}^{iBu}$-H_2 (105 mg, 0.50 mmol) in methanol (25 mL) according to the general procedure und is obtained as red solid (quantitative). ^{1}**H NMR** (400 MHz, MeOH-*d*$_4$): Only dimer: δ = 7.13 (dd, 1H, *J* = 7.1, 2.7 Hz, $H_{arom.}$), 6.63-6.54 (m, 2H, $H_{arom.}$), 3.38 (dd, 1H, *J* = 10.7, 7.4 Hz, OCH_2), 2.81 (dd, 1H, *J* = 10.7, 6.1 Hz, OCH_2), 1.76-1.65 (m, 1H, CH_2CH), 0.82 (dd, 6H, *J* = 6.6, 5.2 Hz, CH_3) ppm. ^{1}**H NMR** (400 MHz, DMSO-*d*$_6$): Dimer (major component): δ = 6.96 (dd, 1H, *J* = 7.9, 1.6 Hz, $H_{arom.}$), 6.49 (t, 1H, *J* = 7.9 Hz, $H_{arom.}$), 6.40 (dd, 1H, *J* = 7.9, 1.6 Hz, $H_{arom.}$), 2.62 (dd, 1H, *J* = 10.6, 6.1 Hz, OCH_2), 1.69-1.58 (m, 1H, CH_2CH), 0.74 (t, 6H, *J* = 6.8 Hz, CH_3) ppm. The other signal of OCH_2 is overlapping with the water signal and is not listed. Monomer (minor component): δ = 6.74 (dd, 1H, *J* = 7.8, 1.6 Hz, $H_{arom.}$), 6.23 (t, 1H, *J* = 7.8 Hz, $H_{arom.}$), 6.08 (dd, 1H, *J* = 7.8, 1.6 Hz, $H_{arom.}$), 3.81 (d, 2H, *J* = 6.6 Hz, OCH_2), 1.92-1.82 (m, 1H, CH_2CH), 0.88 (d, 6H, *J* = 6.7 Hz, CH_3) ppm. **MS** (positive ESI-MS, MeOH): *m/z* (%) = 1379.4186 (100, [M_D+Li^+], $C_{66}H_{72}O_{24}Li_5Ti_2^+$, calcd. 1379.4167). **IR** (KBr): ṽ (cm^{-1}) = 3363, 3069, 2959, 2663, 2313, 2063, 1986, 1899, 1738, 1675, 1594, 1444, 1375, 1295, 1251, 1207, 1064, 1010, 897, 847, 791, 740, 679. **Elemental analysis** $C_{66}H_{72}O_{24}Li_4Ti_2 \cdot 2\ H_2O$: calcd. C = 56.27 %, H = 5.44 %; found C = 56.44 %, H = 5.72 %.

Li[$Li_3(1^{Neo}{}_3Ti)_2$]:

The complex is obtained from the corresponding ligand $\mathbf{1}^{Neo}$-H_2 (112 mg, 0.50 mmol) in methanol (25 mL) with the general procedure as a red solid (quantitative). ^{1}**H NMR** (400 MHz, MeOH-*d*$_4$): Dimer (major component): δ = 7.13 (dd, 1H, *J* = 6.3, 3.4 Hz, $H_{arom.}$), 6.61-6.54 (m, 2H, $H_{arom.}$), 3.42 (d, 1H, *J* = 10.7 Hz, OCH_2), 2.53 (d, 1H, *J* = 10.7 Hz, OCH_2), 0.81 (s, 9H, CH_3) ppm. Monomer (minor component): δ = 7.34 (dd, 1H, *J* = 8.0, 1.5 Hz, $H_{arom.}$), 7.00 (dd, 1H, *J* = 8.0, 1.5 Hz, $H_{arom.}$), 6.75 (t, 1H, *J* = 8.0 Hz,

$H_{arom.}$), 4.04 (s, 2H, OC*H₂*), 1.03 (s, 9H, C*H₃*) ppm. **^{1}H NMR** (400 MHz, DMSO-d_6): Dimer (major component): δ = 6.98 (dd, 1H, *J* = 7.9, 1.5 Hz, $H_{arom.}$), 6.52 (t, 1H, *J* = 7.9 Hz, $H_{arom.}$), 6.41 (dd, 1H, *J* = 7.9, 1.5 Hz, $H_{arom.}$), 2.39 (d, 1H, *J* = 10.5 Hz, OCH_2), 0.76 (s, 9H, CH_3) ppm. The other OCH_2 signal is overlapping with the water signal and is not assigned. Monomer (minor component): δ = 6.76 (dd, 1H, *J* = 7.8, 1.6 Hz, $H_{arom.}$), 6.23 (t, 1H, *J* = 7.8 Hz, $H_{arom.}$), 6.08 (dd, 1H, *J* = 7.8, 1.6 Hz, $H_{arom.}$), 3.71 (s, 2H, OCH_2), 0.89 (s, 9H, CH_3) ppm. **MS** (positive ESI-MS, MeOH): *m/z* (%) = 1463.5115 (100, [M_D+Li^+], $C_{72}H_{84}O_{24}Li_5Ti_2^+$, calcd. 1463.5106). **IR** (KBr): ṽ (cm^{-1}) = 3368, 2957, 2663, 2218, 2070, 1910, 1743, 1677, 1593, 1444, 1370, 1296, 1251, 1209, 1065, 1010, 943, 896, 854, 811, 743, 680. **Elemental analysis** $C_{72}H_{84}O_{24}Li_4Ti_2 \cdot H_2O$: calcd. C = 58.63 %, H = 5.88 %; found C = 58.54 %, H = 6.00 %.

Li[Li$_3$(1^{2EtBu}$_3$Ti)$_2$]:

The conversion of **1^{2EtBu}**-H_2 (119 mg, 0.50 mmol) is performed according to the general procedure in methanol (25 mL). The corresponding complex is obtained as red solid (quantitative). **^{1}H NMR** (400 MHz, MeOH-d_4): Only dimer: δ = 7.07 (dd, 1H, *J* = 7.3, 2.4 Hz, $H_{arom.}$), 6.58-6.47 (m, 2H, $H_{arom.}$), 3.66 (dd, 1H, *J* = 11.1, 5.6 Hz, OCH_2), 2.91 (dd, 1H, *J* = 11.1, 5.3 Hz, OCH_2), 1.36-1.11 (m, 5H, CH_2CH & CH_2CH_3), 0.77 (td, 6H, *J* = 7.3, 5.4 Hz, CH_2CH_3) ppm. **^{1}H NMR** (400 MHz, DMSO-d_6): Dimer (major component): δ = 6.92 (dd, 1H, *J* = 7.8, 1.6 Hz, $H_{arom.}$), 6.47 (t, 1H, *J* = 7.8 Hz, $H_{arom.}$), 6.38 (dd, 1H, *J* = 7.8, 1.6 Hz, $H_{arom.}$), 3.54 (dd, 1H, *J* = 11.1, 6.0 Hz, OCH_2), 2.78 (dd, 1H, *J* = 11.1, 5.0 Hz, OCH_2), 0.70 (td, 6H, *J* = 7.3, 2.7 Hz, CH_2CH_3) ppm. Monomer (minor component): δ = 6.73 (dd, 1H, *J* = 7.8, 1.6 Hz, $H_{arom.}$), 6.23 (t, 1H, *J* = 7.8 Hz, $H_{arom.}$), 6.08 (dd, 1H, *J* = 7.8, 1.6 Hz, $H_{arom.}$), 3.95 (d, 2H, *J* = 5.7 Hz, OCH_2), 0.81 (t, 6H, *J* = 7.4 Hz, CH_2CH_3) ppm. The missing signals of dimer and monomer are overlapping and not assigned. **MS** (negative ESI-MS, MeOH): *m/z* (%) = 1533.6210 (100, [M_D–Li^+], $C_{78}H_{96}O_{24}Li_3Ti_2^-$, calcd. 1533.5736). **IR** (KBr): ṽ (cm^{-1}) = 3362, 3067, 2958, 2874, 2665, 2311, 2071, 1991, 1901, 1742, 1676, 1593, 1518, 1443, 1385, 1294, 1252, 1209, 1065, 1009, 898, 853, 809, 741, 680. **Elemental analysis** $C_{78}H_{96}O_{24}Li_4Ti_2 \cdot 2\ H_2O$: calcd. C = 59.40 %, H = 6.39 %; found C = 59.54 %, H = 6.46 %.

Li[Li$_3$(1^{CH2cyPr}$_3$Ti)$_2$]:

The complex is obtained from the corresponding ligand **1^{CH2cyPr}**-H_2 (104 mg, 0.50 mmol) in methanol (25 mL) as a red solid (quantitative). **^{1}H NMR** (400 MHz, MeOH-d_4): Only dimer: δ = 7.12 (dd, 1H, *J* = 6.5, 3.2 Hz, $H_{arom.}$), 6.59-6.48 (m, 2H, $H_{arom.}$), 3.40 (dd, 1H, *J* = 11.5, 7.5 Hz, OCH_2), 2.88 (dd, 1H, *J* = 11.5, 6.8 Hz, OCH_2), 0.93-0.76 (m, 1H, CH_2CH), 0.44-0.33 (m, 2H, $H_{cycl.}$), 0.17-0.02 (m, 2H, $H_{cycl.}$) ppm. **^{1}H NMR** (400 MHz, DMSO-d_6): Dimer (major component): δ = 7.00 (dd, 1H, *J* = 7.9, 1.6 Hz, $H_{arom.}$), 6.51 (t, 1H, *J* = 7.9 Hz, $H_{arom.}$), 6.42 (dd, 1H, *J* = 7.9, 1.6 Hz, $H_{arom.}$), 2.82 (dd, 1H, *J* = 11.5, 7.0 Hz, OCH_2), 0.88-0.77 (m, 1H, CH_2CH), 0.41-0.34 (m, 2H, $H_{cycl.}$), 0.17-0.01 (m, 2H, $H_{cycl.}$) ppm. Monomer (minor component): δ = 6.78 (dd, 1H, *J* = 7.8, 1.6 Hz, $H_{arom.}$), 6.27 (t, 1H, *J* = 7.8 Hz, $H_{arom.}$), 6.13 (dd, 1H, *J* = 7.8, 1.6 Hz, $H_{arom.}$), 3.93 (d, 2H, *J* = 7.0 Hz, OCH_2), 1.19-1.09 (m, 1H, CH_2CH), 0.52-0.45 (m, 2H, $H_{cycl.}$), 0.34-0.25 (m, 2H, $H_{cycl.}$) ppm. Signals not listed are overlapping and are not assigned. **MS** (positive ESI-MS, MeOH): *m/z* (%) = 1367.3257 (100, [M_D+Li^+], $C_{66}H_{60}O_{24}Li_5Ti_2^+$, calcd. 1367.3228). **IR** (KBr): ṽ (cm^{-1}) = 3660, 3352, 3074, 3009, 2936, 2675, 2194, 2064, 1910, 1741, 1675, 1593, 1443, 1354, 1293, 1254, 1209, 1062, 994, 894, 821, 742, 679. **Elemental analysis** $C_{66}H_{60}O_{24}Li_4Ti_2 \cdot H_2O$: calcd. C = 57.50 %, H = 4.53 %; found C = 57.64 %, H = 4.80 %.

Li[Li$_3$(1^{CH2cyBu}$_3$Ti)$_2$]:

The complex is synthesized in methanol (25 mL) with **1**^{CH2cyBu}-H_2 (111 mg, 0.50 mmol) and is obtained as red solid (quantitative). **^{1}H NMR** (400 MHz, MeOH-d_4): Dimer (major component): δ = 7.10 (dd, 1H, J = 6.9, 2.9 Hz, $H_{arom.}$), 6.59-6.50 (m, 2H, $H_{arom.}$), 3.58 (dd, 1H, J = 11.1, 6.7 Hz, OCH_2), 2.94 (dd, 1H, J = 11.1, 6.1 Hz, OCH_2), 2.44-2.32 (m, 1H, CH_2CH), 1.99-1.63 (m, 6H, $H_{cycl.}$) ppm. Monomer (minor component): δ = 7.32 (dd, 1H, J = 8.0, 1.6 Hz, $H_{arom.}$), 6.99 (dd, 1H, J = 8.0, 1.6 Hz, $H_{arom.}$), 6.73 (t, 1H, J = 8.0 Hz, $H_{arom.}$), 4.31 (d, 2H, J = 6.5 Hz, OCH_2) ppm. Overlapping signals are not assigned. **^{1}H NMR** (400 MHz, DMSO-d_6): Dimer (major component): δ = 6.95 (dd, 1H, J = 7.9, 1.6 Hz, $H_{arom.}$), 6.48 (t, 1H, J = 7.9 Hz, $H_{arom.}$), 6.39 (dd, 1H, J = 7.9, 1.6 Hz, $H_{arom.}$), 3.49 (dd, 1H, J = 11.0, 6.7 Hz, OCH_2), 2.82 (dd, 1H, J = 11.0, 6.0 Hz, OCH_2), 2.37-2.25 (m, 1H, CH_2CH) ppm. Monomer (minor component): δ = 6.77-6.70 (m, 1H, $H_{arom.}$), 6.23 (t, 1H, J = 7.6 Hz, $H_{arom.}$), 6.09 (dd, 1H, J = 7.6, 1.5 Hz, $H_{arom.}$), 4.01 (d, 2H, J = 6.4 Hz, OCH_2), 2.61-2.53 (m, 1H, CH_2CH) ppm. Overlapping signals are not assigned. **MS** (negative ESI-MS, MeOH): m/z (%) = 1437.4276 (100, [M−Li^+], $C_{72}H_{72}O_{24}Li_3Ti_2^-$, calcd. 1437.3858). **IR** (KBr): ṽ (cm^{-1}) = 3363, 2944, 2665, 2196, 2090, 1932, 1742, 1674, 1591, 1554, 1443, 1383, 1346, 1292, 1207, 1064, 1000, 809, 741, 680. **Elemental analysis** $C_{72}H_{72}O_{24}Li_4Ti_2 \cdot 2\ H_2O$: calcd. C = 58.40 %, H = 5.17 %; found C = 58.55 %, H = 5.53 %.

Li[Li_3(1^{CH2cyPent}$_3$Ti)$_2$]:

The complex is formed in methanol (25 mL) with the corresponding ligand **1**^{CH2cyPent}-H_2 (118 mg, 0.50 mmol) and obtained as red solid (quantitative). **^{1}H NMR** (400 MHz, MeOH-d_4): Dimer (major component): δ = 7.08 (dd, 1H, J = 6.9, 2.8 Hz, $H_{arom.}$), 6.60-6.49 (m, 2H, $H_{arom.}$), 3.50 (dd, 1H, J = 10.8, 7.5 Hz, OCH_2), 2.89 (dd, 1H, J = 10.8, 6.5 Hz, OCH_2), 2.04-1.91 (m, 1H, CH_2CH), 1.67-1.44 (m, 6H, $H_{cycl.}$), 1.19-1.08 (m, 2H, $H_{cycl.}$) ppm. Monomer (minor component): δ = 7.31 (dd, 1H, J = 8.0, 1.7 Hz, $H_{arom.}$), 7.01-6.97 (m, 1H, $H_{arom.}$), 6.73 (t, 1H, J = 8.0 Hz, $H_{arom.}$), 4.24 (d, 2H, J = 6.9 Hz, OCH_2) ppm. The other signals are overlapping and are not assigned. **^{1}H NMR** (400 MHz, DMSO-d_6): Dimer (major component): δ = 6.93 (dd, 1H, J = 7.9, 1.6 Hz, $H_{arom.}$), 6.47 (t, 1H, J = 7.9 Hz, $H_{arom.}$), 6.38 (dd, 1H, J = 7.9, 1.6 Hz, $H_{arom.}$), 3.39 (dd, 1H, J = 10.7, 7.4 Hz, OCH_2), 2.75 (dd, 1H, J = 10.7, 6.4 Hz, OCH_2), 1.96-1.83 (m, 1H, CH_2CH) ppm. Monomer (minor component) δ = 6.73 (dd, 1H, J = 7.7, 1.6 Hz, $H_{arom.}$), 6.23 (t, 1H, J = 7.7 Hz, $H_{arom.}$), 6.09 (dd, 1H, J = 7.7, 1.6 Hz, $H_{arom.}$), 3.92 (d, 2H, J = 6.9 Hz, OCH_2), 2.20-2.11 (m, 1H, CH_2CH) ppm. The not assigned signals are overlapping. **MS** (positive ESI-MS, MeOH): m/z (%) = 1535.5084 (100, [M_D+Li^+], $C_{78}H_{84}O_{24}Li_5Ti_2^+$, calcd. 1535.5106). **IR** (KBr): ṽ (cm^{-1}) = 3360, 2947, 2868, 2670, 2326, 2071, 1908, 1742, 1676, 1592, 1444, 1354, 1295, 1208, 1065, 1003, 850, 804, 742, 680. **Elemental analysis** $C_{78}H_{84}O_{24}Li_4Ti_2 \cdot 2\ H_2O$: calcd. C = 59.86 %, H = 5.67 %; found C = 60.11 %, H = 5.89 %.

Li[Li_3(1^{CH2cyHex}$_3$Ti)$_2$]:

Ligand **1**^{CH2cyHex}-H_2 (125 mg, 0.50 mmol) is converted according to the general procedure in methanol (25 mL). The complex is obtained as a red solid (quantitative). **^{1}H NMR** (400 MHz, MeOH-d_4): Dimer (major component): δ = 7.08 (dd, 1H, J = 6.8, 3.0 Hz, $H_{arom.}$), 6.59-6.50 (m, 2H, $H_{arom.}$), 3.40 (dd, 1H, J = 10.8, 7.3 Hz, OCH_2), 2.81 (dd, 1H, J = 10.8, 6.0 Hz, OCH_2), 1.70-1.52 (m, 5H, $H_{cycl.}$), 1.48-1.35 (m, 1H, $H_{cycl.}$), 1.28-1.09 (m, 3H, $H_{cycl.}$), 0.91-0.77 (m, 2H, $H_{cycl.}$) ppm. Monomer (minor component): δ = 7.31 (dd, 1H, J = 8.0, 1.5 Hz, $H_{arom.}$), 6.99 (dd, 1H, J = 8.0, 1.5 Hz, $H_{arom.}$), 6.73 (t, 1H, J = 8.0 Hz, $H_{arom.}$), 4.15 (d, 2H, J = 6.2 Hz, OCH_2) ppm. The other monomer signals are overlapping with the dimer ones and are not listed. **^{1}H NMR** (400 MHz, DMSO-d_6): Dimer (major component): δ = 6.93 (dd, 1H, J = 7.9, 1.6 Hz, $H_{arom.}$), 6.48 (t, 1H, J = 7.9 Hz, $H_{arom.}$), 6.39 (dd, 1H, J = 7.9, 1.6 Hz, $H_{arom.}$), 2.67 (dd, 1H, J = 10.8, 5.9 Hz, OCH_2) ppm. Monomer (minor component): δ = 6.74 (dd, 1H, J = 7.8, 1.5 Hz, $H_{arom.}$), 6.23 (t, 1H, J =

7.8 Hz, $H_{arom.}$), 6.09 (dd, 1H, *J* = 7.8, 1.5 Hz, $H_{arom.}$), 3.85 (d, 2H, *J* = 6.5 Hz, O*CH₂*) ppm. Overlapping signals, which cannot be assigned, are not listed. **MS** (negative ESI-MS, MeOH): *m/z* (%) = 1605.6238 (90, $[M_D–Li^+]$, $C_{84}H_{96}O_{24}Li_3Ti_2^-$, calcd. 1605.5736). **IR** (KBr): ṽ (cm^{-1}) = 3361, 3069, 2923, 2852, 2664, 2318, 2174, 2044, 1932, 1740, 1675, 1594, 1556, 1443, 1381, 1296, 1208, 1066, 1004, 900, 851, 809, 740, 680. **Elemental analysis** $C_{84}H_{96}O_{24}Li_4Ti_2 \cdot 3\ H_2O$: calcd. C = 60.52 %, H = 6.17 %; found C = 60.63 %, H = 6.33 %.

Li[Li$_3$(1^{CH2cyHept}$_3$Ti)$_2$]:

The complex is obtained from the corresponding ligand **1**^{CH2cyHept}-H_2 (40.0 mg, 0.15 mmol) in methanol (25 mL) as a red solid (quantitative). **^{1}H NMR** (400 MHz, DMSO-d_6): Dimer (major component): *δ* = 6.96 (dd, 1H, *J* = 8.0, 1.6 Hz, $H_{arom.}$), 6.51 (t, 1H, *J* = 8.0 Hz, $H_{arom.}$), 6.42 (dd, 1H, *J* = 8.0, 1.6 Hz, $H_{arom.}$) ppm. Monomer (minor component): *δ* = 6.75 (dd, 1H, *J* = 7.9, 1.6 Hz, $H_{arom.}$), 6.25 (t, 1H, *J* = 7.9 Hz, $H_{arom.}$), 6.10 (dd, 1H, *J* = 7.9 Hz, 1.6 Hz, $H_{arom.}$), 3.86 (d, 2H, *J* = 6.8 Hz, OCH_2) ppm. Signals not listed are overlapping and cannot be assigned. **MS** (negative ESI-MS, MeOH): *m/z* (%) = 1689.6884 (100, $[M_D–Li^+]$, $C_{90}H_{108}O_{24}Li_3Ti_2^-$, calcd. 1689.6675). **IR** (KBr): ṽ (cm^{-1}) = 3362, 2918, 2853, 2683, 2324, 2091, 1961, 1831, 1676, 1593, 1562, 1443, 138, 1346, 1296, 1253, 1214, 1153, 1008, 855, 802, 741, 682.

Li[Li$_3$(1^{CH2cyOct}$_3$Ti)$_2$]:

The complex is obtained from the corresponding ligand **1**^{CH2cyOct}-H_2 (45.0 mg, 0.16 mmol) in methanol (25 mL) as a red solid (quantitative). **^{1}H NMR** (400 MHz, DMSO-d_6): Dimer (major component): *δ* = 6.96 (dd, 1H, *J* = 8.0, 1.6 Hz, $H_{arom.}$), 6.51 (t, 1H, *J* = 8.0 Hz, $H_{arom.}$), 6.43 (dd, 1H, *J* = 8.0, 1.6 Hz, $H_{arom.}$), 2.64 (dd, 1H, *J* = 10.6, 6.3 Hz, C*H*) ppm. Monomer (minor component): *δ* = 6.76 (dd, 1H, *J* = 8.0, 1.6 Hz, $H_{arom.}$), 6.26 (t, 1H, *J* = 8.0 Hz, $H_{arom.}$), 6.12 (dd, 1H, *J* = 8.0, 1.6 Hz, $H_{arom.}$), 3.85 (d, 2H, *J* = 6.9 Hz, OCH_2) ppm. Signals not listed are overlapping and cannot be assigned. **MS** (negative ESI-MS, MeOH): *m/z* (%) = 1773.7492 (100, $[M_D–Li^+]$, $C_{96}H_{120}O_{24}Li_3Ti_2^-$, calcd. 1773.7614). **IR** (KBr): ṽ (cm^{-1}) = 3357, 3067, 2917, 2853, 2688, 2434, 2108, 2009, 1939, 1677, 1594, 1561, 1444, 1389, 1344, 1297, 1253, 1215, 1154, 1063, 1010, 945, 891, 854, 810, 743, 683. **Elemental analysis** $C_{96}H_{120}O_{24}Li_4Ti_2 \cdot 3\ H_2O$: calcd. C = 62.82 %, H = 6.92 %; found C = 62.78 %, H = 6.76 %.

Alkenylester ligands

Ligands 2-H$_2$

Prop-2-en-2,3-dihydroxybenzoate (2^{All}-H$_2$)

Allyl alcohol (0.39 mL, 5.84 mmol) is converted into the corresponding ester using a modification of the general procedure. 3 equivalents of the alcohol and 6 equivalents of NEt_3 are used and the reaction mixture was refluxed for 3 days. The product is obtained after purification via column chromatography (pentane/ethyl acetate 20:1, R_f = 0.28) as yellowish oil (68 %, 255 mg, 1.32 mmol). **^{1}H NMR** (400 MHz, $CDCl_3$): *δ* = 10.85 (s, 1H, O*H*), 7.39 (dd, *J* = 8.0, 1.5 Hz, 1H, $H_{arom.}$), 7.11 (dd, *J* = 8.0, 1.5 Hz, 1H, $H_{arom.}$), 6.80 (t, *J* = 8.0 Hz, 1H, $H_{arom.}$), 6.05-5.97 (m, 1H, C*H*), 5.61 (s, 1H, O*H*), 5.41-5.37 (m, 1H, C*H*), 5.27-5.23 (m, 1H, C*H*), 4.81-4.79 (m, 2H, OCH_2) ppm. **^{13}C NMR** (101 MHz, $CDCl_3$): *δ* = 169.97 (*C*O_2CH_2), 148.91 ($C_{arom.}$), 145.01 ($C_{arom.}$), 131.39 (CH_2CH), 120.57 ($C_{arom.}$), 119.84 ($C_{arom.}$), 119.21 ($C_{arom.}$), 118.99 (CHCH_2), 112.38 ($C_{arom.}$), 65.96 (OCH_2) ppm. **MS** (negative and positive ESI-MS, MeOH,

acidified): *m/z* (%) = 193.0517 (100, [M-H$^+$], $C_{10}H_9O_4^-$, calcd. 193.0481), 217.0468 (40, [M+Na$^+$], $C_{10}H_{10}O_4Na^+$, calcd. 217.0477). **IR** (KBr): ṽ (cm^{-1}) = 3469, 3089, 2946, 2738, 2324, 2175, 2082, 1998, 1917, 1670, 1465, 1371, 1300, 1262, 1146, 1067, 988, 930, 839, 750, 697. **Elemental Analysis**: $C_{10}H_{10}O_4$: calcd. C = 61.85 %, H = 5.19 %; found C = 61.19 %, H = 5.38 %.

But-3-en-2,3-dihydroxybenzoate (2^{3Bu}-H_2)

3-Buten-1-ol (280.73 mg, 3.89 mmol) is converted into the corresponding ester using a modification of the general procedure. 3 equivalents of the alcohol and 6 equivalents of NEt_3 are used and the reaction mixture was refluxed for 3 days. The product is obtained after purification via column chromatography (pentane/ethyl acetate 20:1, R_f = 0.3) as colorless oil (40 %, 108 mg, 0.52 mmol). **^{1}H NMR** (600 MHz, $CDCl_3$): δ = 10.90 (s, 1H, O*H*), 7.37 (dd, *J* = 8.0, 1.5 Hz, 1H, $H_{arom.}$), 7.11 (dd, *J* = 8.0, 1.5 Hz, 1H, $H_{arom.}$), 6.80 (t, *J* = 8.0 Hz, 1H, $H_{arom.}$), 5.88-5.85 (m, 1H, C*H*), 5.64 (s, 1H, O*H*), 5.21-5.17 (m, 1H, C*H*), 5.15-5.11 (m, 1H, C*H*), 4.40 (t, *J* = 6.7 Hz, 2H, OCH_2), 2.56-2.52 (m, 2H, CH_2) ppm. **^{13}C NMR** (151 MHz, $CDCl_3$): δ = 170.23 (*C*O_2CH_2), 148.85 ($C_{arom.}$), 145.00 ($C_{arom.}$), 133.50 (CH_2*C*H), 120.56 ($C_{arom.}$), 119.75 ($C_{arom.}$), 119.12 ($C_{arom.}$), 117.79 (CH*C*H_2), 112.51 ($C_{arom.}$), 64.51 (O*C*H_2), 32.94 (*C*H_2) ppm. **MS** (negative ESI-MS, MeOH, acidified): *m/z* (%) = 207.0662 (100, [M-H$^+$], $C_{11}H_{11}O_4^-$, calcd. 207.0656). **IR** (KBr): ṽ (cm^{-1}) = 3881, 3468, 3079, 2966, 2663, 2320, 2088, 1913, 1669, 1466, 1394, 1304, 1263, 1149, 1067, 988, 918, 750. **Elemental Analysis**: $C_{11}H_{12}O_4$: calcd. C = 63.45 %, H = 5.81 %; found C = 63.09 %, H = 5.54 %.

(*Z*)-Pent-2-en-2,3-dihydroxybenzoate (2^{Z2Pent}-H_2)

cis-2-Penten-1-ol (335.35 mg, 3.89 mmol) is converted into the corresponding ester using a modification of the general procedure. 3 equivalents of the alcohol and 6 equivalents of NEt_3 are used and the reaction mixture was refluxed for 3 days. The product is obtained after purification via column chromatography (pentane/ethyl acetate 20:1, R_f = 0.44) as colorless oil (27 %, 78 mg, 0.35 mmol). **^{1}H NMR** (600 MHz, $CDCl_3$): δ = 10.94 (s, 1H, O*H*), 7.38 (dd, *J* = 8.0, 1.5 Hz, 1H, $H_{arom.}$), 7.10 (dd, *J* = 8.0, 1.5 Hz, 1H, $H_{arom.}$), 6.79 (t, *J* = 8.0 Hz, 1H, $H_{arom.}$), 5.76-5.72 (m, 1H, C*H*), 5.68 (s, 1H, O*H*), 5.63-5.60 (m, 1H, C*H*), 4.90 (dd, *J* = 6.9, 1.2 Hz, 2H, OCH_2), 2.21-2.17 (m, 2H, CH_2), 1.03 (t, *J* = 7.5 Hz, 3H, CH_2CH_3) ppm. **^{13}C NMR** (151 MHz, $CDCl_3$): δ = 170.26 (*C*O_2CH_2), 148.87 ($C_{arom.}$), 144.97 ($C_{arom.}$), 138.07 (CH_2*C*H), 122.03 (CH*C*H), 121.85 ($C_{arom.}$), 120.64 ($C_{arom.}$), 119.75 ($C_{arom.}$), 112.55 ($C_{arom.}$), 61.24 (O*C*H_2), 21.01 (CH*C*H_2), 14.08 (CH_2*C*H_3) ppm. **MS** (negative and positive ESI-MS, MeOH, acidified)**:** *m/z* (%) = 221.0823 (50, [M-H$^+$], $C_{12}H_{13}O_4^-$, calcd. 221.0813), 261.1223 (60, [M+K$^+$], $C_{12}H_{14}O_4K^+$, calcd.: 261.1223), 223.0957 (10, [M+H$^+$] $C_{12}H_{15}O_4^+$, calcd. 223.0972). **IR** (KBr): ṽ (cm^{-1}) = 3472, 3190, 3027, 2966, 2876, 2739, 2458, 2325, 2204, 2075, 1991, 1920, 1669, 1615, 1466, 1365, 1296, 1264, 1147, 1067, 976, 839, 750, 713. **Elemental Analysis**: $C_{12}H_{14}O_4$: calcd. C = 64.85 %, H = 6.35 %; found C = 64.68 %, H = 6.19 %.

(*E*)-Pent-2-en-2,3-dihydroxybenzoate (2^{E2Pent}-H_2)

trans-2-Penten-1-ol (335.35 mg, 3.89 mmol) is converted into the corresponding ester using a modification of the general procedure. 3 equivalents of the alcohol and 6 equivalents of NEt_3 are used and the reaction mixture was refluxed for 3 days. The product is obtained after purification via column chromatography (pentane/ethyl acetate 20:1, R_f = 0.42) as colorless oil (24 %, 70 mg, 0.31 mmol). **^{1}H NMR** (600 MHz, $CDCl_3$): δ = 10.98 (s, 1H, O*H*), 7.40 (dd, *J* = 8.0, 1.5 Hz, 1H, $H_{arom.}$), 7.10 (dd, *J* = 8.0, 1.5 Hz, 1H, $H_{arom.}$), 6.80 (t, *J* = 8.0 Hz, 1H, $H_{arom.}$), 5.88-5.85 (m, 1H, C*H*), 5.67-5.63 (m, 1H,

C*H*), 5.61 (s, 1H, O*H*), 4.80 (dd, *J* = 6.9, 1.2 Hz, 2H, OC*H*$_2$), 2.18-2.15 (m, 2H, C*H*$_2$), 1.01 (t, *J* = 7.5 Hz, 3H, CH$_2$C*H*$_3$) ppm. **^{13}C NMR** (151 MHz, CDCl$_3$): δ = 170.18 (*C*O$_2$CH$_2$), 148.87 (C$_{arom.}$), 144.97 (C$_{arom.}$), 139.03 (CH$_2$*C*H), 122.03 (CH*C*H), 120.53 (C$_{arom.}$), 119.70 (C$_{arom.}$), 119.130 (C$_{arom.}$), 112.59 (C$_{arom.}$), 66.30 (O*C*H$_2$), 25.31 (CH*C*H$_2$), 13.05 (CH$_2$*C*H$_3$) ppm. **MS** (negative ESI-MS, MeOH, acidified): *m/z* (%) = 221.0816 (100, [M-H$^+$], $C_{12}H_{13}O_4^-$, calcd. 221.0813). **IR** (KBr): ṽ (cm^{-1}) = 3932, 3467, 3208, 2961, 2739, 2457, 2248, 1917, 1859, 1671, 1468, 1386, 1302, 1151, 1068, 972, 892, 839, 753, 593, 507. **Elemental Analysis**: $C_{12}H_{14}O_4$: calcd. C = 64.85 %, H = 6.35 %; found C = 64.72 %, H = 6.56 %.

Pent-4-en-2,3-dihydroxybenzoate (2^{4Pent}-H$_2$)

4-Penten-1-ol (335.4 mg, 3.89 mmol) is converted into the corresponding ester using a modification of the general procedure. 3 equivalents of the alcohol and 6 equivalents of NEt$_3$ are used and the reaction mixture was refluxed for 3 days. The product is obtained after purification via column chromatography (pentane/ethyl acetate 20:1, R$_f$ = 0.22) as colorless oil (35 %, 100 mg, 0.45 mmol). **^{1}H NMR** (600 MHz, CDCl$_3$): δ = 10.97 (s, 1H, O*H*), 7.38 (dd, *J* = 8.0, 1.5 Hz, 1H, H$_{arom.}$), 7.09 (dd, *J* = 8.0, 1.5 Hz, 1H, H$_{arom.}$), 6.80 (t, *J* = 8.0 Hz, 1H, H$_{arom.}$), 5.84-5.81 (m, 1H, C*H*), 5.61 (s, 1H, O*H*), 5.13-4.98 (m, 2H, CHC*H*$_2$), 4.38 (t, *J* = 6.6 Hz, 2H, OC*H*$_2$), 2.26-2.20 (m, 2H, C*H*$_2$), 1.94-1.82 (m, 2H, C*H*$_2$) ppm. **^{13}C NMR** (151 MHz, CDCl$_3$): δ = 170.36 (*C*O$_2$CH$_2$), 148.89 (C$_{arom.}$), 145.01 (C$_{arom.}$), 137.13 (CH$_2$*C*H), 120.52 (CH*C*H$_2$), 119.71 (C$_{arom.}$), 119.15 (C$_{arom.}$), 115.65 (C$_{arom.}$), 112.55 (C$_{arom.}$), 64.90 (O*C*H$_2$), 30.01 (*C*H$_2$), 27.66 (*C*H$_2$) ppm. **MS** (negative and positive ESI-MS, MeOH, acidified): *m/z* (%) = 221.0834 (100, [M-H$^+$], $C_{12}H_{13}O_4^-$, calcd. 221.0813), 245.1133 (90, [M+Na$^+$], $C_{12}H_{14}O_4Na^+$, calcd. 245.0886). **IR** (KBr): ṽ (cm^{-1}) = 3473, 3078, 2937, 2465, 2161, 1850, 1668, 1466, 1397, 1301, 1262, 1149, 1067, 993, 913, 840, 749. **Elemental Analysis**: $C_{12}H_{14}O_4$: calcd. C = 64.85 %, H = 6.35 %; found C = 64.23 %, H = 6.20 %.

(*Z*)-Hex-2-en-2,3-dihydroxybenzoate(2^{Z2Hex}-H$_2$)

cis-2-Hexen-1-ol (399.93 mg, 3.89 mmol) is converted into the corresponding ester using a modification of the general procedure. 3 equivalents of the alcohol and 6 equivalents of NEt$_3$ are used and the reaction mixture was refluxed for 3 days. The product is obtained after purification via column chromatography (pentane/ethyl acetate 20:1, R$_f$ = 0.42) as colorless oil (26 %, 80 mg, 0.34 mmol). **^{1}H NMR** (600 MHz, CDCl$_3$): δ = 10.94 (s, 1H, O*H*), 7.38 (dd, *J* = 8.0, 1.5 Hz, 1H, H$_{arom.}$), 7.10 (dd, *J* = 8.0, 1.5 Hz, 1H, H$_{arom.}$), 6.79 (t, *J* = 8.0 Hz, 1H, H$_{arom.}$), 5.78-5.70 (m, 1H, C*H*), 5.68-5.65 (m, 1H, C*H*), 5.63 (s, 1H, O*H*), 4.90 (dd, *J* = 6.9, 1.2 Hz, 2H, OC*H*$_2$), 2.17-2.12 (m, 2H, C*H*$_2$), 1.45-1.42 (m, 2H, C*H*$_2$), 0.93 (t, *J* = 7.4 Hz, 3H, CH$_2$C*H*$_3$) ppm. **^{13}C NMR** (151 MHz, CDCl$_3$): δ = 170.26 (*C*O$_2$CH$_2$), 148.86 (C$_{arom.}$), 144.97 (C$_{arom.}$), 136.32 (CH$_2$*C*H), 122.64 (CHC*H*), 120.64 (C$_{arom.}$), 119.71 (C$_{arom.}$), 119.14 (C$_{arom.}$), 112.55 (C$_{arom.}$), 61.33 (O*C*H$_2$), 29.61 (CH*C*H$_2$), 22.53 (*C*H$_2$), 13.68 (CH$_2$*C*H$_3$) ppm. **MS** (negative ESI-MS, MeOH, acidified): *m/z* (%) = 235.0969 (40, [M-H$^+$], $C_{13}H_{15}O_4^-$, calcd. 235.0970). **IR** (KBr): ṽ (cm^{-1}) = 3472, 3180, 3026, 2960, 2932, 2870, 2323, 2211, 2159, 2055, 1996, 1951, 1669, 1614, 1466, 1413, 1367, 1298, 1264, 1146, 1067, 974, 897, 839, 751, 703. **Elemental Analysis:** $C_{13}H_{16}O_4$: calcd. C = 66.09 %, H = 6.83 %; found C = 65.72 %, H = 6.88 %.

(*E*)-Hex-2-en-2,3-dihydroxybenzoate(2^{E2Hex}-H$_2$)

trans-2-Hexen-1-ol (389.93 mg, 3.89 mmol) is converted into the corresponding ester using a modification of the general procedure. 3 equivalents of the alcohol and 6 equivalents of NEt$_3$ are used and the reaction mixture was refluxed for 3 days. The product is obtained after purification via column chromatography (pentane/ethyl acetate 20:1, R$_f$ = 0.17) as colorless oil (27 %, 84 mg,

0.35 mmol). **^{1}H NMR** (600 MHz, $CDCl_3$): δ = 10.95 (s, 1H, O*H*), 7.39 (dd, *J* = 8.0, 1.5 Hz, 1H, $H_{arom.}$), 7.10 (dd, *J* = 8.0, 1.5 Hz, 1H, $H_{arom.}$), 6.79 (t, *J* = 8.0 Hz, 1H, $H_{arom.}$), 5.92-5.84 (m, 1H, C*H*), 5.71-5.63 (m, 1H, C*H*), 5.64 (s, 1H, O*H*), 4.79 (d, *J* = 6.6 Hz, 2H, OCH_2), 2.09-2.05 (m, 2H, CH_2), 1.46-1.43 (m, 2H, CH_2), 0.92 (t, *J* = 7.4 Hz, 3H, CH_2CH_3) ppm. **^{13}C NMR** (151 MHz, $CDCl_3$): δ = 170.17 (CO_2CH_2), 148.87 ($C_{arom.}$), 144.97 ($C_{arom.}$), 137.47 (CH_2CH), 123.13 (CH*C*H), 120.65 ($C_{arom.}$), 119.69 ($C_{arom.}$), 119.13 ($C_{arom.}$), 112.60 ($C_{arom.}$), 66.30 (OCH_2), 34.33 (CHCH_2), 21.99 (CH_2), 13.65 (CH_2CH_3) ppm. **MS** (negative ESI-MS, MeOH, acidified): *m/z* (%) = 235.0951 (100, [M-H$^+$], $C_{13}H_{15}O_4^-$, calcd. 235.0970). **IR** (KBr): ṽ (cm^{-1}) = 3470, 3179, 2959, 2931, 2872, 2323, 2159, 2070, 1668, 1616, 1466, 1386, 1301, 1263, 1145, 1067, 969, 903, 839, 750, 712. **Elemental Analysis:** $C_{13}H_{16}O_4$: calcd. C = 66.09 %, H = 6.83 %; found C = 65.49 %, H = 6.90 %.

(*Z*)-Hex-3-en-2,3-dihydroxybenzoate (2^{Z3Hex}-H_2)

cis-3-Hexen-1-ol (389.93 mg, 3.89 mmol) is converted into the corresponding ester using a modification of the general procedure. 3 equivalents of the alcohol and 6 equivalents of NEt_3 are used and the reaction mixture was refluxed for 3 days. The product is obtained after purification via column chromatography (pentane/ethyl acetate 20:1, R_f = 0.32) as colorless oil (35 %, 106 mg, 0.45 mmol). **^{1}H NMR:** (600 MHz, $CDCl_3$): δ = 10.98 (s, 1H, O*H*), 7.38 (dd, *J* = 8.0, 1.5 Hz, 1H, $H_{arom.}$), 7.11 (dd, *J* = 8.0, 1.5 Hz, 1H, $H_{arom.}$), 6.80 (t, *J* = 8.0 Hz, 1H, $H_{arom.}$), 5.65 (s, 1H, O*H*), 5.58-5.49 (m, 1H, C*H*), 5.42-5.38 (m, 1H, C*H*), 4.35 (t, *J* = 6.6 Hz, 2H, OCH_2), 2.24-2.20 (m, 2H, CH_2), 1.86-1.84 (m, 2H, CH_2), 1.62 (t, *J* = 7.4 Hz, 3H, CH_2CH_3) ppm. **^{13}C NMR** (151 MHz, $CDCl_3$): δ = 170.40 (CO_2CH_2), 148.89 ($C_{arom.}$), 145.01 ($C_{arom.}$), 128.73 (CH_2CH), 125.33 (CH*C*H), 120.50 ($C_{arom.}$), 119.68 ($C_{arom.}$), 119.14 ($C_{arom.}$), 112.59 ($C_{arom.}$), 65.01 (OCH_2), 28.28 (CH_2), 23.12 (CHCH_2), 12.77 (CH_2CH_3) ppm. **MS** (negative ESI-MS, MeOH): *m/z* (%) = 235.1008 (30, [M-H$^+$], $C_{13}H_{15}O_4^-$, calcd. 235.0970). **IR** (KBr): ṽ (cm^{-1}) = 3466, 3176, 3036, 2963, 2930, 2663, 2327, 2191, 2167, 2107, 1995, 1952, 1797, 1730, 1671, 1607, 1535, 1468, 1394, 1306, 1266, 1153, 1068, 997, 969, 900, 842, 789, 752, 703. **Elemental Analysis:** $C_{13}H_{16}O_4$ · 5 MeOH: calcd. C = 54.53 %, H = 9.15 %; found C = 54.63 %, H = 8.69 %.

(*E*)-Hex-3-en-2,3-dihydroxybenzoate (2^{E3Hex}-H_2)

trans-3-Hexen-1-ol (389.93 mg, 3.89 mmol) is converted into the corresponding ester using a modification of the general procedure. 3 equivalents of the alcohol and 6 equivalents of NEt_3 are used and the reaction mixture was refluxed for 3 days. The product is obtained after purification via column chromatography (pentane/ethyl acetate 20:1, R_f = 0.27) as colorless oil (32 %, 98 mg, 0.41 mmol). **^{1}H NMR** (600 MHz, $CDCl_3$): δ = 10.93 (s, 1H, O*H*), 7.37 (dd, *J* = 8.0, 1.5 Hz, 1H, $H_{arom.}$), 7.10 (dd, *J* = 8.0, 1.5 Hz, 1H, $H_{arom.}$), 6.80 (t, *J* = 8.0 Hz, 1H, $H_{arom.}$), 5.67-5.58 (m, 1H, C*H*), 5.62 (s, 1H, O*H*), 5.45-5.42 (m, 1H, C*H*), 4.35 (t, *J* = 6.8 Hz, 2H, OCH_2), 2.49-2.45 (m, 2H, CH_2), 2.12-1.99 (m, 2H, CH_2), 0.97 (t, *J* = 7.4 Hz, 3H, CH_2CH_3) ppm. **^{13}C NMR** (151 MHz, $CDCl_3$): δ = 170.26 (CO_2CH_2), 148.82 ($C_{arom.}$), 144.97 ($C_{arom.}$), 135.71 (CH_2CH), 123.53 (CH*C*H), 120.58 ($C_{arom.}$), 119.66 ($C_{arom.}$), 119.14 ($C_{arom.}$), 112.62 ($C_{arom.}$), 65.14 (OCH_2), 31.85 (CH_2), 25.65 (CHCH_2), 13.70 (CH_2CH_3) ppm. **MS** (positive ESI-MS, MeOH, acidified): *m/z* (%) = 243.1205 (10, [M+Li$^+$], $C_{13}H_{16}O_4Li^+$, calcd. 243.1209). **IR** (KBr): ṽ (cm^{-1}) = 3470, 3141, 2963, 2733, 2328, 2104, 1992, 1918, 1669, 1614, 1466, 1394, 1303, 1264, 1149, 1067, 996, 968, 900, 841, 750, 708. **Elemental Analysis:** $C_{13}H_{16}O_4$: calcd. C = 66.09 %, H = 6.83 %; found C = 65.05 %, H = 6.86 %.

(*Z*)-Hex-4-en-2,3-dihydroxybenzoate (2^{Z4Hex}-H_2)

cis-4-Hexen-1-ol (389.93 mg, 3.89 mmol) is converted into the corresponding ester using a modification of the general procedure. 3 equivalents of the alcohol and 6 equivalents of NEt_3 are used and the reaction mixture was refluxed for 3 days. The product is obtained after purification via column chromatography (pentane/ethyl acetate 20:1, R_f = 0.19) as colorless oil (26 %, 78 mg, 0.33 mmol). **^{1}H NMR** (600 MHz, $CDCl_3$): δ = 10.96 (s, 1H, O*H*), 7.37 (dd, *J* = 8.0, 1.5 Hz, 1H, $H_{arom.}$), 7.10 (dd, *J* = 8.0, 1.5 Hz, 1H, $H_{arom.}$), 6.78 (t, *J* = 8.0 Hz, 1H, $H_{arom.}$), 5.60 (s, 1H, O*H*), 5.55-5.39 (m, 2H, 2×C*H*), 4.38 (t, *J* = 6.6 Hz, 2H, OCH_2), 2.19-2.16 (m, 2H, CH_2), 1.85-1.82 (m, 2H, CH_2), 1.61-1.58 (m, 3H, CHCH_3) ppm. **^{13}C NMR** (151 MHz, $CDCl_3$): δ = 170.05 (CO_2CH_2), 148.87 ($C_{arom.}$), 145.02 ($C_{arom.}$), 129.61 (CH_2CH), 126.19 (CHCH), 120.52 ($C_{arom.}$), 119.65 ($C_{arom.}$), 119.11 ($C_{arom.}$), 112.62 ($C_{arom.}$), 65.03 (OCH_2), 28.86 (CH_2CH), 28.29 (CH_2), 17.88 (CHCH_3) ppm. **MS** (negative ESI-MS, MeOH, acidified): *m/z* (%) = 235.0939 (100, [M-H^+], $C_{13}H_{15}O_4^-$, calcd. 235.0970). **IR** (KBr): ṽ (cm^{-1}) = 3881, 3421, 3014, 2943, 2849, 2670, 2320, 2094, 1994, 1669, 1466, 1396, 1302, 1264, 1150, 1067, 1003, 902, 840, 752, 698. **Elemental Analysis:** $C_{13}H_{16}O_4$: calcd. C = 66.09 %, H = 6.83 %; found C = 65.59 %, H = 6.75 %.

(*E*)-Hex-4-en-2,3-dihydroxybenzoate (2^{E4Hex}-H_2)

Trans-4-Hexen-1-ol (389.93 mg, 3.89 mmol) is converted into the corresponding ester using a modification of the general procedure. 3 equivalents of the alcohol and 6 equivalents of NEt_3 are used and the reaction mixture was refluxed for 3 days. The product is obtained after purification via column chromatography (pentane/ethyl acetate 20:1, R_f = 0.38) as colorless oil (32 %, 96 mg, 0.41 mmol). **^{1}H NMR** (600 MHz, $CDCl_3$) : δ = 10.96 (s, 1H, O*H*), 7.36 (dd, *J* = 8.0, 1.5 Hz, 1H, $H_{arom.}$), 7.09 (dd, *J* = 8.0, 1.5 Hz, 1H, $H_{arom.}$), 6.78 (t, *J* = 8.0 Hz, 1H, $H_{arom.}$), 5.60 (s, 1H, O*H*), 5.54-5.37 (m, 2H, 2×C*H*), 4.33 (t, *J* = 6.6 Hz, 2H, OCH_2), 2.20-2.17 (m, 2H, CH_2), 1.87-1.84 (m, 2H, CH_2), 1.62 (t, *J* = 6.9 Hz, 3H, CHCH_3) ppm. **^{13}C NMR** (151 MHz, $CDCl_3$): δ = 170.16 (CO_2CH_2), 148.88 ($C_{arom.}$), 144.99 ($C_{arom.}$), 137.44 (CH_2CH), 123.14 (CHCH), 120.64 ($C_{arom.}$), 119.68 ($C_{arom.}$), 119.11 ($C_{arom.}$), 112.62 ($C_{arom.}$), 66.27 (OCH_2), 34.31 (CH_2CH), 21.98 (CH_2), 13.62 (CHCH_3) ppm. **MS** (negative and positive ESI-MS, MeOH, acidified): *m/z* (%) = 235.0977 (90, [M-H^+], $C_{13}H_{15}O_4^-$, calcd. 235.0970), 259.1154 (80, [M+Na^+], $C_{13}H_{16}O_4Na^+$, calcd. 259.0947). **IR** (KBr): ṽ (cm^{-1}) = 3883, 3466, 3149, 2933, 2854, 2660, 2322, 2088, 1996, 1669, 1466, 1395, 1303, 1265, 1151, 1068, 966, 904, 841, 750, 710. **Elemental Analysis:** $C_{13}H_{16}O_4$ · 1/3 MeOH: calcd. C = 64.85 %, H = 7.08 %; found C = 64.93 %, H = 7.05 %.

Hex-5-en-2,3-dihydroxybenzoate (2^{5Hex}-H_2)

5-Hexen-1-ol (389.93 mg, 3.89 mmol) is converted into the corresponding ester using a modification of the general procedure. 3 equivalents of the alcohol and 6 equivalents of NEt_3 are used and the reaction mixture was refluxed for 3 days. The product is obtained after purification via column chromatography (pentane/ethyl acetate 20:1, R_f = 0.21) as colorless oil (29 %, 90 mg, 0.38 mmol). **^{1}H NMR** (600 MHz, $CDCl_3$): δ = 10.98 (s, 1H, O*H*), 7.37 (dd, *J* = 8.0, 1.4 Hz, 1H, $H_{arom.}$), 7.11 (dd, *J* = 8.0, 1.4 Hz, 1H, $H_{arom.}$), 6.80 (t, *J* = 8.0 Hz, 1H, $H_{arom.}$), 5.83-5.80 (m, 1H, C*H*), 5.64 (s, 1H, O*H*), 5.08-4.93 (m, 2H, CH_2), 4.36 (t, *J* = 6.6 Hz, 2H, OCH_2), 2.17-2.05 (m, 2H, CH_2), 1.82-1.79 (m, 2H, CH_2), 1.57-1.45 (m, 2H, CH_2) ppm. **^{13}C NMR** (151 MHz, $CDCl_3$): δ = 170.40 (CO_2CH_2), 148.88 ($C_{arom.}$), 145.00 ($C_{arom.}$), 138.15 (CH_2CH), 120.52 ($C_{arom.}$), 119.68 ($C_{arom.}$), 119.14 ($C_{arom.}$), 115.06 (CHCH_2), 112.58 ($C_{arom.}$), 65.47 (OCH_2), 33.24 (CH_2), 28.88 (CH_2), 24.97 (CH_2) ppm. **MS** (negative ESI-MS, MeOH, acidified): *m/z* (%) = 235.0950 (100, [M-H^+], $C_{13}H_{15}O_4^-$, calcd. 235.0970). **IR** (KBr): ṽ (cm^{-1}) = 3470, 3076, 2935, 2861, 2659, 2322, 2102, 1994, 1917, 1830, 1669, 1466, 1398, 1303, 1264, 1150, 1067, 994, 911, 842, 751, 713. **Elemental Analysis**: $C_{13}H_{16}O_4$: calcd. C = 66.09 %, H = 6.83 %; found C = 65.65 %, H = 6.89 %.

Hept-6-en-2,3-dihydroxybenzoate (2^{6Hept}-H_2)

6-Hepten-1-ol (444.54 mg, 3.89 mmol) is converted into the corresponding ester using a modification of the general procedure. 3 equivalents of the alcohol and 6 equivalents of NEt_3 are used and the reaction mixture was refluxed for 3 days. The product is obtained after purification via column chromatography (pentane/ethyl acetate 20:1, R_f = 0.20) as colorless oil (30 %, 98 mg, 0.39 mmol). **^{1}H NMR** (400 MHz, $CDCl_3$): δ = 10.98 (s, 1H, O*H*), 7.39 (dd, *J* = 8.0, 1.5 Hz, 1H, $H_{arom.}$), 7.09 (dd, *J* = 8.0, 1.5 Hz, 1H, $H_{arom.}$), 6.79 (t, *J* = 8.0 Hz, 1H, $H_{arom.}$), 5.83-5.75 (m, 1H, C*H*), 5.61 (s, 1H, O*H*), 5.01-4.95 (m, 2H, CH_2), 4.38 (t, *J* = 6.6 Hz, 2H, OCH_2), 2.08-2.05 (m, 2H, CH_2), 1.80-1.75 (m, 2H, CH_2), 1.43-1.36 (m, 4H, 2×CH_2) ppm. **^{13}C NMR** (101 MHz, $CDCl_3$): δ = 170.39 (CO_2CH_2), 148.87 ($C_{arom.}$), 145.00 ($C_{arom.}$), 138.55 (CH_2CH), 120.49 ($C_{arom.}$), 119.64 ($C_{arom.}$), 119.10 ($C_{arom.}$), 114.60 ($CHCH_2$), 112.60 ($C_{arom.}$), 65.57 (OCH_2), 33.53 (CH_2), 29.35 (CH_2), 28.35 (CH_2), 25.36 (CH_2) ppm. **MS** (negative ESI-MS, MeOH, acidified): *m/z* (%) = 249.1132 (100, [M-H$^+$], $C_{14}H_{17}O_4^-$, calcd. 249.1111). **IR** (KBr): ṽ (cm^{-1}) = 3442, 3076, 2931, 2859, 2400, 1826, 1673, 1641, 1464, 1383, 1305, 1268, 1206, 932, 883, 709, 638, 593, 554, 504. **Elemental Analysis**: $C_{14}H_{18}O_4$ · 2 MeOH: calcd. C = 61.13 %, H = 8.34 %; found C = 61.26 %, H = 9.10 %.

Oct-7-en-2,3-dihydroxybenzoate (2^{7Oct}-H_2)

7-Octen-1-ol (499.15 mg, 3.89 mmol) is converted into the corresponding ester using a modification of the general procedure. 3 equivalents of the alcohol and 6 equivalents of NEt_3 are used and the reaction mixture was refluxed for 3 days. The product is obtained after purification via column chromatography (pentane/ethyl acetate 20:1, R_f = 0.23) as colourless oil (29 %, 100 mg, 0.38 mmol). **^{1}H NMR** (400 MHz, $CDCl_3$): δ = 10.98 (s, 1H, O*H*), 7.39 (dd, *J* = 8.0, 1.5 Hz, 1H, $H_{arom.}$), 7.09 (dd, *J* = 8.0, 1.5 Hz, 1H, $H_{arom.}$), 6.79 (t, *J* = 8.0 Hz, 1H, $H_{arom.}$), 5.81-5.75 (m, 1H, C*H*), 5.61 (s, 1H, O*H*), 5.01-4.96 (m, 2H, CH_2), 4.38 (t, *J* = 6.6 Hz, 2H, OCH_2), 2.08-2.05 (m, 2H, CH_2), 1.80-1.75 (m, 2H, CH_2), 1.43-1.29 (m, 6H, 3×CH_2) ppm. **^{13}C NMR** (101 MHz, $CDCl_3$): δ = 170.39 (CO_2CH_2), 148.87 ($C_{arom.}$), 145.00 ($C_{arom.}$), 138.84 (CH_2CH), 120.49 ($C_{arom.}$), 119.63 ($C_{arom.}$), 119.10 ($C_{arom.}$), 114.37 ($CHCH_2$), 112.62 ($C_{arom.}$), 65.63 (OCH_2), 33.62 (CH_2), 29.44 (CH_2), 28.64 (CH_2), 25.77 (CH_2), 25.59 (CH_2) ppm. **MS** (negative and positive ESI-MS, MeOH, acidified): *m/z* (%) = 263.1289 (100, [M-H$^+$], $C_{15}H_{19}O_4^-$, calcd. 263.1268), 287.1257 (60, [M+Na$^+$], $C_{15}H_{20}O_4Na^+$, calcd. 287.1260). **IR** (KBr): ṽ (cm^{-1}) = 3552, 3446, 3076, 2925, 2857, 2403, 1982, 1825, 1673, 1641, 1543, 1463, 1381, 1303, 1206, 905, 825, 708, 638, 595, 553, 512. **Elemental Analysis**: $C_{15}H_{20}O_4$ · 3/2 MeOH: calcd. C = 63.44 %, H = 8.39 %; found C = 63.20 %, H = 9.60 %.

Non-8-en-2,3-dihydroxybenzoate (2^{8Non}-H_2)

8-Nonen-1-ol (553.75 mg, 3.89 mmol) is converted into the corresponding ester using a modification of the general procedure. 3 equivalents of the alcohol and 6 equivalents of NEt_3 are used and the reaction mixture was refluxed for 3 days. The product is obtained after purification via column chromatography (pentane/ethyl acetate 20:1, R_f = 0.18) as colourless oil (30 %, 110 mg, 0.39 mmol). **^{1}H NMR** (600 MHz, $CDCl_3$): δ = 11.00 (s, 1H, O*H*), 7.39 (dd, *J* = 8.0, 1.4 Hz, 1H, $H_{arom.}$), 7.11 (dd, *J* = 8.0, 1.4 Hz, 1H, $H_{arom.}$), 6.80 (t, *J* = 8.0 Hz, 1H, $H_{arom.}$), 5.82-5.79 (m, 1H, C*H*), 5.61 (s, 1H, O*H*), 5.01-4.96 (m, 2H, CH_2), 4.36 (t, *J* = 6.6 Hz, 2H, OCH_2), 2.10-2.06 (m, 2H, CH_2), 1.81-1.78 (m, 2H, CH_2), 1.45-1.28 (m, 8H, 4×CH_2) ppm. **^{13}C NMR** (151 MHz, $CDCl_3$): δ = 170.42 (CO_2CH_2), 148.87 ($C_{arom.}$), 145.00 ($C_{arom.}$), 139.04 (CH_2CH), 120.52 ($C_{arom.}$), 119.66 ($C_{arom.}$), 119.12 ($C_{arom.}$), 114.27 ($CHCH_2$), 112.63 ($C_{arom.}$), 65.82 (OCH_2), 33.73 (CH_2), 30.95 (CH_2), 29.05 (CH_2), 28.95 (CH_2), 28.49 (CH_2), 25.88 (CH_2) ppm. **MS** (negative and positive ESI-MS, MeOH, acidified): *m/z* (%) = 277.1454 (100, [M-H$^+$], $C_{16}H_{21}O_4^-$, calcd. 277.1424), 301.1402 (100, [M+Na$^+$], $C_{16}H_{22}O_4Na^+$, calcd. 301.1416). **IR** (KBr): ṽ (cm^{-1}) = 3469, 3144, 3076, 2928, 2856, 2458, 1912, 1852, 1672, 1469, 1397, 1307, 1267, 1154, 1068, 992, 910, 843, 753, 719, 639,

592, 527, 492. **Elemental Analysis**: $C_{16}H_{22}O_4$: calcd. C = 69.04 %, H = 7.97 %; found C = 68.61 %, H = 8.13 %.

Dec-9-en-2,3-dihydroxybenzoate (2^{9Dec}-H_2)

9-Decen-1-ol (608.36 mg, 3.89 mmol) is converted into the corresponding ester using a modification of the general procedure. 3 equivalents of the alcohol and 6 equivalents of NEt_3 are used and the reaction mixture was refluxed for 3 days. The product is obtained after purification via column chromatography (pentane/ethyl acetate 20:1, R_f = 0.25) as colourless oil (22 %, 82 mg, 0.28 mmol). **^{1}H NMR** (600 MHz, $CDCl_3$): δ = 10.98 (s, 1H, O*H*), 7.40 (dd, *J* = 8.0, 1.4 Hz, 1H, $H_{arom.}$), 7.11 (dd, *J* = 8.0, 1.4 Hz, 1H, $H_{arom.}$), 6.80 (t, *J* = 8.0 Hz, 1H, $H_{arom.}$), 5.82-5.79 (m, 1H, C*H*), 5.62 (s, 1H, O*H*), 5.03-4.93 (m, 2H, CH_2), 4.36 (t, *J* = 6.6 Hz, 2H, OCH_2), 2.05-2.02 (m, 2H, CH_2), 1.82-1.79 (m, 2H, CH_2), 1.43-1.23 (m, 10H, 5×CH_2) ppm. **^{13}C NMR** (151 MHz, $CDCl_3$): δ = 170.42 (CO_2CH_2), 148.87 ($C_{arom.}$), 145.00 ($C_{arom.}$), 139.13 (CH_2CH), 120.52 ($C_{arom.}$), 119.65 ($C_{arom.}$), 119.11 ($C_{arom.}$), 114.19 ($CHCH_2$), 112.63 ($C_{arom.}$), 65.70 (OCH_2), 33.77 (CH_2), 29.48 (CH_2), 29.32 (CH_2), 29.00 (CH_2), 28.86 (CH_2), 28.77 (CH_2), 25.73 (CH_2) ppm. **MS** (negative and positive ESI-MS, MeOH, acidified): *m/z* (%) = 291.1643 (100, [M-H$^+$], $C_{17}H_{23}O_4^-$, calcd. 291.1554), 315.1560 (50, [M+Na$^+$] $C_{17}H_{24}O_4Na^+$, calcd. 315.1572). **IR** (KBr): $\tilde{\nu}$ (cm^{-1}) = 3888, 3785, 3661, 3466, 3210, 3077, 2926, 2855, 2740, 2457, 2254, 1911, 1851, 1671, 1540, 1468, 1394, 1306, 1267, 1237, 1153, 1068, 993, 909, 843, 753, 718, 639, 592, 495. **Elemental Analysis**: $C_{17}H_{24}O_4$: calcd. C = 69.84 %, H = 8.27 %; found C = 69.28 %, H = 8.37 %.

Undec-10-en-2,3-dihydroxybenzoate ($2^{10Undec}$-H_2)

10-Undecen-1-ol (662.95 mg, 3.89 mmol) is converted into the corresponding ester using a modification of the general procedure. 3 equivalents of the alcohol and 6 equivalents of NEt_3 are used and the reaction mixture was refluxed for 3 days. The product is obtained after purification via column chromatography (pentane/ethyl acetate 20:1, R_f = 0.24) as colourless oil (19 %, 78 mg, 0.25 mmol). **^{1}H NMR** (600 MHz, $CDCl_3$): δ = 11.00 (s, 1H, O*H*), 7.40 (dd, *J* = 8.0, 1.4 Hz, 1H, $H_{arom.}$), 7.11 (dd, *J* = 8.0, 1.4 Hz, 1H, $H_{arom.}$), 6.80 (t, *J* = 8.0 Hz, 1H, $H_{arom.}$), 5.83-5.80 (m, 1H, C*H*), 5.62 (s, 1H, O*H*), 5.02-4.95 (m, 2H, CH_2), 4.38 (t, *J* = 6.6 Hz, 2H, OCH_2), 2.12-2.05 (m, 2H, CH_2), 1.81-1.78 (m, 2H, CH_2), 1.51-1.22 (m, 12H, 6×CH_2) ppm. **^{13}C NMR** (151 MHz, $CDCl_3$): δ = 170.43 (CO_2CH_2), 148.87 ($C_{arom.}$), 145.00 ($C_{arom.}$), 139.19 (CH_2CH), 120.52 ($C_{arom.}$), 119.65 ($C_{arom.}$), 119.11 ($C_{arom.}$), 114.14 ($CHCH_2$), 112.64 ($C_{arom.}$), 65.72 (OCH_2), 33.79 (CH_2), 29.49 (CH_2), 29.43 (CH_2), 29.19 (CH_2), 29.08 (CH_2), 28.90 (CH_2), 28.51 (CH_2), 25.74 (CH_2) ppm. **MS** (negative and positive ESI-MS, MeOH, acidified): *m/z* (%) = 305.1763 (100, [M-H$^+$], $C_{18}H_{25}O_4^-$, calcd. 305.1737), 329.1715 (100, [M+Na$^+$] $C_{18}H_{26}O_4Na^+$, calcd. 329.1729). **IR** (KBr): $\tilde{\nu}$ (cm^{-1}) = 3471, 3076, 2927, 2855, 2459, 1911, 1672, 1469, 1397, 1307, 1267, 1237, 1154, 1068, 994, 909, 843, 753, 718, 639, 593. **Elemental Analysis**: $C_{18}H_{26}O_4$: calcd. C = 70.56 %, H = 8.55 %; found C = 70.22 %, H = 8.54 %.

Complexes Li[$Li_3(2_3Ti)_2$]

Li[$Li_3(2^{All}{}_3Ti)_2$]:

Ligand **2^{All}**-H_2 (20 mg, 0.10 mmol) is converted into the corresponding complex in methanol (25 mL). The product is obtained after removal of the solvent under reduced pressure as red solid (quantitative). **^{1}H NMR** (600 MHz, MeOH-d_4): Dimer (major component): δ = 7.14 (dd, *J* = 7.1, 2.6 Hz, 1H, $H_{arom.}$), 6.60-6.56 (m, 2H, $H_{arom.}$), 5.75-5.67 (m, 1H, C*H*), 5.16 (dd, *J* = 17.3, 1.5 Hz, 1H, $CHCH_2$), 5.09

(dd, *J* = 10.5, 1.5 Hz, 1H, CHC*H*$_2$), 4.12-4.06 (m, 1H, OC*H*$_2$), 3.59-3.53 (m, 1H, OC*H*$_2$) ppm. Monomer (minor component): δ = 7.38 (dd, *J* = 8.0, 1.6 Hz, 1H, H$_{arom.}$), 7.00 (dd, *J* = 8.0, 1.6 Hz, 1H, H$_{arom.}$), 6.76 (t, *J* = 8.0 Hz, 1H, H$_{arom.}$), 6.11-6.03 (m, 1H, C*H*), 5.42 (dd, *J* = 17.2, 1.4 Hz, 1H, CHC*H*$_2$), 5.30 (dd, *J* = 10.5, 1.4 Hz, 1H, CHC*H*$_2$) ppm. Signals not listed are overlapping with the solvent peak. **^{1}H NMR** (400 MHz, DMSO-*d*$_6$): Dimer (minor component): δ = 6.96 (dd, *J* = 7.9, 1.6 Hz, 1H, H$_{arom.}$), 6.46 (t, *J* = 7.9 Hz, 1H, H$_{arom.}$), 6.43 (dd, *J* = 7.9, 1.6 Hz, 1H, H$_{arom.}$), 5.61-5.57 (m, 1H, C*H*), 5.13-5.02 (m, 2H, C*H*$_2$), 4.02-3.98 (m, 1H, OC*H*$_2$), 3.52-3.45 (m, 1H, OC*H*$_2$) ppm. Monomer (minor component): δ = 6.77 (dd, 1H, *J* = 7.9, 1.6 Hz, 1H, H$_{arom.}$), 6.24 (t, *J* = 7.9 Hz, 1H, H$_{arom.}$), 6.11 (dd, *J* = 7.9, 1.6 Hz, 1H, H$_{arom.}$), 5.97-5.83 (m, 1H, C*H*), 5.48-5.44 (m, 2H, C*H*$_2$) 4.57 (d, *J* = 2.4 Hz, 2H, OC*H*$_2$) ppm. **MS** (negative ESI-MS, MeOH): *m/z* (%) = 1269.1896 (100, [M$_D$-Li$^+$], $C_{60}H_{48}O_{24}Li_3Ti_2^-$, calcd. 1269.1980). **IR** (KBr): $\tilde{\nu}$ (cm^{-1}) = 3895, 3361, 3073, 2940, 2682, 2263, 2199, 2157, 2040, 1987, 1897, 1677, 1593, 1560, 1442, 1368, 1296, 1251, 1154, 1065, 993, 932, 853, 801, 742, 682. **Elemental analysis** $C_{60}H_{48}O_{24}Li_4Ti_2 \cdot 4\,H_2O$: calcd. C = 53.44 %, H = 4.19 %; found C = 53.43 %, H = 4.12 %.

Li[Li$_3$(2^{3Bu}$_3$Ti)$_2$]

2^{3Bu}-H$_2$ (30 mg, 0.14 mmol) is converted into the corresponding complex in methanol (30 mL). The product is obtained after removal of the solvent under reduced pressure as red solid (quantitative). **^{1}H NMR** (600 MHz, MeOH-*d*$_4$): Dimer (major component): δ = 7.11 (dd, *J* = 6.5, 3.2 Hz, 1H, H$_{arom.}$), 6.61-6.53 (m, 2H, H$_{arom.}$), 5.71-5.67 (m, 1H, C*H*), 5.04-4.94 (m, 2H, C*H*$_2$), 3.66-3.62 (m, 1H, OC*H*$_2$), 3.12-3.08 (m, 1H, OC*H*$_2$), 2.22-2.10 (m, 2H, C*H*$_2$) ppm. Monomer (minor component): δ = 7.34 (dd, *J* = 7.9, 1.5 Hz, 1H, H$_{arom.}$), 7.01 (dd, *J* = 7.9, 1.5 Hz, 1H, H$_{arom.}$), 6.75 (t, *J* = 7.9 Hz, 1H, H$_{arom.}$), 5.91-5.82 (m, 1H, C*H*), 5.17-5.10 (m, 2H, CHC*H*$_2$), 4.20 (t, *J* = 6.5 Hz, 2H, OC*H*$_2$), 2.57-2.54 (m, 2H, C*H*$_2$) ppm. **^{1}H NMR** (400 MHz, DMSO-*d*$_6$): Dimer (major component): δ = 6.94 (dd, *J* = 7.9, 1.5 Hz, 1H, H$_{arom.}$), 6.48 (t, *J* = 7.9 Hz, 1H, H$_{arom.}$), 6.38 (dd, *J* = 7.9, 1.5 Hz, 1H, H$_{arom.}$), 5.63-5.60 (m, 1H, C*H*), 5.13-4.99 (m, 2H, C*H*$_2$), 3.55-3.51 (m, 1H, OC*H*$_2$), 2.96-2.92 (m, 1H, OC*H*$_2$), 2.16-2.00 (m, 2H, C*H*$_2$) ppm. Monomer (minor component): δ = 6.73 (dd, *J* = 7.9, 1.5 Hz, 1H, H$_{arom.}$), 6.22 (t, *J* = 7.9 Hz, 1H, H$_{arom.}$), 6.09 (dd, *J* = 7.9, 1.5 Hz, 1H, H$_{arom.}$), 5.86-5.81 (m, 1H, C*H*), 5.10-5.06 (m, 2H, C*H*$_2$), 4.07 (t, *J* = 6.7 Hz, 2H, OC*H*$_2$), 2.37-2.34 (m, 2H, C*H*$_2$) ppm. **MS** (negative and positive ESI-MS, MeOH): *m/z* (%) = 1353.2916 (100, [M$_D$-Li$^+$], $C_{66}H_{60}O_{24}Li_3Ti_2^-$, calcd. 1353.2914), 1367.3209 (10, [M$_D$+Li$^+$], $C_{66}H_{60}O_{24}Li_5Ti_2^+$, calcd. 1367.3233). **IR** (KBr): $\tilde{\nu}$ (cm^{-1}) = 3863, 3359, 3072, 2961, 2680, 2314, 2196, 2127, 2002, 1898, 1837, 1738, 1674, 1594, 1442, 1387, 1344, 1295, 1251, 1210, 1154, 1064, 992, 913, 854, 804, 741, 680. **Elemental analysis:** $C_{66}H_{60}O_{24}Li_4Ti_2 \cdot 4\,H_2O$: calcd. C = 55.33 %, H = 4.78 %; found C = 55.04 %, H = 4.75 %.

Li[Li$_3$(2^{Z2Pent}$_3$Ti)$_2$]

2^{Z2Pent}-H$_2$ (20 mg, 0.09 mmol) is converted into the corresponding complex in methanol (25 mL). The product is obtained after removal of the solvent under reduced pressure as red solid (quantitative). **^{1}H NMR** (600 MHz, MeOH-*d*$_4$): Only dimer: δ = 7.08 (dd, *J* = 7.8, 1.6 Hz, 1H, H$_{arom.}$), 6.62-6.49 (m, 2H, H$_{arom.}$), 5.46-5.37 (m, 1H, C*H*), 5.29-5.15 (m, 1H, C*H*), 4.21-4.18 (m, 1H, OC*H*$_2$), 3.58-3.55 (m, 1H, OC*H*$_2$), 2.02-1.97 (m, 2H, C*H*$_2$), 0.76 (t, *J* = 7.5 Hz, 3H, CH$_2$C*H*$_3$) ppm. **^{1}H NMR** (600 MHz, DMSO-*d*$_6$): Dimer (major component): δ = 6.93 (dd, *J* = 7.8, 1.6 Hz, 1H, H$_{arom.}$), 6.47 (t, *J* = 7.8 Hz, 1H, H$_{arom.}$), 6.41 (dd, *J* = 7.8, 1.6 Hz, 1H, H$_{arom.}$), 5.41-5.39 (m, 1H, C*H*), 5.22-5.11 (m, 1H, C*H*), 4.05-4.02 (m, 1H, OC*H*$_2$), 3.48-3.45 (m, 1H, OC*H*$_2$), 1.91-1.77 (m, 2H, C*H*$_2$), 0.74 (t, *J* = 7.5 Hz, 3H, CH$_2$C*H*$_3$) ppm. Monomer

(minor component): δ = 6.72 (dd, *J* = 7.8, 1.6 Hz, 1H, $H_{arom.}$), 6.22 (t, *J* = 7.8 Hz, 1H, $H_{arom.}$), 6.09 (dd, *J* = 7.8, 1.6 Hz, 1H, $H_{arom.}$), 5.53-5.50 (m, 2H, C*H*C*H*), 4.62 (t, *J* = 6.7 Hz, 2H, OCH_2), 2.14-2.02 (m, 2H, CH_2), 0.91 (t, *J* = 7.5 Hz, 3H, CH_2CH_3) ppm. **MS** (negative and positive ESI-MS, MeOH): *m/z* (%) = 1437.4005 (100, [M_D-Li^+], $C_{72}H_{72}O_{24}Li_3Ti_2^-$, calcd. 1437.3853), 1451.4214 (10, [M_D+Li^+], $C_{72}H_{72}O_{24}Li_5Ti_2^+$, calcd. 1451.4173). **IR** (KBr): ṽ (cm^{-1}) = 3848, 3362, 2962, 2878, 2693, 2501, 2302, 2125, 2031, 1985, 1905, 1837, 1676, 1593, 1558, 1442, 1364, 1289, 1251, 1211, 1065, 987, 898, 852, 801, 740, 681. **Elemental analysis:** $C_{72}H_{72}O_{24}Li_4Ti_2 \cdot 6\ H_2O$: calcd. C = 55.69 %, H = 5.45 %; found C = 55.27 %, H = 5.30 %.

Li[Li$_3$(2^{E2Pent}$_3$Ti)$_2$]

Ligand **2^{E2Pent}**-H_2 (20 mg, 0.09 mmol) is converted into the corresponding complex in methanol (25 mL). The product is obtained after removal of the solvent under reduced pressure as red solid (quantitative). **^{1}H NMR** (600 MHz, MeOH-d_4): Only dimer: δ = 7.09 (dd, *J* = 7.9, 1.5 Hz, 1H, $H_{arom.}$), 6.60-6.50 (m, 2H, $H_{arom.}$), 5.63-5.60 (m, 1H, C*H*), 5.32-5.28 (m, 1H, C*H*), 4.01-3.98 (m, 1H, OCH_2), 3.51-3.45 (m, 1H, OCH_2), 1.97-1.95 (m, 2H, CH_2), 0.91 (t, *J* = 7.4 Hz, 3H, CH_2CH_3). **^{1}H NMR** (600 MHz, DMSO-d_6): Dimer (minor component): δ = 6.95 (dd, *J* = 7.8, 1.5 Hz, 1H, $H_{arom.}$), 6.51 (t, *J* = 7.8 Hz, 1H, $H_{arom.}$), 6.44 (dd, *J* = 7.8, 1.5 Hz, 1H, $H_{arom.}$), 5.35-5.19 (m, 1H, C*H*), 3.93-3.91 (m, 1H, OCH_2), 3.51-3.48 (m, 1H, OCH_2) ppm. Monomer (major component): δ = 6.76 (dd, *J* = 7.8, 1.6 Hz, 1H, $H_{arom.}$), 6.26 (t, *J* = 7.8 Hz, 1H, $H_{arom.}$), 6.12 (dd, *J* = 7.8, 1.6 Hz, 1H, $H_{arom.}$), 4.54 (t, *J* = 6.4 Hz, 2H, OCH_2) ppm. Signals not listed are overlapping and cannot be assigned. **MS** (negative and positive ESI-MS, MeOH, acidified): *m/z* (%) = 1437.3914 (100, [M_D-Li^+], $C_{72}H_{72}O_{24}Li_3Ti_2^-$, calcd. 1437.3853), 1451.4235 (20, [M_D+Li^+], $C_{72}H_{72}O_{24}Li_5Ti_2^+$, calcd. 1451.4173). **IR** (KBr): ṽ (cm^{-1}) = 3631, 3358, 3070, 2963, 2933, 2878, 2688, 2507, 2240, 2159, 2056, 1982, 1941, 1906, 1674, 1595, 1559, 1444, 1381, 1345, 1291, 1252, 1215, 1188, 1154, 1070, 1001, 965, 851, 806, 738, 682. **Elemental analysis:** $C_{72}H_{72}O_{24}Li_4Ti_2 \cdot 5\ H_2O$: calcd. C = 56.34 %, H = 5.39 %; found C = 56.01 %, H = 5.81 %.

Li[Li$_3$(2^{4Pent}$_3$Ti)$_2$]

Ligand **2^{4Pent}**-H_2 (20 mg, 0.09 mmol) is converted into the corresponding complex in methanol (25 mL). The product is obtained after removal of the solvent under reduced pressure as red solid (quantitative). **^{1}H NMR** (600 MHz, MeOH-d_4): Only dimer: δ = 7.11 (dd, *J* = 7.8, 1.6 Hz, 1H, $H_{arom.}$), 6.62-6.50 (m, 2H, $H_{arom.}$), 5.75 (m, 1H, C*H*), 5.02-4.75 (m, 2H, CH_2), 3.65-3.60 (m, 1H, OCH_2), 3.08-3.04 (m, 1H, OCH_2), 1.99-1.95 (m, 2H, CH_2), 1.52-1.49 (m, 2H, CH_2) ppm. **^{1}H NMR** (600 MHz, DMSO-d_6): Dimer (major component): δ = 6.96 (dd, *J* = 7.8, 1.6 Hz, 1H, $H_{arom.}$), 6.50 (t, *J* = 7.8 Hz, 1H, $H_{arom.}$), 6.42 (dd, *J* = 7.8, 1.6 Hz, 1H, $H_{arom.}$), 5.75-5.67 (m, 1H, C*H*), 4.95-4.87 (m, 2H, CH_2), 3.52-3.48 (m, 1H, OCH_2), 2.93-2.90 (m, 1H, OCH_2), 1.99-1.84 (m, 2H, CH_2), 1.52-1.34 (m, 2H, CH_2) ppm. Monomer (minor component): δ = 6.77 (dd, *J* = 7.8, 1.6 Hz, 1H, $H_{arom.}$), 6.26 (t, *J* = 7.8 Hz, 1H, $H_{arom.}$), 6.11 (dd, *J* = 7.8, 1.6 Hz, 1H, $H_{arom.}$), 5.83-5.77 (m, 1H, C*H*), 5.04-4.97 (m, 2H, CH_2), 4.06 (t, *J* = 6.6 Hz, 2H, OCH_2), 2.14-2.10 (m, 2H, CH_2), 1.70-1.67 (m, 2H, CH_2) ppm. **MS** (negative ESI-MS, MeOH, acidified): *m/z* (%) = 1437.3840 (98, [M_D-Li^+], $C_{72}H_{72}O_{24}Li_3Ti_2^-$, calcd. 1437.3853), 715.1830 (100, [M_M-Li^+], $C_{36}H_{36}O_{12}LiTi^-$, calcd. 715.1846). **IR** (KBr): ṽ (cm^{-1}) = 3827, 3353, 3073, 2928, 2652, 2452, 2292, 2239, 2198, 2162, 2075, 2019, 1985, 1943, 1901, 1674, 1596, 1562, 1444, 1390, 1348, 1294, 1252, 1215, 1155, 1067, 1006, 911, 851, 804, 739, 680. **Elemental analysis:** $C_{72}H_{72}O_{24}Li_4Ti_2 \cdot 5\ H_2O$: calcd. C = 56.34 %, H = 5.39 %; found C = 56.14 %, H = 4.95 %.

$Li[Li_3(2^{Z2Hex}{}_3Ti)_2]$

Ligand **2**^{Z2Hex}-H_2 (30 mg, 0.13 mmol) is converted into the corresponding complex in methanol (30 mL). The product is obtained after removal of the solvent under reduced pressure as red solid (quantitative). **^{1}H NMR** (600 MHz, MeOH-d_4): Dimer (major component): δ = 7.10 (dd, J = 7.8, 1.5 Hz, 1H, $H_{arom.}$), 6.59-6.52 (m, 2H, $H_{arom.}$), 5.47-5.39 (m, 1H, C*H*), 5.29-5.25 (m, 1H, C*H*), 4.23-4.21 (m, 1H, OC*H*$_2$), 3.58-3.55 (m, 1H, OC*H*$_2$), 1.95-1.77 (m, 2H, C*H*$_2$), 1.21-1.18 (m, 2H, C*H*$_2$), 0.75 (t, J = 7.4 Hz, 3H, CH_2CH_3) ppm. Monomer (minor component): δ = 7.33 (dd, J = 7.8, 1.6 Hz, 1H, $H_{arom.}$), 7.00 (dd, J = 7.8, 1.6 Hz, 1H, $H_{arom.}$), 6.74 (t, J = 7.8 Hz, 1H, $H_{arom.}$), 5.57-5.55 (m, 1H, C*H*), 5.35-5.32 (m, 1H, C*H*), 4.62 (t, J = 6.8 Hz, 2H, OC*H*$_2$), 2.23-2.20 (m, 2H, C*H*$_2$), 1.36-1.33 (m, 2H, C*H*$_2$), 0.86 (t, J = 7.4 Hz, 3H, CH_2CH_3) ppm. **^{1}H NMR** (600 MHz, DMSO-d_6): Dimer (major component): δ = 6.93 (dd, J = 7.8, 1.6 Hz, 1H, $H_{arom.}$), 6.47 (t, J = 7.8 Hz, 1H, $H_{arom.}$), 6.39 (dd, J = 7.8, 1.6 Hz, 1H, $H_{arom.}$), 5.47-5.35 (m, 1H, C*H*), 5.23-5.20 (m, 1H, C*H*), 4.06-4.03 (m, 1H, OC*H*$_2$), 3.49-3.45 (m, 1H, OC*H*$_2$), 1.90-1.67 (m, 2H, C*H*$_2$), 1.15-1.12 (m, 2H, C*H*$_2$), 0.70 (t, J = 7.4 Hz, 3H, CH_2CH_3) ppm. Monomer (minor component): δ = 6.73 (dd, J = 7.8, 1.6 Hz, 1H $H_{arom.}$), 6.24 (t, J = 7.8 Hz, 1H, $H_{arom.}$), 6.10 (dd, J = 7.8, 1.6 Hz, 1H, $H_{arom.}$), 5.57-5.50 (m, 2H, C*H*C*H*), 4.62 (t, J = 6.8 Hz, 2H, OC*H*$_2$), 2.07-2.03 (m, 2H, C*H*$_2$), 1.35-1.31 (m, 2H, C*H*$_2$), 0.84 (t, J = 7.4 Hz, 3H, CH_2CH_3) ppm. **MS** (negative and positive ESI-MS, MeOH, acidified): *m/z* (%) = 1521.4974 (100, $[M_D\text{-}Li^+]$, $C_{78}H_{84}O_{24}Li_3Ti_2^-$, calcd. 1521.4792), 771.2651 (96, $[M_M\text{+}Li^+]$, $C_{39}H_{42}O_{12}Li_3Ti^+$, calcd. 771.2636), 1535.5154 (100, $[M_D\text{+}Li^+]$, $C_{78}H_{84}O_{24}Li_5Ti_2^+$, calcd. 1535.5112). **IR** (KBr): $\tilde{\nu}$ (cm^{-1}) = 3860, 3748, 3629, 3357, 3069, 3018, 2958, 2930, 2869, 2679, 2499, 2295, 2167, 2141, 2103, 2020, 1977, 1938, 1896, 1843, 1676, 1594, 1561, 1442, 1365, 1291, 1252, 1213, 1187, 1153, 1064, 996, 891, 851, 802, 741, 680. **Elemental analysis:** $C_{78}H_{84}O_{24}Li_4Ti_2 \cdot 16\ H_2O$: calcd. C = 51.55 %, H = 6.43 %; found C = 51.21 %, H = 6.26 %.

$Li[Li_3(2^{E2Hex}{}_3Ti)_2]$

Ligand **2**^{E2Hex}-H_2 (30 mg, 0.13 mmol) is converted into the corresponding complex in methanol (30 mL). The product is obtained after removal of the solvent under reduced pressure as red solid (quantitative). **^{1}H NMR** (600 MHz, MeOH-d_4): Dimer (major component): δ = 7.11 (dd, J = 7.8, 1.5 Hz, 1H, $H_{arom.}$), 6.63-6.50 (m, 2H, $H_{arom.}$), 5.59-5.55 (m, 1H, C*H*), 5.42-5.26 (m, 1H, C*H*), 4.02-3.98 (m, 1H, OC*H*$_2$), 3.51-3.48 (m, 1H, OC*H*$_2$), 1.96-1.92 (m, 2H, C*H*$_2$), 1.35-1.31 (m, 2H, C*H*$_2$), 0.86 (t, J = 7.4 Hz, 3H, CH_2CH_3) ppm. Monomer (minor component): δ = 7.36 (dd, J = 7.8, 1.6 Hz, 1H, $H_{arom.}$), 7.00 (dd, J = 7.8, 1.6 Hz, 1H, $H_{arom.}$), 6.74 (t, J = 7.8 Hz, 1H, $H_{arom.}$), 5.85-5.81 (m, 1H, C*H*), 5.67-5.63 (m, 1H, C*H*), 4.81 (t, J = 6.8 Hz, 2H, OC*H*$_2$), 2.09-2.06 (m, 2H, C*H*$_2$), 1.45-1.41 (m, 2H, C*H*$_2$), 0.94 (t, J = 7.4 Hz, 3H, CH_2CH_3) ppm. **^{1}H NMR** (400 MHz, DMSO-d_6): Dimer (major component): δ = 6.95 (dd, J = 7.8, 1.6 Hz, 1H, $H_{arom.}$), 6.47 (t, J = 7.8 Hz, 1H, $H_{arom.}$), 6.41 (dd, J = 7.8, 1.6 Hz, 1H, $H_{arom.}$), 5.60-5.54 (m, 1H, C*H*), 5.30-5.22 (m, 1H, C*H*), 3.92-3.88 (m, 1H, OC*H*$_2$), 3.39-3.35 (m, 1H, OC*H*$_2$), 1.90-1.87 (m, 2H, C*H*$_2$), 1.27-1.24 (m, 2H, C*H*$_2$), 0.78 (t, J = 7.4 Hz, 3H, CH_2CH_3) ppm. Monomer (minor component): δ = 6.76 (dd, J = 7.8, 1.6 Hz, 1H, $H_{arom.}$), 6.23 (t, J = 7.8 Hz, 1H, $H_{arom.}$), 6.09 (dd, J = 7.8, 1.6 Hz, 1H, $H_{arom.}$), 5.79-5.72 (m, 1H, C*H*), 5.53-5.48 (m, 1H, C*H*), 4.52 (t, J = 6.8 Hz, 2H, OC*H*$_2$), 1.95-1.93 (m, 2H, C*H*$_2$), 1.32-1.29 (m, 2H, C*H*$_2$), 0.82 (t, J = 7.4 Hz, 3H, CH_2CH_3) ppm. **MS** (negative and positive ESI-MS, MeOH, acidified): *m/z* (%) = 1521.5050 (100, $[M_D\text{-}Li^+]$, $C_{78}H_{84}O_{24}Li_3Ti_2^-$, calcd. 1521.4792), 757.2394 (20, $[M_M\text{-}Li^+]$, $C_{39}H_{42}O_{12}LiTi^-$, calcd. 757.2316), 1535.5117 (50, $[M_D\text{+}Li^+]$, $C_{78}H_{84}O_{24}Li_5Ti_2^+$, calcd. 1535.5112). **IR** (KBr): $\tilde{\nu}$ (cm^{-1}) = 3897, 3856, 3747, 3625, 3360, 3068, 2957, 2929, 2871, 2684, 2515, 2286, 2161, 2112, 2019, 1984, 1951, 1844, 1674, 1594, 1559, 1443, 1382, 1345, 1291, 1251, 1214, 1188, 1152, 1067, 992, 968, 891, 852, 805, 738, 681. **Elemental analysis:** $C_{78}H_{84}O_{24}Li_4Ti_2 \cdot 18\ H_2O$: calcd. C = 50.55 %, H = 6.53 %; found C = 50.45 %, H = 6.54 %.

$Li[Li_3(2^{Z3Hex}{}_3Ti)_2]$

Ligand **2^{Z3Hex}**-H_2 (30 mg, 0.13 mmol) is converted into the corresponding complex in methanol (30 mL). The product is obtained after removal of the solvent under reduced pressure as red solid (quantitative). **^{1}H NMR** (600 MHz, MeOH-d_4): Dimer (major component): δ = 7.10 (dd, J = 7.9, 1.6 Hz, 1H, $H_{arom.}$), 6.57-6.53 (m, 2H, $H_{arom.}$), 5.41-5.37 (m, 1H, CH), 5.22-5.14 (m, 1H, CH), 3.57-3.53 (m, 1H, OCH_2), 3.10-3.07 (m, 1H, OCH_2), 2.15-2.07 (m, 2H, CH_2), 1.96-1.93 (m, 2H, CH_2), 0.87 (t, J = 7.5 Hz, 3H, CH_2CH_3) ppm. Monomer (minor component): δ = 7.41 (dd, J = 7.9, 1.5 Hz, 1H, $H_{arom.}$), 7.00 (dd, J = 7.9, 1.5 Hz, 1H, $H_{arom.}$), 6.84 (t, J = 7.9 Hz, 1H, $H_{arom.}$), 5.53-5.51 (m, 1H, CH), 5.47-5.45 (m, 1H, CH), 3.85 (t, J = 6.8 Hz, 2H, OCH_2), 2.55-2.53 (m, 2H, CH_2), 2.28-2.26 (m, 2H, CH_2), 0.97 (t, J = 7.4 Hz, 3H, CH_2CH_3) ppm. **^{1}H NMR** (400 MHz, DMSO-d_6): Dimer (major component): δ = 6.93 (dd, J = 7.9, 1.6 Hz, 1H, $H_{arom.}$), 6.46 (t, J = 7.9 Hz, 1H, $H_{arom.}$), 6.37 (dd, J = 7.9, 1.6 Hz, 1H, $H_{arom.}$), 3.43-3.41 (m, 1H, OCH_2), 2.92-2.89 (m, 1H, OCH_2), 0.81 (t, J = 7.4 Hz, 3H, CH_2CH_3) ppm. Monomer (minor component): δ = 6.73 (dd, J = 7.9, 1.6 Hz, 1H, $H_{arom.}$), 6.23 (t, J = 7.9 Hz, 1H, $H_{arom.}$), 6.10 (dd, J = 7.9, 1.6 Hz, 1H, $H_{arom.}$), 4.02 (t, J = 6.8 Hz, 2H, OCH_2), 0.88 (t, J = 7.4 Hz, 3H, CH_2CH_3) ppm. Signals not listed are overlapping and cannot be assigned. **MS** (negative and positive ESI-MS, MeOH): *m/z* (%) = 1521.5096 (100, [M_D-Li^+], $C_{78}H_{84}O_{24}Li_3Ti_2^-$, calcd. 1521.4792), 1529.5055 (70, [M_D+H^+], $C_{78}H_{85}O_{24}Li_4Ti_2^+$, calcd. 1529.4950). **IR** (KBr): $\tilde{\nu}$ (cm^{-1}) = 3898, 3859, 3748, 3393, 3068, 3010, 2962, 2688, 2506, 2303, 2167, 2109, 2022, 1982, 1938, 1839, 1794, 1745, 1676, 1594, 1560, 1444, 1386, 1344, 1296, 1252, 1214, 1154, 1065, 1009, 906, 854, 806, 740, 682. **Elemental analysis:** $C_{78}H_{84}O_{24}Li_4Ti_2 \cdot 14\ H_2O$: calcd. C = 52.60 %, H = 6.34 %; found C = 52.03 %, H = 6.03 %.

$Li[Li_3(2^{E3Hex}{}_3Ti)_2]$

Ligand **2^{E3Hex}**-H_2 (30 mg, 0.13 mmol) is converted into the corresponding complex in methanol (30 mL). The product is obtained after removal of the solvent under reduced pressure as red solid (quantitative). **^{1}H NMR** (600 MHz, MeOH-d_4): Dimer (major component): δ = 7.11 (dd, J = 7.8, 1.5 Hz, 1H, $H_{arom.}$), 6.60-6.55 (m, 2H, $H_{arom.}$), 5.45-5.42 (m, 1H, CH), 5.36-5.25 (m, 1H, CH), 3.61-3.58 (m, 1H, OCH_2), 3.08-3.02 (m, 1H, OCH_2), 2.15-2.05 (m, 2H, CH_2), 2.00-1.97 (m, 2H, CH_2), 0.96 (t, J = 7.4 Hz, 3H, CH_2CH_3) ppm. Monomer (minor component): δ = 7.36 (dd, J = 7.8, 1.5 Hz, 1H, $H_{arom.}$), 7.01 (dd, J = 7.8, 1.5 Hz, 1H, $H_{arom.}$), 6.76 (t, J = 7.8 Hz, 1H, $H_{arom.}$), 5.62-5.59 (m, 1H, CH), 5.41-5.38 (m, 1H, CH), 4.38 (t, J = 6.8 Hz, 2H, OCH_2), 2.43-2.41 (m, 2H, CH_2), 2.21-2.17 (m, 2H, CH_2), 1.03 (t, J = 7.4 Hz, 3H, CH_2CH_3) ppm. **^{1}H NMR** (400 MHz, DMSO-d_6): Dimer (major component): δ = 6.94 (dd, J = 7.8, 1.6 Hz, 1H, $H_{arom.}$), 6.47 (t, J = 7.8 Hz, 1H, $H_{arom.}$), 6.37 (dd, J = 7.8, 1.6 Hz, 1H, $H_{arom.}$), 3.45-3.43 (m, 1H, OCH_2), 2.92-2.81 (m, 1H, OCH_2), 0.86 (t, J = 7.4 Hz, 3H, CH_2CH_3) ppm. Monomer (minor component): δ = 6.73 (dd, J = 7.8, 1.6 Hz, 1H, $H_{arom.}$), 6.22 (t, J = 7.8 Hz, 1H, $H_{arom.}$), 6.09 (dd, J = 7.8, 1.5 Hz, 1H, $H_{arom.}$), 4.02 (t, J = 6.8 Hz, 2H, OCH_2), 0.89 (t, J = 7.4 Hz, 3H, CH_2CH_3) ppm. Signals not listed are overlapping and cannot be assigned. **MS** (negative and positive ESI-MS, MeOH, acidified): *m/z* (%) = 1521.4977 (100, [M_D-Li^+], $C_{78}H_{84}O_{24}Li_3Ti_2^-$, calcd. 1521.4792), 1535.5120 (40, [M_D+Li^+], $C_{78}H_{84}O_{24}Li_5Ti_2^+$, calcd. 1535.5112). **IR** (KBr): $\tilde{\nu}$ (cm^{-1}) = 3898, 3856, 3628, 3357, 3067, 3021, 2924, 2852, 2663, 2519, 2320, 2239, 2157, 2106, 2059, 2025, 1984, 1938, 1844, 1791, 1676, 1594, 1560, 1444, 1388, 1349, 1294, 1254, 1216, 1154, 1068, 1012, 965, 915, 890, 853, 804, 739, 682. **Elemental analysis** $C_{78}H_{84}O_{24}Li_4Ti_2 \cdot 4\ H_2O$: calcd. C = 58.51 %, H = 5.79 %; found C = 58.29 %, H = 5.65 %.

$Li[Li_3(2^{Z4Hex}{}_3Ti)_2]$

Ligand **2^{Z4Hex}**-H_2 (30 mg, 0.13 mmol) is converted into the corresponding complex in methanol (30 mL). The product is obtained after removal of the solvent under reduced pressure as red solid

(quantitative). ^{1}**H NMR** (600 MHz, MeOH-*d*$_4$): Only dimer (major component): δ = 7.13 (dd, *J* = 7.8, 1.5 Hz, 1H, $H_{arom.}$), 6.60-6.53 (m, 2H, $H_{arom.}$), 5.44-5.35 (m, 1H, C*H*), 5.32-5.29 (m, 1H, C*H*), 3.62-3.60 (m, 1H, OC*H*$_2$), 3.06-3.04 (m, 1H, OC*H*$_2$), 2.01-1.98 (m, 2H, C*H*$_2$), 1.52-1.48 (m, 2H, C*H*$_2$), 1.45-1.40 (m, 3H, CHC*H*$_3$) ppm. ^{1}**H NMR** (400 MHz, DMSO-*d*$_6$): Dimer (major component): δ = 6.99 (dd, *J* = 7.9, 1.5 Hz, 1H, $H_{arom.}$), 6.47 (t, *J* = 7.9 Hz, 1H, $H_{arom.}$), 6.41 (dd, *J* = 7.9, 1.5 Hz, 1H, $H_{arom.}$), 5.35-5.21 (m, 2H, C*H*C*H*), 3.55-3.48 (m, 1H, OC*H*$_2$), 2.88-2.82 (m, 1H, OC*H*$_2$), 1.95-1.90 (m, 2H, C*H*$_2$) ppm. Monomer (minor component): δ = 6.78 (dd, *J* = 7.9, 1.5 Hz, 1H, $H_{arom.}$), 6.23 (t, *J* = 7.9 Hz, 1H, $H_{arom.}$), 6.09 (dd, *J* = 7.9, 1.5 Hz, 1H, $H_{arom.}$), 5.41-5.36 (m, 2H, C*H*C*H*), 4.03 (t, *J* = 6.8 Hz, 2H, OC*H*$_2$), 2.11-2.04 (m, 2H, C*H*$_2$), 1.65-1.61 (m, 2H, C*H*$_2$) ppm. Signals not listed are overlapping and cannot be assigned. **MS** (negative ESI-MS, MeOH, acidified): *m/z* (%) = 1521.5057 (100, [M_D-Li^+], $C_{78}H_{84}O_{24}Li_3Ti_2^-$, calcd. 1521.4792). **IR** (KBr): $\tilde{\nu}$ (cm^{-1}) = 3857, 3747, 3627, 3356, 3069, 3012, 2922, 2858, 2684, 2511, 2297, 2250, 2164, 2102, 2027, 1981, 1956, 1842, 1674, 1594, 1559, 1443, 1388, 1348, 1292, 1252, 1214, 1153, 1069, 1022, 996, 891, 852, 803, 738, 679. **Elemental analysis**: $C_{78}H_{84}O_{24}Li_4Ti_2 \cdot 14\ H_2O$: calcd. C = 52.60 %, H = 6.34 %; found C = 52.67 %, H = 5.95 %.

Li[Li$_3$(2^{E4Hex}$_3$Ti)$_2$]

Ligand **2**$^{\mathbf{E4Hex}}$-H_2 (30 mg, 0.13 mmol) is converted into the corresponding complex in methanol (30 mL). The product is obtained after removal of the solvent under reduced pressure as red solid (quantitative). ^{1}**H NMR** (600 MHz, MeOH-*d*$_4$): Only dimer: δ = 7.09 (dd, *J* = 7.8, 1.5 Hz, 1H, $H_{arom.}$), 6.60-6.52 (m, 2H, $H_{arom.}$), 5.41-5.32 (m, 2H, C*H*C*H*), 3.62-3.60 (m, 1H, OC*H*$_2$), 3.05-3.03 (m, 1H, OC*H*$_2$), 2.01-1.98 (m, 2H, C*H*$_2$), 1.51-1.48 (m, 2H, C*H*$_2$), 1.44-1.41 (m, 3H, CHC*H*$_3$) ppm. ^{1}**H NMR** (400 MHz, DMSO-*d*$_6$): Dimer (major component): δ = 6.97 (dd, *J* = 7.8, 1.6 Hz, 1H, $H_{arom.}$), 6.45 (t, *J* = 7.8 Hz, 1H, $H_{arom.}$), 6.39 (dd, *J* = 7.8, 1.6 Hz, 1H, $H_{arom.}$), 5.27-5.23 (m, 2H, C*H*C*H*), 3.51-3.48 (m, 1H, OC*H*$_2$), 2.87-2.84 (m, 1H, OC*H*$_2$) ppm. Monomer (minor component): δ = 6.78 (dd, *J* = 7.8, 1.6 Hz, 1H, $H_{arom.}$), 6.22 (t, *J* = 7.8 Hz, 1H, $H_{arom.}$), 6.06 (dd, *J* = 7.8, 1.6 Hz, 1H, $H_{arom.}$), 5.42-5.37 (m, 2H, C*H*C*H*), 4.02 (t, *J* = 6.5 Hz, 2H, OC*H*$_2$) ppm. Signals not listed are overlapping and cannot be assigned. **MS** (negative and positive ESI-MS, MeOH, acidified): *m/z* (%) = 1521.4898 (100, [M_D-Li^+], $C_{78}H_{84}O_{24}Li_3Ti_2^-$, calcd. 1521.4792), 1535.5156 (90, [M_D+Li^+], $C_{78}H_{84}O_{24}Li_5Ti_2^+$, calcd. 1535.5112). **IR** (KBr): $\tilde{\nu}$ (cm^{-1}) = 3748, 3547, 3069, 2930, 2853, 2503, 2161, 2016, 1983, 1910, 1673, 1594, 1559, 1444, 1390, 1345, 1295, 1252, 1215, 1151, 1068, 1010, 965, 917, 890, 852, 806, 741, 683. **Elemental analysis:** $C_{78}H_{84}O_{24}Li_4Ti_2 \cdot 20\ H_2O$: calcd. C = 49.59 %, H = 6.62 %; found C = 49.68 %, H = 6.59 %.

Li[Li$_3$(2^{5Hex}$_3$Ti)$_2$]

Ligand **2**$^{\mathbf{5Hex}}$-H_2 (30 mg, 0.13 mmol) is converted into the corresponding complex in methanol (30 mL). The product is obtained after removal of the solvent under reduced pressure as red solid (quantitative). ^{1}**H NMR** (600 MHz, MeOH-*d*$_4$): Only dimer: δ = 7.10 (dd, *J* = 7.9, 1.5 Hz, 1H, $H_{arom.}$), 6.60-6.53 (m, 2H, $H_{arom.}$), 5.79-5.75 (m, 1H, C*H*), 4.96-4.88 (m, 2H, C*H*$_2$), 3.61-3.59 (m, 1H, OC*H*$_2$), 3.03-3.01 (m, 1H, OC*H*$_2$), 2.02-1.98 (m, 2H, C*H*$_2$), 1.51-1.35 (m, 2H, C*H*$_2$), 1.34-1.26 (m, 2H, C*H*$_2$) ppm. ^{1}**H NMR** (400 MHz, DMSO-*d*$_6$): Dimer (major component): δ = 6.95 (dd, *J* = 7.8, 1.6 Hz, 1H, $H_{arom.}$), 6.47 (t, *J* = 7.8 Hz, 1H, $H_{arom.}$), 6.39 (dd, *J* = 7.8, 1.6 Hz, 1H, $H_{arom.}$), 5.68-5.63 (m, 1H, C*H*), 4.93-4.85 (m, 2H, C*H*$_2$), 3.50-3.45 (m, 1H, OC*H*$_2$), 2.94-2.85 (m, 1H, OC*H*$_2$), 1.97-1.93 (m, 2H, C*H*$_2$) ppm. Monomer (minor component): δ = 6.76 (dd, *J* = 7.8, 1.6 Hz, 1H, $H_{arom.}$), 6.23 (t, *J* = 7.8 Hz, 1H, $H_{arom.}$), 6.09 (dd, *J* = 7.8, 1.6 Hz, 1H, $H_{arom.}$), 5.78-5.65 (m, 1H, C*H*), 5.01-4.95 (m, 2H, C*H*$_2$), 4.02 (t, *J* = 6.8 Hz, 2H, OC*H*$_2$), 2.04-1.98 (m, 2H, C*H*$_2$), 1.62-1.57 (m, 2H, C*H*$_2$) ppm. Signals not listed are overlapping and cannot be assigned. **MS** (positive ESI-MS, MeOH, acidified): *m/z* (%) = 1535.5156 (50, [M_D+Li^+], $C_{78}H_{84}O_{24}Li_5Ti_2^+$,

calcd. 1535.5112). **IR** (KBr): ṽ (cm^{-1}) = 3857, 3747, 3628, 3358, 3071, 2931, 2860, 2682, 2509, 2322, 2221, 2164, 2105, 2020, 1983, 1949, 1891, 1840, 1676, 1594, 1560, 1444, 1391, 1345, 1295, 1253, 1215, 1154, 1066, 996, 908, 854, 803, 740, 682. **Elemental analysis:** $C_{78}H_{84}O_{24}Li_4Ti_2 \cdot 20\ H_2O$: calcd. C = 49.59 %, H = 6.62 %; found C = 49.75 %, H = 6.48 %.

Li[Li$_3$(2$^{6Hept}{}_3$Ti)$_2$]

Ligand **2^{6Hept}**-H$_2$ (50 mg, 0.2 mmol) is converted into the corresponding complex in methanol (50 mL). The product is obtained after removal of the solvent under reduced pressure as red solid (quantitative). **^{1}H NMR** (600 MHz, MeOH-*d*$_4$): Only dimer: *δ* = 7.10 (dd, *J* = 7.3, 2.4 Hz, 1H, H$_{arom.}$), 6.59-6.52 (m, 2H, H$_{arom.}$), 5.77 (m, 1H, C*H*), 5.00-4.86 (m, 2H, C*H*$_2$), 3.69-3.55 (m, 1H, OC*H*$_2$), 3.05-3.01 (m, 1H, OC*H*$_2$), 2.03-2.00 (m, 2H, C*H*$_2$), 1.43-1.20 (m, 6H, 3×C*H*$_2$) ppm. **^{1}H NMR** (400 MHz, DMSO-*d*$_6$): Dimer (major component): *δ* = 6.92 (dd, *J* = 7.8, 1.6 Hz, 1H, H$_{arom.}$), 6.46 (t, *J* =7.8 Hz, 1H, H$_{arom.}$), 6.38 (dd, *J* =7.8, 1.6 Hz, 1H, H$_{arom.}$), 4.93-4.86 (m, 2H, C*H*$_2$), 3.48-3.44 (m, 1H, OC*H*$_2$), 2.87-2.83 (m, 1H, OC*H*$_2$) ppm. Monomer (minor component): *δ* = 6.74 (dd, *J* = 7.8, 1.6 Hz, 1H, H$_{arom.}$), 6.24 (t, *J* = 7.8 Hz, 1H, H$_{arom.}$), 6.10 (dd, *J* =7.8, 1.6 Hz, 1H, H$_{arom.}$), 5.01-4.93 (m, 2H, C*H*$_2$), 4.03 (t, *J* = 6.6 Hz, 2H, OC*H*$_2$) ppm. Signals not listed are overlapping and cannot be assigned. **MS** (negative and positive ESI-MS, MeOH): *m/z* (%) = 1605.5642 (100, [M$_D$-Li$^+$], $C_{84}H_{96}O_{24}Li_3Ti_2^+$, calcd. 1605.5731), 1619.6094 (10, [M$_D$+Li$^+$], $C_{84}H_{96}O_{24}Li_5Ti_2^+$, calcd. 1619.6052). **IR** (KBr): ṽ (cm^{-1}) = 3358, 3071, 2928, 2857, 2601, 2165, 2043, 1979, 1829, 1676, 1595, 1522, 1444, 1391, 1348, 1295, 1254, 1214, 1154, 1066, 994, 910, 855, 804, 739, 680. **Elemental analysis:** $C_{84}H_{96}O_{24}Li_4Ti_2 \cdot 10\ H_2O$: calcd. C = 56.26 %, H = 6.52 %; found C = 56.52 %, H = 5.99 %.

Li[Li$_3$(2$^{7Oct}{}_3$Ti)$_2$]

Ligand **2^{7Oct}**-H$_2$ (50 mg, 0.19 mmol) is converted into the corresponding complex in methanol (50 mL). The product is obtained after removal of the solvent under reduced pressure as red solid (quantitative). **^{1}H NMR** (600 MHz, MeOH-*d*$_4$): Only dimer: *δ* = 7.10 (dd, *J* = 7.5, 2.2 Hz, 1H, H$_{arom.}$), 6.60-6.52 (m, 2H, H$_{arom.}$), 5.79-5.77 (m, 1H, C*H*), 4.97-4.88 (m, 2H, C*H*$_2$), 3.60-3.58 (m, 1H, OC*H*$_2$), 3.03-3.01 (m, 1H, OC*H*$_2$), 2.17-2.13 (m, 2H, C*H*$_2$), 2.09-1.98 (m, 2H, C*H*$_2$), 1.49-1.21 (m, 6H, 3×C*H*$_2$) ppm. **^{1}H NMR** (400 MHz, DMSO-*d*$_6$): Dimer (major component): *δ* = 6.92 (dd, *J* = 7.8, 1.6 Hz, 1H, H$_{arom.}$), 6.47 (t, *J* = 7.8 Hz, 1H, H$_{arom.}$), 6.38 (dd, *J* =7.8, 1.6 Hz, 1H, H$_{arom.}$), 4.92-4.86 (m, 2H, C*H*$_2$), 3.47-3.45 (m, 1H, OC*H*$_2$), 2.87-2.84 (m, 1H, OC*H*$_2$) ppm. Monomer (minor component): δ = 6.72 (dd, *J* = 7.8, 1.6 Hz, 1H, H$_{arom.}$), 6.22 (t, *J* = 7.8 Hz, 1H, H$_{arom.}$), 6.07 (dd, *J* = 7.8, 1.6 Hz, 1H, H$_{arom.}$), 5.00-4.93 (m, 2H, C*H*$_2$), 4.02 (t, *J* = 6.6 Hz, 2H, OC*H*$_2$) ppm. Signals not listed are overlapping and cannot be assigned. **MS** (negative and positive ESI-MS, MeOH): *m/z* (%) = 1689.6698 (100, [M$_D$-Li$^+$], $C_{90}H_{108}O_{24}Li_3Ti_2^+$, calcd. 1689.6670), 1703.7036 (30, [M$_D$+Li$^+$], $C_{90}H_{108}O_{24}Li_5Ti_2^+$, calcd. 1703.6990). **IR** (KBr): ṽ (cm^{-1}) = 3359, 3072, 2927, 2855, 2599, 2285, 2167, 2012, 1831, 1676, 1594, 1560, 1444, 1390, 1346, 1294, 1253, 1215, 1154, 1067, 994, 907, 854, 805, 738, 681. **Elemental analysis:** $C_{90}H_{108}O_{24}Li_4Ti_2 \cdot 5\ H_2O$: calcd. C = 60.48 %, H = 6.65 %; found C = 60.01 %, H = 6.42 %.

Li[Li$_3$(2$^{8Non}{}_3$Ti)$_2$]

Ligand **2^{8Non}**-H$_2$ (50 mg, 0.18 mmol) is converted into the corresponding complex in methanol (50 mL). The product is obtained after removal of the solvent under reduced pressure as red solid (quantitative). **^{1}H NMR** (600 MHz, MeOH-*d*$_4$): Only dimer: *δ* = 7.09 (dd, *J* = 7.9, 1.6 Hz, 1H, H$_{arom.}$), 6.58-6.48 (m, 2H, H$_{arom.}$), 5.85-5.70 (m, 1H, C*H*), 5.00-4.81 (m, 2H, C*H*$_2$), 3.63-3.49 (m, 1H, OC*H*$_2$), 3.07-

2.98 (m, 1H, OCH_2), 2.03-1.96 (m, 2H, CH_2), 1.43-1.30 (m, 4H, 2×CH_2), 1.29-1.21 (m, 6H 3×CH_2) ppm. ^{1}**H NMR** (400 MHz, DMSO-d_6): Dimer (major component): δ = 6.92 (dd, J = 8.2, 1.6 Hz, 1H, $H_{arom.}$), 6.46 (t, J = 8.2 Hz, 1H, $H_{arom.}$), 6.38 (dd, J = 8.2, 1.6 Hz, 1H, $H_{arom.}$), 3.46-3.44 (m, 1H, OCH_2), 2.87-2.85 (m, 1H, OCH_2) ppm. Monomer (minor component): δ = 6.72 (dd, J = 8.2, 1.6 Hz, 1H, $H_{arom.}$), 6.21 (t, J = 8.2 Hz, 1H, $H_{arom.}$), 6.08 (dd, J = 8.2, 1.6 Hz, 1H, $H_{arom.}$), 4.02 (t, J = 6.6 Hz, 2H, OCH_2) ppm. Signals not listed are overlapping and cannot be assigned. **MS** (negative and positive ESI-MS, MeOH): m/z (%) = 1773.7756 (100, [M_D-Li^+], $C_{96}H_{120}O_{24}Li_3Ti_2^-$, calcd. 1773.7609), 1787.7924 (50, [M_D+Li^+], $C_{96}H_{120}O_{24}Li_5Ti_2^+$, calcd. 1787.7928). **IR** (KBr): $\tilde{\nu}$ (cm^{-1}) = 3901, 3366, 3071, 2925, 2854, 2657, 2507, 2322, 2229, 2162, 2057, 2005, 1961, 1903, 1781, 1677, 1593, 1523, 1445, 1390, 1294, 1253, 1215, 1154, 1065, 999, 907, 855, 803, 738, 682. **Elemental analysis:** $C_{96}H_{120}O_{24}Li_4Ti_2 \cdot 14\ H_2O$: calcd. C = 56.70 %, H = 7.34 %; found C = 56.20 %, H = 6.68 %.

Li[Li$_3$(2^{9Dec}$_3$Ti)$_2$]

Ligand **2^{9Dec}**-H_2 (50 mg, 0.17 mmol) is converted into the corresponding complex in methanol (50 mL). The product is obtained after removal of the solvent under reduced pressure as red solid (quantitative). ^{1}**H NMR** (600 MHz, MeOH-d_4): Dimer (major component): δ = 7.08 (dd, J = 7.9, 1.5 Hz, 1H, $H_{arom.}$), 6.58-6.51 (m, 2H, $H_{arom.}$), 5.82-5.74 (m, 1H, CH), 5.00-4.87 (m, 2H, CH_2), 3.60-3.49 (m, 1H, OCH_2), 3.06-2.97 (m, 1H, OCH_2), 2.04-1.97 (m, 2H, CH_2), 1.40-1.28 (m, 4H, 2×CH_2), 1.27-1.20 (m, 8H, 4×CH_2) ppm. Monomer (minor component): δ = 7.38 (dd, J = 7.9, 1.5 Hz, 1H, $H_{arom.}$), 7.00 (dd, J = 7.9, 1.5 Hz, 1H, $H_{arom.}$), 6.78 (t, J = 7.9 Hz, 1H, $H_{arom.}$), 4.38 (t, J = 6.6 Hz, 2H, OCH_2), 3.59-3.55 (m, 2H, CH_2), 1.79-1.75 (m, 4H, 2×CH_2), 1.48-1.43 (m, 8H, 4×CH_2) ppm. Signals not listed are overlapping and cannot be assigned. ^{1}**H NMR** (400 MHz, DMSO-d_6): Dimer (major component): δ = 6.92 (dd, J = 7.8, 1.5 Hz, 1H, $H_{arom.}$), 6.45 (t, J = 7.8 Hz, 1H, $H_{arom.}$), 6.38 (dd, J = 7.8, 1.5 Hz, 1H, $H_{arom.}$), 3.47-3.45 (m, 1H, OCH_2), 2.87-2.85 (m, 1H, OCH_2) ppm. Monomer (minor component): δ = 6.72 (dd, J = 7.8, 1.5 Hz, 1H, $H_{arom.}$), 6.21 (t, J = 7.8 Hz, 1H, $H_{arom.}$), 6.08 (dd, J = 7.8, 1.5 Hz, 1H, $H_{arom.}$), 4.04 (t, J = 6.6 Hz, 2H, OCH_2) ppm. Signals not listed are overlapping and cannot be assigned. **MS** (negative and positive ESI-MS, MeOH): m/z (%) = 1858.8671 (100, [M_D-Li^+], $C_{102}H_{132}O_{24}Li_3Ti_2^-$, calcd. 1858.6330), 1872.8884 (10, [M_D+Li^+], $C_{102}H_{132}O_{24}Li_5Ti_2^+$, calcd. 1872.6640). **IR** (KBr): $\tilde{\nu}$ (cm^{-1}) = 3395, 3072, 2924, 2854, 2595, 2508, 2448, 2229, 2163, 2041, 1987, 1904, 1828, 1676, 1594, 1561, 1445, 1390, 1346, 1294, 1253, 1215, 1154, 1064, 997, 908, 854, 801, 739, 681. **Elemental analysis:** $C_{102}H_{132}O_{24}Li_4Ti_2 \cdot 10\ H_2O$: calcd. C = 59.88 %, H = 7.49 %; found C = 59.89 %, H = 7.59 %.

Li[Li$_3$(2^{10Undec}$_3$Ti)$_2$]

Ligand **2^{10Undec}**-H_2 (40 mg, 0.13 mmol) is converted into the corresponding complex in methanol (25 mL). The product is obtained after removal of the solvent under reduced pressure as red solid (quantitative). ^{1}**H NMR** (400 MHz, MeOH-d_4): Only dimer: δ = 7.09 (dd, J = 7.9, 1.4 Hz, 1H, $H_{arom.}$), 6.59-6.48 (m, 2H, $H_{arom.}$), 5.82-5.79 (m, 1H, CH), 4.96-4.81 (m, 2H, CHCH_2), 3.64-3.49 (m, 1H, OCH_2), 3.06-2.95 (m, 1H, OCH_2), 2.02 (q, J = 7.0 Hz, 2H, CH_2), 1.40-1.32 (m, 4H, 2×CH_2), 1.32-1.17 (m, 10H, 5×CH_2) ppm. ^{1}**H NMR** (400 MHz, DMSO-d_6): Dimer (major component): δ = 6.92 (dd, J = 7.8, 1.5 Hz, 1H, $H_{arom.}$), 6.45 (t, J = 7.8 Hz, 1H, $H_{arom.}$), 6.38 (dd, J = 7.8, 1.5 Hz, 1H, $H_{arom.}$), 3.47-3.45 (m, 1H, OCH_2), 2.87-2.85 (m, 1H, OCH_2) ppm. Monomer (minor component): δ = 6.72 (dd, J = 7.8, 1.5 Hz, 1H, $H_{arom.}$), 6.21 (t, J = 7.8 Hz, 1H, $H_{arom.}$), 6.08 (dd, J = 7.8, 1.5 Hz, 1H, $H_{arom.}$), 4.04 (t, J = 6.6 Hz, 2H, OCH_2) ppm. Signals not listed are overlapping and cannot be assigned. **MS** (negative and positive ESI-MS, MeOH): m/z (%) = 1942.9647 (100, [M_D-Li^+], $C_{108}H_{144}O_{24}Li_3Ti_2^-$, calcd. 1942.7940), 1956.9817 (10, [M_D+Li^+], $C_{108}H_{144}O_{24}Li_5Ti_2^+$, calcd. 1956.8262). **IR** (KBr): $\tilde{\nu}$ (cm^{-1}) = 3360, 3072, 2924, 2853, 2686, 2222, 2159,

2039, 1899, 1829, 1676, 1594, 1560, 1445, 1390, 1346, 1294, 1253, 1215, 1192, 1154, 1066, 996, 907, 855, 805, 739, 681. **Elemental analysis:** $C_{108}H_{144}O_{24}Li_4Ti_2 \cdot 6\ H_2O$: calcd. C = 63.03 %, H = 7.64 %; found C = 63.26 %, H = 7.40 %.

Alkinyl ester ligands

Ligands 3-H$_2$

Propargyl-2,3-dihydroxybenzoate (3^{Prop}-H$_2$)

Propargyl alcohol (218.25 mg, 3.89 mmol) is converted into the corresponding ester using a modification of the general procedure. 3 equivalents of the alcohol and 6 equivalents of NEt_3 are used and the reaction mixture was refluxed for 3 days. The product is obtained after purification via column chromatography (pentane/ethyl acetate 12:1, R_f = 0.26) as white solid (36 %, 90 mg, 0.47 mmol). **M.p.:** 86 °C – 88 °C (last solvent used: DCM). **^{1}H NMR** (600 MHz, $CDCl_3$): δ = 10.62 (s, 1H, O*H*), 7.41 (dd, *J* = 8.0, 1.5 Hz, 1H, $H_{arom.}$), 7.12 (dd, *J* = 8.0, 1.5 Hz, 1H, $H_{arom.}$), 6.81 (t, *J* = 8.0 Hz, 1H, $H_{arom.}$), 5.65 (s, 1H, O*H*), 4.97 (d, *J* = 2.5 Hz, 2H, OC*H*$_2$), 2.58 (t, *J* = 2.5 Hz, 1H, C*H*) ppm. **^{13}C NMR** (151 MHz, $CDCl_3$): δ = 169.52 (*C*O$_2$CH$_2$), 148.93 ($C_{arom.}$), 145.02 ($C_{arom.}$), 120.76 ($C_{arom.}$), 120.27 ($C_{arom.}$), 119.43 ($C_{arom.}$), 111.83 ($C_{arom.}$), 76.79 (CH$_2$*C*), 75.693 (*C*CH), 52.874 (O*C*H$_2$C) ppm. **MS** (negative and positive ESI-MS, MeOH, acidified): *m/z* (%) = 191.0356 (20, [M-H$^+$], $C_{10}H_7O_4^-$, calcd. 191.0126), 215.0324 (30, [M+Na$^+$] $C_{10}H_8O_4Na^+$, calcd. 215.0320). **IR** (KBr): $\tilde{\nu}$ (cm^{-1}) = 3819, 3492, 3267, 2940, 2455, 2323, 2205, 2133, 2087, 2031, 1985, 1934, 1875, 1819, 1674, 1617, 1468, 1389, 1302, 1258, 1144, 1066, 1006, 962, 915, 841, 781, 753, 726, 698, 663. **Elemental Analysis**: $C_{10}H_8O_4$: calcd. C = 62.50 %, H = 4.20 %; found C = 62.29 %, H = 4.27 %.

But-2-yn-2,3-dihydroxybenzoate (3^{2Bu}-H$_2$)

2-Butyn-1-ol (272.86 mg, 3.89 mmol) is converted into the corresponding ester using a modification of the general procedure. 3 equivalents of the alcohol and 6 equivalents of NEt_3 are used and the reaction mixture was refluxed for 3 days. The product is obtained after purification via column chromatography (pentane/ethyl acetate 12:1, R_f = 0.26) as white solid (28 %, 76 mg, 0.36 mmol). **M.p.**: 88 °C – 90 °C (last solvent used: DCM). **^{1}H NMR** (600 MHz, $CDCl_3$): δ = 10.74 (s, 1H, O*H*), 7.42 (dd, *J* = 8.0, 1.5 Hz, 1H, $H_{arom.}$), 7.12 (dd, *J* = 8.0, 1.5 Hz, 1H, $H_{arom.}$), 6.81 (t, *J* = 8.0 Hz, 1H, $H_{arom.}$), 5.69 (s, 1H, O*H*), 4.91 (q, *J* = 2.4 Hz, 2H, OC*H*$_2$), 1.88 (t, *J* = 2.4 Hz, 3H, CC*H*$_3$) ppm. **^{13}C NMR** (151 MHz, $CDCl_3$): δ = 169.77 (*C*O$_2$CH$_2$), 148.89 ($C_{arom.}$), 144.99 ($C_{arom.}$), 120.79 ($C_{arom.}$), 120.05 ($C_{arom.}$), 119.31 ($C_{arom.}$), 112.12 ($C_{arom.}$), 84.10 (CH$_2$*C*), 72.51 (*C*CH$_3$), 53.77 (O*C*H$_2$C), 3.71 (C*C*H$_3$) ppm. **MS** (negative ESI-MS, MeOH, acidified): *m/z* (%) = 205.0511 (100, [M-H$^+$], $C_{11}H_9O_4^-$, calcd. 205.0485). **IR** (KBr): $\tilde{\nu}$ (cm^{-1}) = 3749, 3403, 3224, 3041, 2915, 2735, 2327, 2244, 2141, 2050, 1900, 1845, 1681, 1469, 1372, 1291, 1249, 1137, 1067, 970, 900, 843, 805, 738, 687. **Elemental Analysis**: $C_{11}H_{10}O_4 \cdot 1/4\ H_2O$: calcd. C = 62.71 %, H = 5.02 %; found C = 62.90 %, H = 4.75 %.

But-3-yn-2,3-dihydroxybenzoate (3^{3Bu}-H$_2$)

3-Butyn-1-ol (272.86 mg, 3.89 mmol) is converted into the corresponding ester using a modification of the general procedure. 3 equivalents of the alcohol and 6 equivalents of NEt_3 are used and the reaction mixture was refluxed for 3 days. The product is obtained after purification via column chromatography (pentane/ethyl acetate 12:1, R_f = 0.22) as white solid (24 %, 64 mg, 0.31 mmol).

M.p.: 75 °C – 78 °C (last solvent used: DCM). **^{1}H NMR** (600 MHz, $CDCl_3$): δ = 10.75 (s, 1H, O*H*), 7.39 (dd, *J* = 8.0, 1.5 Hz, 1H, $H_{arom.}$), 7.10 (dd, *J* = 8.0, 1.5 Hz, 1H, $H_{arom.}$), 6.79 (t, *J* = 8.0 Hz, 1H, $H_{arom.}$), 5.61 (s, 1H, O*H*), 5.44 (t, *J* = 6.8 Hz, 2H, OCH_2), 2.67 (t, *J* = 6.8 Hz, 2H, CH_2), 2.07-2.05 (m, 1H, C*H*) ppm. **^{13}C NMR** (151 MHz, $CDCl_3$): δ = 169.96 (CO_2CH_2), 148.88 ($C_{arom.}$), 145.01 ($C_{arom.}$), 120.68 ($C_{arom.}$), 119.99 ($C_{arom.}$), 119.30 ($C_{arom.}$), 112.21 ($C_{arom.}$), 79.46 (*C*CH), 70.37 (C*C*H), 63.02 (OCH_2), 18.97 (CH_2) ppm. **MS** (negative ESI-MS, MeOH, acidified): *m/z* (%) = 205.0516 (100, [$M-H^+$], $C_{11}H_9O_4^-$, calcd. 205.0485). **IR** (KBr): $\tilde{\nu}$ (cm^{-1}) = 3944, 3434, 3274, 3109, 2929, 2754, 2462, 2325, 2153, 1922, 1864, 1668, 1462, 1393, 1310, 1247, 1152, 1058, 991, 852, 790, 749, 689. **Elemental Analysis**: $C_{11}H_{10}O_4$: calcd. C = 64.08 %, H = 4.89 %; found C = 63.45 %, H = 4.85 %.

Pent-2-yn-2,3-dihydroxybenzoate (3^{2Pent}-H_2)

2-Pentyn-1-ol (327.48 mg, 3.89 mmol) is converted into the corresponding ester using a modification of the general procedure. 3 equivalents of the alcohol and 6 equivalents of NEt_3 are used and the reaction mixture was refluxed for 3 days. The product is obtained after purification via column chromatography (pentane/ethyl acetate 12:1, R_f = 0.20) as white solid (56 %, 74 mg, 0.34 mmol). **M.p.**: 62 °C – 64 °C (last solvent used: DCM). **^{1}H NMR** (600 MHz, $CDCl_3$): δ = 10.73 (s, 1H, O*H*), 7.41 (dd, *J* = 8.0, 1.6 Hz, 1H, $H_{arom.}$), 7.10 (dd, *J* = 8.0, 1.6 Hz, 1H, $H_{arom.}$), 6.79 (t, *J* = 8.0 Hz, 1H, $H_{arom.}$), 5.63 (s, 1H, O*H*), 4.92 (t, *J* = 2.5 Hz, 2H, OCH_2), 2.24 (t, *J* = 6.8 Hz, 2H, CH_2), 1.14 (t, *J* = 6.8 Hz, 3H, CH_2CH_3) ppm. **^{13}C NMR** (151 MHz, $CDCl_3$): δ = 169.73 (CO_2CH_2), 148.89 ($C_{arom.}$), 145.01 ($C_{arom.}$), 120.80 ($C_{arom.}$), 120.03 ($C_{arom.}$), 119.28 ($C_{arom.}$), 112.17 ($C_{arom.}$), 89.78 (CH_2C), 72.63 (CCH_2), 53.79 (OCH_2C), 13.46 (CH_2), 12.45 (CH_2CH_3) ppm. **MS** (negative and positive ESI-MS, MeOH, acidified): *m/z* (%) = 219.0676 (100, [$M-H^+$], $C_{12}H_{11}O_4^-$, calcd. 219.0642), 243.0635 (100, [$M+Na^+$], $C_{12}H_{12}O_4Na^+$, calcd. 243.0633). **IR** (KBr): $\tilde{\nu}$ (cm^{-1}) = 3397, 3218, 2981, 2941, 2881, 2732, 2444, 2239, 1907, 1679, 1466, 1373, 1290, 1248, 1136, 1064, 1015, 970, 841, 741, 690. **Elemental Analysis**: $C_{12}H_{12}O_4 \cdot H_2O$: calcd. C = 60.50 %, H = 5.92 %, found C = 60.41 %, H = 5.28 %.

Pent-3-yn-2,3-dihydroxybenzoate (3^{3Pent}-H_2)

3-Pentyn-1-ol (327.48 mg, 3.89 mmol) is converted into the corresponding ester using a modification of the general procedure. 3 equivalents of the alcohol and 6 equivalents of NEt_3 are used and the reaction mixture was refluxed for 3 days. The product is obtained after purification via column chromatography (pentane/ethyl acetate 12:1, R_f = 0.18) as colorless oil (24 %, 32 mg, 0.16 mmol). **^{1}H NMR** (600 MHz, $CDCl_3$): δ = 10.83 (s, 1H, O*H*), 7.40 (dd, *J* = 8.0, 1.5 Hz, 1H, $H_{arom.}$), 7.11 (d, *J* = 8.0, 1.5 Hz, 1H, $H_{arom.}$), 6.81 (t, *J* = 8.0 Hz, 1H, $H_{arom.}$), 5.65 (s, 1H, O*H*), 4.40 (t, *J* = 6.9 Hz, 2H, OCH_2), 2.63-2.61 (m, 2H, CH_2), 1.80 (dt, *J* = 9.8, 2.6 Hz, 3H, CCH_3) ppm. **^{13}C NMR** (151 MHz, $CDCl_3$): δ = 170.00 (CO_2CH_2), 148.84 ($C_{arom.}$), 145.00 ($C_{arom.}$), 120.70 ($C_{arom.}$), 119.86 ($C_{arom.}$), 119.23 ($C_{arom.}$), 112.38 ($C_{arom.}$), 77.81 (CH_2C), 74.14 (CCH_3), 63.75 (OCH_2), 19.21 (CH_2), 3.47 (CCH_3) ppm. **MS** (negative ESI-MS, MeOH, acidified): *m/z* (%) = 219.0676 (100, [$M-H^+$], $C_{12}H_{11}O_4^-$, calcd. 219.0642). **IR** (KBr): $\tilde{\nu}$ (cm^{-1}) = 3784, 3468, 3201, 2965, 2921, 2857, 2237, 2054, 1916, 1858, 1673, 1618, 1469, 1393, 1309, 1267, 1152, 1069, 1006, 843, 753, 702, 641, 592, 538, 493. **Elemental Analysis**: $C_{12}H_{12}O_4$: calcd. C = 65.45 %, H = 5.49 %, found C = 65.35 %, H = 5.85 %.

Pent-4-yn-2,3-dihydroxybenzoate (3^{4Pent}-H_2)

4-Pentyn-1-ol (327.48 mg, 3.89 mmol) is converted into the corresponding ester using a modification of the general procedure. 3 equivalents of the alcohol and 6 equivalents of NEt_3 are used and the

reaction mixture was refluxed for 3 days. The product is obtained after purification via column chromatography (pentane/ethyl acetate 12:1, R_f = 0.24) as colorless oil (25 %, 34 mg, 0.15 mmol). **^{1}H NMR** (600 MHz, $CDCl_3$): *δ* = 10.91 (s, 1H, O*H*), 7.37 (dd, *J* = 8.0, 1.5 Hz, 1H, $H_{arom.}$), 7.11 (dd, *J* = 8.0, 1.5 Hz, 1H, $H_{arom.}$), 6.80 (t, *J* = 8.0 Hz, 1H, $H_{arom.}$), 5.67 (s, 1H, O*H*), 4.47 (t, *J* = 6.3 Hz, 2H, OCH_2), 2.41-2.38 (m, 2H, CH_2), 2.05-1.97 (m, 3H, CH_2 & C*H*). **^{13}C NMR** (151 MHz, $CDCl_3$): *δ* = 170.26 (CO_2CH_2), 148.91 ($C_{arom.}$), 145.03 ($C_{arom.}$), 120.51 ($C_{arom.}$), 119.84 ($C_{arom.}$), 119.19 ($C_{arom.}$), 112.41 ($C_{arom.}$), 82.67 (CH_2C), 69.36 (CCH), 63.99 (OCH_2), 27.40 (CH_2), 15.26 (CH_2) ppm. **MS** (negative ESI-MS, MeOH, acidified): *m/z* (%) = 219.0676 (100, [M-H^+], $C_{12}H_{11}O_4^-$, calcd. 219.0642). **IR** (KBr): ṽ (cm^{-1}) = 3917, 3785, 3472, 3295, 2962, 2927, 2849, 2742, 2459, 2118, 1918, 1861, 1672, 1616, 1468, 1393, 1305, 1266, 1152, 1069, 1025, 977, 898, 840, 753, 642, 593, 528, 494, 458. **Elemental Analysis**: $C_{12}H_{12}O_4$: calcd. C = 65.45 %, H = 5.49 %, found C = 65.36 %, H = 5.55 %.

Hex-2-yn-2,3-dihydroxybenzoate (3^{2Hex}-H_2)

2-Hexyn-1-ol (382.06 mg, 3.89 mmol) is converted into the corresponding ester using a modification of the general procedure. 3 equivalents of the alcohol and 6 equivalents of NEt_3 are used and the reaction mixture was refluxed for 3 days. The product is obtained after purification via column chromatography (pentane/ethyl acetate 12:1, R_f = 0.20) as colorless oil (22 %, 66 mg, 0.28 mmol). **^{1}H NMR** (600 MHz, $CDCl_3$): *δ* = 10.73 (s, 1H, O*H*), 7.41 (dd, *J* = 8.0, 1.6 Hz, 1H, $H_{arom.}$), 7.10 (dd, *J* = 8.0, 1.6 Hz, 1H, $H_{arom.}$), 6.79 (t, *J* = 8.0 Hz, 1H, $H_{arom.}$), 5.62 (s, 1H, O*H*), 4.93 (t, *J* = 2.2 Hz, 2H, OCH_2), 2.21-2.17 (m, 2H, CH_2), 1.55-1.52 (m, 2H, CH_2), 0.97 (t, *J* = 7.4 Hz, 3H, CH_2CH_3) ppm. **^{13}C NMR** (151 MHz, $CDCl_3$): *δ* = 169.73 (CO_2CH_2), 148.89 ($C_{arom.}$), 145.00 ($C_{arom.}$), 120.80 ($C_{arom.}$), 120.01 ($C_{arom.}$), 119.28 ($C_{arom.}$), 112.17 ($C_{arom.}$), 88.44 (CCH_2), 73.40 (CH_2C), 53.83 (OCH_2C), 21.78 (CH_2), 20.73 (CH_2), 13.44 (CH_2CH_3) ppm. **MS** (negative ESI-MS, MeOH, acidified): *m/z* (%) = 233.0822 (40, [M-H^+], $C_{13}H_{13}O_4^-$, calcd. 233.0798). **IR** (KBr): ṽ (cm^{-1}) = 3486, 3157, 2960, 2876, 2309, 2239, 2155, 1918, 1673, 1466, 1378, 1293, 1142, 1067, 1028, 976, 894, 836, 749, 700. **Elemental Analysis**: $C_{13}H_{14}O_4$: calcd. C = 66.66 %, H = 6.02 %; found C = 66.45 %, H = 6.12 %.

Hex-3-yn-2,3-dihydroxybenzoate (3^{3Hex}-H_2)

3-Hexyn-1-ol (382.06 mg, 3.89 mmol) is converted into the corresponding ester using a modification of the general procedure. 3 equivalents of the alcohol and 6 equivalents of NEt_3 are used and the reaction mixture was refluxed for 3 days. The product is obtained after purification via column chromatography (pentane/ethyl acetate 12:1, R_f = 0.18) as white solid (22 %, 69 mg, 0.29 mmol). **M.p.**: 70 °C – 72 °C (last solvent used: DCM). **^{1}H NMR** (600 MHz, $CDCl_3$): *δ* = 10.80 (s, 1H, O*H*), 7.39 (dd, *J* = 8.0, 1.5 Hz, 1H, $H_{arom.}$), 7.13 (dd, *J* = 8.0, 1.5 Hz, 1H, $H_{arom.}$), 6.79 (t, *J* = 8.0 Hz, 1H, $H_{arom.}$), 5.61 (s, 1H, O*H*), 4.39 (t, *J* = 6.8 Hz, 2H, OCH_2), 2.63-2.61 (m, 2H, CH_2), 2.20-2.17 (m, 2H, CH_2), 1.09 (t, *J* = 7.5 Hz, 3H, CH_2CH_3) ppm. **^{13}C NMR** (151 MHz, $CDCl_3$): *δ* = 170.00 (CO_2CH_2), 148.83 ($C_{arom.}$), 145.00 ($C_{arom.}$), 120.70 ($C_{arom.}$), 119.84 ($C_{arom.}$), 119.22 ($C_{arom.}$), 112.40 ($C_{arom.}$), 83.93 (CCH_2), 74.27 (CH_2C), 63.77 (OCH_2C), 19.25 (CH_2), 14.05 (CH_2), 12.34 (CH_2CH_3) ppm. **MS** (negative and positive ESI-MS, MeOH, acidified): *m/z* (%) = 233.0822 (100, [M-H^+], $C_{13}H_{13}O_4^-$, calcd. 233.0798), 257.0779 (100, [M+Na^+], $C_{13}H_{14}O_4Na^+$, calcd. 257.0790). **IR** (KBr): ṽ (cm^{-1}) = 3856, 3661, 3471, 3123, 2972, 2918, 2736, 2525, 2326, 2200, 2152, 2049, 2006, 1905, 1659, 1463, 1404, 1315, 1259, 1157, 1066, 1003, 897, 833, 286, 744, 684. **Elemental Analysis**: $C_{13}H_{14}O_4$ · DCM · 2/3 H_2O: calcd. C = 50.77 %, H = 5.28 %; found C = 50.73 %, H = 5.30 %.

Hex-5-yn-2,3-dihydroxybenzoate (3^{5Hex}-H_2)

5-Hexyn-1-ol (382.06 mg, 3.89 mmol) is converted into the corresponding ester using a modification of the general procedure. 3 equivalents of the alcohol and 6 equivalents of NEt_3 are used and the reaction mixture was refluxed for 3 days. The product is obtained after purification via column chromatography (pentane/ethyl acetate 12:1, R_f = 0.30) as colorless oil (7 %, 24 mg, 0.10 mmol). **^{1}H NMR** (600 MHz, $CDCl_3$): δ = 10.95 (s, 1H, O*H*), 7.37 (dd, *J* = 8.0, 1.5 Hz, 1H, $H_{arom.}$), 7.11 (dd, *J* = 8.0, 1.5 Hz, 1H, $H_{arom.}$), 6.80 (t, *J* = 8.0 Hz, 1H, $H_{arom.}$), 5.64 (s, 1H, O*H*), 4.38 (t, *J* = 6.4 Hz, 2H, OCH_2), 2.29 (m, 2H, CH_2), 1.98-1.89 (m, 3H, CH_2 & C*H*), 1.74-1.67 (m, 2H, CH_2) ppm. **MS** (negative ESI-MS, MeOH, acidified): *m/z* (%) = 233.0822 (100, [M-H^+], $C_{13}H_{13}O_4^-$, calcd. 233.0798). **IR** (KBr): ṽ (cm^{-1}) = 3486, 3154, 2963, 2874, 2312, 2239, 2100, 1915, 1673, 1467, 1379, 1297, 1264, 1142, 1067, 1028, 977, 895, 837, 750, 701. **Elemental Analysis**: $C_{13}H_{14}O_4$: calcd. C = 66.66 %, H = 6.02 %; found C = 66.51 %, H = 6.02 %.

Hept-6-yn-2,3-dihydroxybenzoate (3^{6Hept}-H_2)

6-Heptyn-1-ol (1.09 g, 9.73 mmol) is converted into the corresponding ester using a modification of the general procedure. 3 equivalents of the alcohol and 6 equivalents of NEt_3 are used and the reaction mixture was refluxed for 3 days. The product is obtained after purification via column chromatography (pentane/ethyl acetate 12:1, R_f = 0.19) as colorless oil (8 %, 70 mg, 0.28 mmol). **^{1}H NMR** (600 MHz, $CDCl_3$): δ = 10.94 (s, 1H, O*H*), 7.35 (dd, *J* = 8.0, 1.5 Hz, 1H, $H_{arom.}$), 7.10 (dd, *J* = 8.0, 1.5 Hz, 1H, $H_{arom.}$), 6.78 (t, *J* = 8.0 Hz, 1H, $H_{arom.}$), 4.35 (t, *J* = 6.6 Hz, 2H, OCH_2), 2.26-2.15 (m, 2H, CH_2), 1.95-1.91 (m, 1H, C*H*), 1.81-1.77 (m, 2H, CH_2), 1.62-1.46 (m, 4H, 2×CH_2).ppm. **^{13}C NMR** (151 MHz, $CDCl_3$): δ = 170.36 (CO_2CH_2), 148.90 ($C_{arom.}$), 145.02 ($C_{arom.}$), 120.51 ($C_{arom.}$), 119.70 ($C_{arom.}$), 119.13 ($C_{arom.}$), 112.57 ($C_{arom.}$), 84.06 (CH_2C), 68.53 (*C*CH), 65.36 (OCH_2), 28.04 (CH_2), 27.93 (CH_2), 25.01 (CH_2), 18.27 (CH_2) ppm. **MS** (negative and positive ESI-MS, MeOH, acidified): *m/z* (%) = 247.0969 (100, [M-H^+], $C_{14}H_{15}O_4^-$, calcd. 247.0955), 271.0942 (40, [M+Na^+], $C_{14}H_{16}O_4Na^+$, calcd. 271.0946). **IR** (KBr): ṽ (cm^{-1}) = 3853, 3480, 3295, 3123, 2939, 2864, 2663, 2525, 2324, 2113, 1915, 1668, 1466, 1397, 1304, 1264, 1150, 1066, 983, 882, 841, 752. **Elemental Analysis**: $C_{14}H_{16}O_4$ · 1/4 H_2O: calcd. C = 66.52 %, H = 6.58 %; found C = 66.58 %, H = 6.60 %.

Oct-7-yn-2,3-dihydroxybenzoate (3^{7Oct}-H_2)

7-Octyn-1-ol (491 mg, 3.89 mmol) is converted into the corresponding ester using a modification of the general procedure. 3 equivalents of the alcohol and 6 equivalents of NEt_3 are used and the reaction mixture was refluxed for 3 days. The product is obtained after purification via column chromatography (pentane/ethyl acetate 12:1, R_f = 0.24) as colorless oil (28 %, 94 mg, 0.36 mmol). **^{1}H NMR** (600 MHz, $CDCl_3$): δ = 10.98 (s, 1H, O*H*), 7.37 (dd, *J* = 8.0, 1.5 Hz, 1H, $H_{arom.}$), 7.11 (dd, *J* = 8.0, 1.5 Hz, 1H, $H_{arom.}$), 6.80 (t, *J* = 8.0 Hz, 1H, $H_{arom.}$), 5.63 (s, 1H, O*H*), 4.35 (t, *J* = 6.6 Hz, 2H, OCH_2), 3.96 (s, 1H, C*H*), 2.22-2.20 (m, 2H, CH_2), 1.82-1.79 (m, 2H, CH_2), 1.59-1.42 (m, 6H, 3×CH_2) ppm. **^{13}C NMR** (151 MHz, $CDCl_3$): δ = 170.40 (CO_2CH_2), 148.87 ($C_{arom.}$), 145.00 ($C_{arom.}$), 120.51 ($C_{arom.}$), 119.77 ($C_{arom.}$), 119.14 ($C_{arom.}$), 112.59 ($C_{arom.}$), 84.40 (CH_2C), 68.33 (*C*CH), 65.54 (OCH_2), 28.41 (CH_2), 28.29 (CH_2), 28.25 (CH_2), 25.49 (CH_2), 18.32 (CH_2) ppm. **MS** (negative and positive ESI-MS, MeOH, acidified): *m/z* (%) = 261.1138 (100, [M-H^+], $C_{15}H_{17}O_4^-$, calcd. 261.1111), 285.1096 (100, [M+Na^+], $C_{15}H_{18}O_4Na^+$, calcd. 285.1103). **IR** (KBr): ṽ (cm^{-1}) = 3489, 3461, 3274, 3037, 2941, 2857, 2452, 2322, 2156, 2015, 1972, 1921, 1868, 1664, 1460, 1357, 1312, 1264, 1191, 1150, 1068, 990, 906, 837, 784, 751, 659. **Elemental Analysis**: $C_{15}H_{18}O_4$ · 1/2 H_2O: calcd. C = 66.40 %, H = 7.06 %; found C = 66.56 %, H = 6.66 %.

Non-8-yn-2,3-dihydroxybenzoate (3^{8Non}-H_2)

8-Nonyn-1-ol (546 mg, 3.89 mmol) is converted into the corresponding ester using a modification of the general procedure. 3 equivalents of the alcohol and 6 equivalents of NEt_3 are used and the reaction mixture was refluxed for 3 days. The product is obtained after purification via column chromatography (pentane/ethyl acetate 12:1, R_f = 0.21) as yellowish oil (12 %, 46 mg, 0.16 mmol). **^{1}H NMR** (600 MHz, $CDCl_3$): δ = 10.98 (s, 1H, O*H*), 7.37 (dd, *J* = 8.0, 1.5 Hz, 1H, $H_{arom.}$), 7.10 (dd, *J* = 8.0, 1.5 Hz, 1H, $H_{arom.}$), 6.80 (t, *J* = 8.0 Hz, 1H, $H_{arom.}$), 5.63 (s, 1H, O*H*), 4.35 (t, *J* = 6.6 Hz, 2H, OCH_2), 2.21-2.18 (m, 2H, CH_2), 1.94 (t, *J* = 2.7 Hz, 1H, C*H*), 1.79-1.77 (m, 2H, CH_2), 1.53-1.34 (m, 8H, 4×CH_2) ppm. **^{13}C NMR** (151 MHz, $CDCl_3$): δ = 170.40 (CO_2CH_2), 120.51 ($C_{arom.}$), 119.66 ($C_{arom.}$), 119.12 ($C_{arom.}$), 68.20 (C*C*H), 65.60 (OCH_2), 28.68 (CH_2), 28.54 (CH_2), 28.46 (CH_2), 28.32 (CH_2), 25.81 (CH_2), 18.34 (CH_2) ppm. **MS** (negative and positive ESI-MS, MeOH, acidified): *m/z* (%) = 275.1270 (100, [M-H$^+$], $C_{16}H_{19}O_4^-$, calcd. 275.1271), 299.1245 (100, [M+Na$^+$], $C_{16}H_{20}O_4Na^+$, calcd. 299.1261). **IR** (KBr): $\tilde{\nu}$ (cm^{-1}) = 3916, 3473, 3298, 2932, 2858, 2323, 2114, 2000, 1914, 1669, 1614, 1466, 1397, 1303, 1264, 1150, 1067, 990, 910, 842, 752. **Elemental Analysis**: $C_{16}H_{20}O_4$: calcd. C = 69.55 %, H = 7.30 %; found C = 69.40 %, H = 7.71 %.

Dec-9-yn-2,3-dihydroxybenzoate (3^{9Dec}-H_2)

9-Decyn-1-ol (601 mg, 3.89 mmol) is converted into the corresponding ester using a modification of the general procedure. 3 equivalents of the alcohol and 6 equivalents of NEt_3 are used and the reaction mixture was refluxed for 3 days. The product is obtained after purification via column chromatography (pentane/ethyl acetate 12:1, R_f = 0.22) as colorless oil (40 %, 150 mg, 0.52 mmol). **^{1}H NMR** (600 MHz, $CDCl_3$): δ = 10.99 (s, 1H, O*H*), 7.37 (dd, *J* = 8.0, 1.6 Hz, 1H, $H_{arom.}$), 7.10 (dd, *J* = 8.0, 1.6 Hz, 1H, $H_{arom.}$), 6.80 (t, *J* = 8.0 Hz, 1H, $H_{arom.}$), 5.63 (s, 1H, O*H*), 4.34 (t, *J* = 6.6 Hz, 2H, OCH_2), 2.21-2.17 (m, 2H, CH_2), 1.95-1.93 (m, 1H, C*H*), 1.82-1.74 (m, 2H, CH_2), 1.59 -1.29 (m, 8H, 4×CH_2), 0.91-0.79 (m, 2H, CH_2) ppm. **^{13}C NMR** (151 MHz, $CDCl_3$): δ = 170.41 (CO_2CH_2), 148.87 ($C_{arom.}$), 145.00 ($C_{arom.}$), 120.52 ($C_{arom.}$), 119.65 ($C_{arom.}$), 119.12 ($C_{arom.}$), 112.62 ($C_{arom.}$), 84.67 (CH_2C), 68.14 (C*C*H), 65.66 (OCH_2), 29.07 (CH_2), 28.94 (CH_2), 28.60 (CH_2), 28.49 (CH_2), 28.39 (CH_2), 25.87 (CH_2), 18.36 (CH_2) ppm. **MS** (negative and positive ESI-MS, MeOH, acidified): *m/z* (%) = 289.1443 (100, [M-H$^+$], $C_{17}H_{21}O_4^-$, calcd. 289.1424), 313.1423 (10, [M+Na$^+$], $C_{17}H_{22}O_4Na^+$, calcd. 313.1508). **IR** (KBr): $\tilde{\nu}$ (cm^{-1}) = 3922, 3301, 2931, 2857, 2115, 1675, 1616, 1466, 1397, 1304, 1154, 1068, 998, 846, 753, 721, 635, 497. **Elemental Analysis**: $C_{17}H_{22}O_4$ · 1/2 pentane: calcd. C = 71.75 %, H = 8.65 %, found C = 71.41 %, H = 8.64 %.

Undec-10-yn-2,3-dihydroxybenzoate ($3^{10Undec}$-H_2)

10-Undecyn-1-ol (655 mg, 3.89 mmol) is converted into the corresponding ester using a modification of the general procedure. 3 equivalents of the alcohol and 6 equivalents of NEt_3 are used and the reaction mixture was refluxed for 3 days. The product is obtained after purification via column chromatography (pentane/ethyl acetate 12:1, R_f = 0.20) as colourless oil (20 %, 80 mg, 0.26 mmol). **^{1}H NMR** (600 MHz, $CDCl_3$): δ = 10.98 (s, 1H, O*H*), 7.37 (dd, *J* = 8.0, 1.5 Hz, 1H, $H_{arom.}$), 7.10 (dd, *J* = 8.0, 1.5 Hz, 1H, $H_{arom.}$), 6.80 (t, *J* = 8.0 Hz, 1H, $H_{arom.}$), 5.64 (s, 1H, O*H*), 4.34 (t, *J* = 6.7 Hz, 2H, OCH_2), 2.19-2.17 (m, 2H, CH_2), 1.94 (t, *J* = 2.6 Hz, 1H, C*H*), 1.79-1.75 (m, 2H, CH_2), 1.58-1.48 (m, 4H, 2×CH_2), 1.48-1.27 (m, 8H, 4×CH_2) ppm. **^{13}C NMR** (150 MHz, $CDCl_3$): δ = 170.42 (CO_2CH_2), 148.87 ($C_{arom.}$), 145.00 ($C_{arom.}$), 120.52 ($C_{arom.}$), 119.65 ($C_{arom.}$), 119.12 ($C_{arom.}$), 112.63 ($C_{arom.}$), 84.74 (CH_2C), 68.10 (C*C*H), 65.69 (OCH_2), 29.72 (CH_2), 29.32 (CH_2), 28.99 (CH_2), 28.68 (CH_2), 28.50 (CH_2), 28.43 (CH_2), 25.90 (CH_2), 18.38

(CH_2) ppm. **MS** (negative and positive ESI-MS, MeOH, acidified): *m/z* (%) = 303.1596 (30, [M-H$^+$], $C_{18}H_{23}O_4^-$, calcd. 303.1581), 327.1577 (40, [M+Na$^+$], $C_{18}H_{24}O_4Na^+$, calcd. 327.1572). **IR** (KBr): ṽ (cm^{-1}) = 3916, 3785, 3662, 3483, 3301, 3131, 2930, 2856, 2459, 2318, 2116, 1914, 1856, 1671, 1614, 1469, 1397, 1307, 1266, 1153, 1068, 992, 900, 843, 754, 717, 635, 594, 529, 494. **Elemental Analysis**: $C_{18}H_{24}O_4$: calcd. C = 71.03 %, H = 7.95 %, found C = 70.61 %, H = 7.92 %.

Complexes Li[Li$_3$(3$_3$Ti)$_2$]

Li[Li$_3$($3^{Prop}{}_3$Ti)$_2$]

Ligand **3^{Prop}**-H_2 (50 mg, 0.26 mmol) is converted into the corresponding complex in methanol (50 mL). The product is obtained after removal of the solvent under reduced pressure as red solid (quantitative). **^{1}H NMR** (600 MHz, MeOH-d_4): Dimer (major component): δ = 7.14 (dd, *J* = 7.6, 1.7 Hz, 1H, H$_{arom.}$), 6.69-6.57 (m, 2H, H$_{arom.}$), 4.19 (dd, *J* = 15.7, 2.4 Hz, 1H, OC*H*$_2$), 3.62 (dd, *J* = 15.7, 2.4 Hz, 1H, OC*H*$_2$), 2.79 (t, *J* = 2.4 Hz, 1H, CC*H*) ppm. Monomer (minor component): δ = 7.35 (dd, *J* = 7.9, 1.5 Hz, 1H, H$_{arom.}$), 7.03 (dd, *J* = 7.9, 1.5 Hz, 1H, H$_{arom.}$), 6.78 (t, *J* = 7.9 Hz, 1H, H$_{arom.}$), 4.78 (d, *J* = 3.2 Hz, 2H, OC*H*$_2$), 3.07 (t, *J* = 2.4 Hz, 1H, CC*H*) ppm. **^{1}H NMR** (400 MHz, DMSO-d_6): Dimer (minor component): δ = 6.94 (dd, *J* = 7.6, 1.7 Hz, 1H, H$_{arom.}$), 6.55 (t, *J* = 7.6 Hz, 1H, H$_{arom.}$), 6.47 (dd, *J* = 7.6, 1.7 Hz, 1H, H$_{arom.}$), 4.02 (dd, *J* = 15.0, 2.4 Hz, 1H, OC*H*$_2$), 3.62 (dd, *J* = 15.0, 2.4 Hz, 1H, OC*H*$_2$), 2.64 (t, *J* = 2.4 Hz, 1H, CC*H*) ppm. Monomer (major component): δ = 6.75 (dd, *J* = 7.8, 1.6 Hz, 1H, H$_{arom.}$), 6.25 (t, *J* = 7.8 Hz, 1H, H$_{arom.}$), 6.12 (dd, *J* = 7.8, 1.6 Hz, 1H, H$_{arom.}$), 4.70 (d, *J* = 2.5 Hz, 2H, OC*H*$_2$), 3.37 (t, *J* = 2.4 Hz, 1H, CC*H*) ppm. **MS** (negative and positive ESI-MS, MeOH): *m/z* (%) = 1257.1036 (30, [M$_D$-Li$^+$], $C_{60}H_{36}O_{24}Li_3Ti_2^-$, calcd. 1257.1036), 1287.1161 (100, [M$_D$+Na$^+$], $C_{60}H_{36}O_{24}Li_4Ti_2Na^+$, calcd. 1287.1195), 655.0512 (97, [M$_M$+Na$^+$], $C_{30}H_{18}O_{12}Li_2TiNa^+$, calcd. 655.0498). **IR** (KBr): ṽ (cm^{-1}) = 3937, 3750, 3382, 3280, 3068, 2851, 2599, 2413, 2284, 2159, 2128, 2044, 2013, 1981, 1900, 1678, 1594, 1561, 1441, 1375, 1290, 1250, 1213, 1186, 1151, 1065, 1021, 948, 899, 850, 798, 742, 679. **Elemental analysis:** $C_{60}H_{36}O_{24}Li_4Ti_2 \cdot 5\ H_2O$: calcd. C = 53.21 %, H = 3.42 %; found C = 53.43 %, H = 3.35 %.

Li[Li$_3$($3^{2Bu}{}_3$Ti)$_2$]

Ligand **3^{2Bu}**-H_2 (30 mg, 0.14 mmol) is converted into the corresponding complex in methanol (30 mL). The product is obtained after removal of the solvent under reduced pressure as red solid (quantitative). **^{1}H NMR** (600 MHz, MeOH-d_4): Dimer (major component): δ = 7.12 (dd, *J* = 7.8, 1.6 Hz, 1H, H$_{arom.}$), 6.68-6.55 (m, 2H, H$_{arom.}$), 4.12 (dd, *J* = 15.4, 2.4 Hz, 1H, OC*H*$_2$), 3.56 (dd, *J* = 15.4, 2.4 Hz, 1H, OC*H*$_2$), 1.76 (t, *J* = 2.4 Hz, 3H, CC*H*$_3$) ppm. Monomer (minor component): δ = 7.35 (dd, *J* = 7.8, 1.6 Hz, 1H, H$_{arom.}$), 7.03 (dd, *J* = 7.8, 1.6 Hz, 1H, H$_{arom.}$), 6.76 (t, *J* = 7.8 Hz, 1H, H$_{arom.}$), 4.92 (d, *J* = 6.5 Hz, OC*H*$_2$), 1.86 (t, *J* = 2.4 Hz, 3H, CC*H*$_3$) ppm. **^{1}H NMR** (400 MHz, DMSO-d_6): Dimer (minor component): δ = 6.93 (dd, *J* = 7.9, 1.5 Hz, 1H, H$_{arom.}$), 6.54 (t, *J* = 7.9 Hz, 1H, H$_{arom.}$), 6.46 (dd, *J* = 7.9, 1.5 Hz, 1H, H$_{arom.}$), 4.01-3.99 (m, 1H, OC*H*$_2$), 3.48-3.46 (m, 1H, OC*H*$_2$), 1.72 (t, *J* = 2.4 Hz, 3H, CC*H*$_3$) ppm. Monomer (major component): δ = 6.75 (dd, *J* = 7.9, 1.5 Hz, 1H, H$_{arom.}$), 6.25 (t, *J* = 7.9 Hz, 1H, H$_{arom.}$), 6.11 (dd, *J* = 7.9, 1.5 Hz, 1H, H$_{arom.}$), 4.64 (d, *J* = 6.6 Hz, 2H, OC*H*$_2$), 1.78 (t, *J* = 2.4 Hz, 3H, CC*H*$_3$) ppm. **MS** (negative and positive ESI-MS, MeOH): *m/z* (%) = 1341.1978 (100, [M$_D$-Li$^+$], $C_{66}H_{48}O_{24}Li_3Ti_2^-$, calcd. 1341.1975), 1355.2304 (50, [M$_D$+Li$^+$], $C_{66}H_{48}O_{24}Li_5Ti_2^+$, calcd. 1355.2294). **IR** (KBr): ṽ (cm^{-1}) = 3851, 3610, 3362, 3074, 2920, 2692, 2498, 2311, 2243, 2118, 2040, 1987, 1910, 1678, 1593, 1557, 1444, 1376, 1290, 1252, 1215, 1156, 1066, 992, 901, 834, 747, 680. **Elemental analysis:** $C_{66}H_{48}O_{24}Li_4Ti_2 \cdot 4\ H_2O$: calcd. C = 55.80 %, H = 3.97 %; found C = 55.88 %, H = 3.23 %.

Li[Li$_3$(3^{3Bu}$_3$Ti)$_2$]

Ligand **3^{3Bu}**-H$_2$ (30 mg, 0.14 mmol) is converted into the corresponding complex in methanol (30 mL). The product is obtained after removal of the solvent under reduced pressure as red solid (quantitative). **^{1}H NMR** (600 MHz, MeOH-d_4): Dimer (major component): δ = 7.17 (dd, J = 6.6, 2.9 Hz, 1H, H$_{arom.}$), 6.62-6.59 (m, 2H, H$_{arom.}$), 3.66-3.64 (m, 1H, OCH_2), 3.15-3.13 (m, 1H, OCH_2), 2.35-2.23 (m, 2H, CH_2C), 2.21 (t, J = 2.6 Hz, 1H, CCH) ppm. Monomer (minor component): δ = 7.35 (dd, J = 6.6, 2.9 Hz, 1H, H$_{arom.}$), 7.02 (dd, J = 6.6, 2.9 Hz, 1H, H$_{arom.}$), 6.74 (t, J = 6.6 Hz, 1H, H$_{arom.}$), 4.44 (t, J = 4.4 Hz, 2H, OCH_2), 2.70-2.66 (m, 2H, CH_2C), 2.36 (t, J = 2.4 Hz, 1H, CCH) ppm. **^{1}H NMR** (400 MHz, DMSO-d_6): Dimer (minor component): δ = 6.98 (dd, J = 7.9, 1.6 Hz, 1H, H$_{arom.}$), 6.51 (t, J = 7.9 Hz, 1H, H$_{arom.}$), 6.40 (dd, J = 7.9, 1.6 Hz, 1H, H$_{arom.}$), 3.55-3.51 (m, 1H, OCH_2), 3.03-2.93 (m, 1H, OCH_2), 2.73 (t, J = 2.6 Hz, 1H, CCH), 2.25-2.21 (m, 2H, CH_2C) ppm. Monomer (major component): δ = 6.76 (dd, J = 7.9, 1.6 Hz, 1H, H$_{arom.}$), 6.24 (t, J = 7.9 Hz, 1H, H$_{arom.}$), 6.10 (dd, J = 7.9, 1.6 Hz, 1H, H$_{arom.}$), 4.11 (t, J = 6.9 Hz, 2H, OCH_2), 2.79 (t, J = 2.7 Hz, 1H, CCH), 2.57-2.47 (m, 2H, CH_2C) ppm. **MS** (negative ESI-MS, MeOH): m/z (%) = 1341.2009 (100, [M$_D$-Li$^+$], $C_{66}H_{48}O_{24}Li_3Ti_2^-$, calcd. 1341.1975). **IR** (KBr): ṽ (cm^{-1}) = 3900, 3619, 3282, 3067, 2964, 2912, 2653, 2318, 2243, 2205, 2081, 2036, 1990, 1962, 1941, 1903, 1675, 1594, 1561, 1444, 1389, 1343, 1298, 1253, 1214, 1154, 1066, 1011, 979, 855, 805, 746, 684. **Elemental analysis:** $C_{66}H_{48}O_{24}Li_4Ti_2 \cdot 10\ H_2O$: calcd. C = 51.86 %, H = 4.48 %; found C = 51.71 %, H = 4.10 %.

Li[Li$_3$(3^{2Pent}$_3$Ti)$_2$]

Ligand **3^{2Pent}**-H$_2$ (50 mg, 0.23 mmol) is converted into the corresponding complex in methanol (50 mL). The product is obtained after removal of the solvent under reduced pressure as red solid (quantitative). **^{1}H NMR** (600 MHz, MeOH-d_4): Dimer (major component): δ = 7.12 (dd, J = 7.9, 1.5 Hz, 1H, H$_{arom.}$), 6.66-6.55 (m, 2H, H$_{arom.}$), 4.12 (d, J = 15.0 Hz, 1H, OCH_2C), 3.59 (d, J = 15.0 Hz, 1H, OCH_2C), 2.16-2.12 (m, 2H, CH_2), 1.07 (t, J = 7.4 Hz, 3H, CH$_2$CH_3) ppm. Monomer (minor component): δ = 7.36 (dd, J = 7.8, 1.5 Hz, 1H, H$_{arom.}$), 7.02 (dd, J = 7.8, 1.5 Hz, 1H, H$_{arom.}$), 6.78 (t, J = 7.8 Hz, 1H, H$_{arom.}$), 4.86 (s, 2H, OCH_2C), 2.21 (m, 2H, CH_2), 1.18 (m, 3H, CH$_2$CH_3) ppm. **^{1}H NMR** (400 MHz, DMSO-d_6): Dimer (minor component): δ = 6.96 (dd, J = 8.1, 1.6 Hz, 1H, H$_{arom.}$), 6.56 (t, J = 8.1 Hz, 1H, H$_{arom.}$), 6.49 (dd, J = 8.1, 1.6 Hz, 1H, H$_{arom.}$), 4.03 (d, J = 14.9 Hz, 1H, OCH_2C), 3.57 (d, J = 14.9 Hz, 1H, OCH_2C), 2.13-2.11 (m, 2H, CH_2), 0.96 (t, J = 7.2 Hz, 3H, CH$_2$CH_3) ppm. Monomer (major component): δ = 6.78 (dd, J = 8.1, 1.6 Hz, 1H, H$_{arom.}$), 6.27 (t, J = 8.1 Hz, 1H, H$_{arom.}$), 6.14 (dd, J = 8.1, 1.6 Hz, 1H, H$_{arom.}$), 4.71 (s, 2H, OCH_2C), 2.21-2.18 (m, 2H, CH_2), 1.03 (t, J = 7.2 Hz, 3H, CH$_2$CH_3) ppm. **MS** (negative and positive ESI-MS, MeOH): m/z (%) = 1425.2992 (100, [M$_D$-Li$^+$], $C_{72}H_{60}O_{24}Li_3Ti_2^-$, calcd. 1425.2914), 1439.3245 (20, [M$_D$+Li$^+$], $C_{72}H_{60}O_{24}Li_5Ti_2^+$, calcd. 1439.3233). **IR** (KBr): ṽ (cm^{-1}) = 3395, 2976, 2674, 2239, 2079, 1993, 1902, 1745, 1676, 1592, 1559, 1443, 1374, 1289, 1252, 1213, 1149, 1064, 988, 898, 817, 742, 679. **Elemental analysis**: $C_{72}H_{60}Li_4O_{24}Ti_2 \cdot 4\ H_2O$: calcd. C = 57.47 %, H = 4.56 %; found C = 57.87 %, H = 4.34 %.

Li[Li$_3$(3^{3Pent}$_3$Ti)$_2$]

Ligand **3^{3Pent}**-H$_2$ (25 mg, 0.12 mmol) is converted into the corresponding complex in methanol (25 mL). The product is obtained after removal of the solvent under reduced pressure as red solid (quantitative). **^{1}H NMR** (600 MHz, MeOH-d_4): Only dimer: δ = 7.13 (dd, J = 7.8, 1.6 Hz, 1H, H$_{arom.}$), 6.60-6.56 (m, 2H, H$_{arom.}$), 3.61-3.54 (m, 1H, OCH_2), 3.10-3.06 (m, 1H, OCH_2), 2.19-2.15 (m, 2H, CH_2), 1.69 (t, J = 2.5 Hz, 3H, CCH_3) ppm. **^{1}H NMR** (400 MHz, DMSO-d_6): Dimer (minor component): δ = 6.96 (dd, J = 7.9, 1.6 Hz, 1H, H$_{arom.}$), 6.51 (t, J = 7.9 Hz, 1H, H$_{arom.}$), 6.40 (dd, J = 7.9, 1.6 Hz, 1H, H$_{arom.}$), 3.53-3.42 (m, 1H, OCH_2), 2.97-2.94 (m, 1H, OCH_2), 2.17-2.13 (m, 2H, CH_2), 1.66 (t, J = 2.7 Hz, 3H, CCH_3)

ppm. Monomer (major component): δ = 6.74 (dd, J = 7.8, 1.6 Hz, 1H, $H_{arom.}$), 6.23 (t, J = 7.8 Hz, 1H, $H_{arom.}$), 6.09 (dd, J = 7.8, 1.6 Hz, 1H, $H_{arom.}$), 4.07 (t, J = 7.1 Hz, 2H, OCH_2), 2.45-2.43 (m, 2H, CH_2), 1.70 (t, J = 2.7 Hz, 3H, CCH_3) ppm. **MS** (negative and positive ESI-MS, MeOH): m/z (%) = 1425.2979 (100, $[M_D\text{-}Li^+]$, $C_{72}H_{60}O_{24}Li_3Ti_2^-$, calcd. 1425.2914), 1439.3176 (80, $[M_D\text{+}Li^+]$, $C_{72}H_{60}O_{24}Li_5Ti_2^+$, calcd. 1439.3233). **IR** (KBr): ṽ (cm^{-1}) = 3587, 3365, 3069, 2962, 2917, 2853, 2663, 2319, 2243, 2199, 2077, 2034, 1981, 1948, 1904, 1679, 1593, 1561, 1444, 1390, 1344, 1298, 1253, 1215, 1154, 1064, 1005, 951, 892, 857, 809, 743, 684. **Elemental analysis**: $C_{72}H_{60}Li_4O_{24}Ti_2 \cdot 6\ H_2O$: calcd. C = 56.13 %, H = 4.71 %; found: C = 56.23 %, H = 4.50 %.

$Li[Li_3(3^{4Pent}{}_3Ti)_2]$

Ligand **3^{4Pent}**-H_2 (50 mg, 0.23 mmol) is converted into the corresponding complex in methanol (50 mL). The product is obtained after removal of the solvent under reduced pressure as red solid (quantitative). **^{1}H NMR** (400 MHz, MeOH-d_4): Only dimer: δ = 7.11 (dd, J = 7.8, 1.5 Hz, 1H, $H_{arom.}$), 6.62-6.54 (m, 2H, $H_{arom.}$), 3.70-3.64 (m, 1H, OCH_2), 3.19-3.08 (m, 1H, OCH_2), 2.16-2.06 (m, 3H, CH_2 & CH), 1.68-1.51 (m, 2H, CH_2) ppm. **^{1}H NMR** (400 MHz, DMSO-d_6): Dimer (major component): δ = 6.98 (dd, J = 8.1, 1.7 Hz, 1H, $H_{arom.}$), 6.5 (t, J = 8.1 Hz, 1H, $H_{arom.}$), 6.41 (dd, J = 8.1, 1.7 Hz, 1H, $H_{arom.}$), 3.53-3.50 (m, 1H, OCH_2), 2.97-2.94 (m, 1H, OCH_2), 2.10-2.03 (m, 3H, CH_2 & CH), 1.58-1.44 (m, 2H, CH_2) ppm. Monomer (minor component): δ = 6.74 (dd, J = 8.1, 1.7 Hz, 1H, $H_{arom.}$), 6.23 (t, J = 8.1 Hz, 1H, $H_{arom.}$), 6.11 (dd, J = 8.1, 1.7 Hz, 1H, $H_{arom.}$), 4.10 (t, J = 6.3 Hz, 2H, OCH_2), 2.70 (t, J = 2.6 Hz, 1H, CH), 2.27-2.23 (m, 2H, CH_2), 1.79-1.75 (m, 2H, CH_2) ppm. **MS** (negative and positive ESI-MS, MeOH): m/z (%) = 1425.2979 (100, $[M_D\text{-}Li^+]$, $C_{72}H_{60}O_{24}Li_3Ti_2^-$, calcd. 1425.2914), 1439.3176 (10, $[M_D\text{+}Li^+]$, $C_{72}H_{60}O_{24}Li_5Ti_2^+$, calcd. 1439.3233). **IR** (KBr): ṽ (cm^{-1}) = 3868, 3292, 2949, 2683, 2507, 2323, 2226, 2076, 1900, 1674, 1594, 1553, 1444, 1348, 1291, 1251, 1212, 1067, 1031, 989, 897, 849, 803, 740, 681. **Elemental analysis:** $C_{72}H_{60}Li_4O_{24}Ti_2 \cdot 4/3\ H_2O$: calcd. C = 59.36 %, H = 4.34 %; found C = 59.07 %, H = 3.86 %.

$Li[Li_3(3^{2Hex}{}_3Ti)_2]$

Ligand **3^{2Hex}**-H_2 (30 mg, 0.13 mmol) is converted into the corresponding complex in methanol (30 mL). The product is obtained after removal of the solvent under reduced pressure as red solid (quantitative). **^{1}H NMR** (600 MHz, MeOH-d_4): Dimer (major component): δ = 7.13 (dd, J = 7.4, 2.3 Hz, 1H, $H_{arom.}$), 6.66-6.58 (m, 2H, $H_{arom.}$), 4.12 (d, J = 15.4 Hz, 1H, OCH_2), 3.59 (d, J = 15.4 Hz, 1H, OCH_2), 2.13-2.10 (m, 2H, CCH_2), 1.45-1.43 (m, 2H, CH_2), 0.92 (t, J = 2.7 Hz, 3H, CH_2CH_3) ppm. Monomer (minor component): δ = 7.34 (dd, J = 7.7, 1.9 Hz, 1H, $H_{arom.}$), 7.02 (dd, J = 7.7, 1.9 Hz, 1H, $H_{arom.}$), 6.76 (t, J = 7.7 Hz, 1H, $H_{arom.}$), 4.61 (s, 2H, OCH_2), 2.23-2.17 (m, 2H, CCH_2), 1.57-1.51 (m, 2H, CH_2), 0.98 (t, J = 2.8 Hz, 3H, CH_2CH_3) ppm. **^{1}H NMR** (400 MHz, DMSO-d_6): Dimer (minor component): δ = 6.98 (dd, J = 7.9, 1.6 Hz, 1H, $H_{arom.}$), 6.55 (t, J = 7.9 Hz, 1H, $H_{arom.}$), 6.47 (dd, J = 7.9, 1.6 Hz, 1H, $H_{arom.}$), 4.00 (d, J = 15.2 Hz, 1H, OCH_2), 3.50 (d, J = 15.2 Hz, 1H, OCH_2), 2.08-2.06 (m, 2H, CCH_2), 1.33-1.31 (m, 2H, CH_2), 0.81 (t, J = 2.7 Hz, 3H, CH_2CH_3) ppm. Monomer (major component): δ = 6.75 (dd, J = 7.9, 1.6 Hz, 1H, $H_{arom.}$), 6.25 (t, J = 7.9 Hz, 1H, $H_{arom.}$), 6.11 (dd, J = 7.9, 1.6 Hz, 1H, $H_{arom.}$), 4.69 (s, 2H, OCH_2), 2.18-2.16 (m, 2H, CCH_2), 1.43-1.41 (m, 2H, CH_2), 0.89 (t, J = 2.8 Hz, 3H, CH_2CH_3) ppm. **MS** (negative and positive ESI-MS, MeOH, acidified): m/z (%) = 1509.3853 (100, $[M_D\text{-}Li^+]$, $C_{78}H_{72}O_{24}Li_3Ti_2^-$, calcd. 1509.3853), 751.1834 (98, $[M_M\text{-}Li^+]$, $C_{39}H_{36}O_{12}LiTi^-$, calcd. 751.1846), 1523.4141 (98, $[M_D\text{+}Li^+]$, $C_{78}H_{72}O_{24}Li_5Ti_2^+$, calcd. 1523.4172), 765.2140 (100, $[M_M\text{+}Li^+]$, $C_{39}H_{36}O_{12}Li_3Ti^+$, calcd. 765.2166). **IR** (KBr): ṽ (cm^{-1}) = 3457, 3013, 2968, 2872, 2450, 2243, 2106, 1997, 1960, 1740, 1679, 1593, 1557, 1443, 1370, 1290,

1216, 1153, 1066, 991, 900, 850, 795, 742, 681. **Elemental analysis:** $C_{78}H_{72}O_{24}Li_4Ti_2 \cdot 4\ H_2O$: calcd. C = 58.96 %, H = 5.08 %; found C = 58.40 %, H = 4.92 %.

Li[Li$_3$(3^{3Hex}$_3$Ti)$_2$]

Ligand **3^{3Hex}**-H$_2$ (30 mg, 0.13 mmol) is converted into the corresponding complex in methanol (30 mL). The product is obtained after removal of the solvent under reduced pressure as red solid (quantitative). **^{1}H NMR** (600 MHz, MeOH-d_4): Dimer (major component): δ = 7.17 (dd, J = 7.8, 1.5 Hz, 1H, $H_{arom.}$), 6.63-6.57 (m, 2H, $H_{arom.}$), 3.61-3.57 (m, 1H, OCH_2), 3.10-3.07 (m, 1H, OCH_2), 2.23-2.19 (m, 2H, CH_2C) ppm. Monomer (minor component): δ = 7.38 (dd, J = 7.6, 1.4 Hz, 1H, $H_{arom.}$), 7.02 (dd, J = 7.6, 1.4 Hz, 1H, $H_{arom.}$), 6.76 (t, J = 7.6 Hz, 1H, $H_{arom.}$), 4.39 (t, J = 6.8 Hz, 2H, OCH_2), 2.63-2.61 (m, 2H, CH_2C) ppm. Signals not listed are overlapping and cannot be assigned. **^{1}H NMR** (400 MHz, DMSO-d_6): Dimer (major component): δ = 6.98 (dd, J = 7.9, 1.6 Hz, 1H, $H_{arom.}$), 6.50 (t, J = 7.9 Hz, 1H, $H_{arom.}$), 6.39 (dd, J = 7.9, 1.6 Hz, 1H, $H_{arom.}$), 3.49-3.47 (m, 1H, OCH_2), 2.88-2.86 (m, 1H, OCH_2) ppm. Monomer (minor component): δ = 6.74 (dd, J = 7.9, 1.6 Hz, 1H, $H_{arom.}$), 6.23 (t, J = 7.9 Hz, 1H, $H_{arom.}$), 6.10 (dd, J = 7.9, 1.6 Hz, 1H, $H_{arom.}$), 4.07 (t, J = 7.1 Hz, 2H, OCH_2) ppm. Signals not listed are overlapping and cannot be assigned. **MS** (negative and positive ESI-MS, MeOH, acidified): m/z (%) = 1509.3834 (100, [M_D-Li$^+$], $C_{78}H_{72}O_{24}Li_3Ti_2^-$, calcd. 1509.3853), 1523.4172 (10, [M_D+Li$^+$], $C_{78}H_{72}O_{24}Li_5Ti_2^+$, calcd. 1523.4173). **IR** (KBr): $\tilde{\nu}$ (cm^{-1}) = 3861, 3593, 3363, 3069, 2973, 2917, 2693, 2513, 2322, 2195, 2156, 2014, 1966, 1929, 1677, 1594, 1561, 1443, 1389, 1341, 1297, 1253, 1214, 1153, 1065, 1012, 891, 856, 807, 743, 683. **Elemental analysis:** $C_{78}H_{72}O_{24}Li_4Ti_2 \cdot 3\ H_2O$: calcd. C = 59.64 %, H = 5.00 %; found C = 59.39 %, H = 5.04 %.

Li[Li$_3$(3^{5Hex}$_3$Ti)$_2$]

Ligand **3^{5Hex}**-H$_2$ (20 mg, 0.08 mmol) is converted into the corresponding complex in methanol (25 mL). The product is obtained after removal of the solvent under reduced pressure as red solid (quantitative). **^{1}H NMR** (600 MHz, MeOH-d_4): Only dimer: δ = 7.16 (dd, J = 7.5, 2.3 Hz, 1H, $H_{arom.}$), 6.63-6.57 (m, 2H, $H_{arom.}$), 3.61-3.59 (m, 1H, OCH_2), 3.04-2.97 (m, 1H, OCH_2), 2.15 (t, J = 7.0 Hz, 2H, CH_2), 1.88 (s, 1H, CH), 1.58-1.46 (m, 2H, CH_2), 1.45-1.39 (m, 2H, CH_2) ppm. **^{1}H NMR** (400 MHz, DMSO-d_6): Dimer (major component): δ = 6.98 (dd, J = 7.9, 1.6 Hz, 1H, $H_{arom.}$), 6.50 (t, J = 7.9 Hz, 1H, $H_{arom.}$), 6.40 (dd, J = 7.9, 1.6 Hz, 1H, $H_{arom.}$), 3.49-3.47 (m, 1H, OCH_2), 2.87-2.84 (m, 1H, OCH_2) ppm. Monomer (minor component): δ = 6.72 (dd, J = 7.9, 1.6 Hz, 1H, $H_{arom.}$), 6.22 (t, J = 7.9 Hz, 1H, $H_{arom.}$), 6.10 (dd, J = 7.9, 1.6 Hz, 1H, $H_{arom.}$), 4.05 (t, J = 6.4 Hz, 2H, OCH_2) ppm. Signals not listed are overlapping and cannot be assigned. **MS** (negative ESI-MS, MeOH, acidified): m/z (%) = 1509.3892 (100, [M_D-Li$^+$], $C_{78}H_{72}O_{24}Li_3Ti_2^-$, calcd. 1509.3853). **IR** (KBr): $\tilde{\nu}$ (cm^{-1}) = 3292, 2925, 2858, 2686, 2293, 2174, 2114, 2046, 1892, 1675, 1592, 1446, 1392, 1347, 1297, 1254, 1217, 1154, 1063, 1018, 935, 893, 858, 803, 743, 681. **Elemental analysis:** $C_{78}H_{72}O_{24}Li_4Ti_2 \cdot 5\ H_2O$: calcd. C = 58.30 %, H = 5.14 %; found C = 58.40 %, H = 4.86 %.

Li[Li$_3$(3^{6Hept}$_3$Ti)$_2$]

Ligand **3^{6Hept}**-H$_2$ (40 mg, 0.16 mmol) is converted into the corresponding complex in methanol (50 mL). The product is obtained after removal of the solvent under reduced pressure as red solid (quantitative). **^{1}H NMR** (600 MHz, MeOH-d_4): Only dimer: δ = 7.12 (dd, J = 7.8, 1.5 Hz, 1H, $H_{arom.}$), 6.61-6.52 (m, 2H, $H_{arom.}$), 3.60-3.56 (m, 1H, OCH_2), 3.05-2.98 (m, 1H, OCH_2), 2.21-2.07 (m, 2H, CH_2), 1.66-1.63 (m, 1H, CH), 1.55-1.32 (m, 6H, 3×CH_2) ppm. **^{1}H NMR** (400 MHz, DMSO-d_6): Dimer (major

component): δ = 6.95 (dd, J = 8.0, 1.5 Hz, 1H, $H_{arom.}$), 6.49 (t, J = 8.0 Hz, 1H, $H_{arom.}$), 6.39 (dd, J = 8.0, 1.5 Hz, 1H, $H_{arom.}$), 3.47-3.44 (m, 1H, OCH_2), 2.86-2.83 (m, 1H, OCH_2) ppm. Monomer (minor component): δ = 6.73 (dd, J = 8.0, 1.5 Hz, 1H, $H_{arom.}$), 6.22 (t, J = 8.0 Hz, 1H, $H_{arom.}$), 6.08 (dd, J = 8.0, 1.5 Hz, 1H, $H_{arom.}$), 4.03 (t, J = 6.5 Hz, 2H, OCH_2) ppm. Signals not listed are overlapping and cannot be assigned. **MS** (negative ESI-MS, MeOH): *m/z* (%) = 1593.4873 (100, $[M_D\text{-}Li^+]$, $C_{84}H_{84}O_{24}Li_3Ti_2^-$, calcd. 1593.4791). **IR** (KBr): ṽ (cm^{-1}) = 3292, 3068, 2936, 2862, 2680, 2116, 1898, 1747, 1673, 1593, 1558, 1444, 1390, 1346, 1293, 1251, 1207, 1063, 999, 853, 804, 793, 679. **Elemental analysis:** $C_{84}H_{84}Li_4O_{24}Ti_2 \cdot 4\ H_2O$: calcd. C = 60.30 %, H = 5.54 %; found C = 60.02 %, H = 5.44 %.

$Li[Li_3(3^{7Oct}{}_3Ti)_2]$

Ligand **3^{7Oct}**-H_2 (50 mg, 0.19 mmol) is converted into the corresponding complex in methanol (50 mL). The product is obtained after removal of the solvent under reduced pressure as red solid (quantitative). **^{1}H NMR** (600 MHz, MeOH-d_4): Dimer (major component): δ = 7.09 (dd, J = 8.0, 2.8 Hz, 1H, $H_{arom.}$), 6.58-6.55 (m, 2H, $H_{arom.}$), 3.59-3.55 (m, 1H, OCH_2), 3.05-3.03 (m, 1H, OCH_2) ppm. Monomer (minor component): δ = 7.30 (dd, J = 7.6, 2.6 Hz, 1H, $H_{arom.}$), 6.99 (dd, J = 7.6, 2.6 Hz, 1H, $H_{arom.}$), 6.74 (t, J = 7.6 Hz, 1H, $H_{arom.}$), 4.38 (t, J = 4.6 Hz, 2H, OCH_2), 3.93 (s, 1H, C*H*) ppm. Signals not listed are overlapping and cannot be assigned. **^{1}H NMR** (400 MHz, DMSO-d_6): Dimer (major component): δ = 6.93 (dd, J = 7.9, 1.5 Hz, 1H, $H_{arom.}$), 6.48 (t, J = 7.9 Hz, 1H, $H_{arom.}$), 6.40 (dd, J = 7.9, 1.5 Hz, 1H, $H_{arom.}$), 3.60 (s, 1H, C*H*), 3.45-3.43 (m, 1H, OCH_2), 2.89-2.85 (m, 1H, OCH_2) ppm. Monomer (minor component): δ = 6.73 (dd, J = 7.9, 1.5 Hz, 1H, $H_{arom.}$), 6.23 (t, J = 7.9 Hz, 1H, $H_{arom.}$), 6.09 (dd, J = 7.9, 1.5 Hz, 1H, $H_{arom.}$), 4.03 (t, J = 6.6 Hz, 2H, OCH_2), 3.81 (s, 1H, C*H*) ppm. Signals not listed are overlapping and cannot be assigned. **MS** (negative and positive ESI-MS, MeOH): *m/z* (%) = 1677.5685 (30, $[M_D\text{-}Li^+]$, $C_{90}H_{96}O_{24}Li_3Ti_2^-$, calcd. 1677.5730), 1691.6179 (20, $[M_D\text{+}Li^+]$, $C_{90}H_{96}O_{24}Li_5Ti_2^+$, calcd. 1691.6050). **IR** (KBr): ṽ (cm^{-1}) = 3918, 3294, 3069, 2934, 2858, 2600, 2502, 2298, 2210, 2171, 2115, 2034, 1979, 1900, 1673, 1594, 1556, 1442, 1392, 1346, 1297, 1250, 1207, 1152, 1065, 1000, 913, 851, 804, 740, 680. **Elemental analysis:** $C_{90}H_{96}Li_4O_{24}Ti_2 \cdot 5\ H_2O$: calcd. C = 60.89 %, H = 6.02 %; found C = 59.75 %, H = 5.23 %..

$Li[Li_3(3^{8Non}{}_3Ti)_2]$

Ligand **3^{8Non}**-H_2 (30 mg, 0.11 mmol) is converted into the corresponding complex in methanol (30 mL). The product is obtained after removal of the solvent under reduced pressure as red solid (quantitative). **^{1}H NMR** (600 MHz, MeOH-d_4): Only dimer: δ = 7.10 (dd, J = 6.8, 3.0 Hz, 1H, $H_{arom.}$), 6.59-6.53 (m, 2H, $H_{arom.}$), 3.61-3.58 (m, 1H, OCH_2), 3.08-3.05 (m, 1H, OCH_2), 2.20-2.11 (m, 2H, CH_2), 1.98 (m, 1H, C*H*), 1.48-1.22 (m, 10H, $5\times CH_2$) ppm. **^{1}H NMR** (600 MHz, DMSO-d_6): Dimer (major component): δ = 6.94 (dd, J = 7.9, 1.6 Hz, 1H, $H_{arom.}$), 6.48 (t, J = 7.9 Hz, 1H, $H_{arom.}$), 6.40 (dd, J = 7.9, 1.6 Hz, 1H, $H_{arom.}$), 3.48-3.45 (m, 1H, OCH_2), 2.89-2.86 (m, 1H, OCH_2) ppm. Monomer (minor component): δ = 6.73 (dd, J = 7.9, 1.6 Hz, 1H, $H_{arom.}$), 6.23 (t, J = 7.9 Hz, 1H, $H_{arom.}$), 6.09 (dd, J = 7.9, 1.6 Hz, 1H, $H_{arom.}$), 4.02 (t, J = 6.6 Hz, 2H, OCH_2) ppm. Signals not listed are overlapping and cannot be assigned. **MS** (negative and positive ESI-MS, MeOH): *m/z* (%) = 1761.6688 (100, $[M_D\text{-}Li^+]$, $C_{96}H_{108}O_{24}Li_3Ti_2^-$, calcd. 1761.6669), 1775.7025 (30, $[M_D\text{+}Li^+]$, $C_{96}H_{108}O_{24}Li_5Ti_2^+$, calcd. 1775.6989). **IR** (KBr): ṽ (cm^{-1}) = 3901, 3861, 3747, 3402, 3295, 3085, 2926, 2855, 2652, 2549, 2319, 2271, 2238, 2160, 2112, 2093, 2064, 2029, 1989, 1970, 1944, 1863, 1676, 1595, 1519, 1444, 1295, 1255, 1215, 1153, 1063, 1010, 918, 856, 802, 742, 683. **Elemental analysis:** $C_{96}H_{108}Li_4O_{24}Ti_2 \cdot 15\ H_2O$: calcd. C = 56.53 %, H = 6.82 %; found C = 56.48 %, H = 6.11 %.

Li[Li_3($3^{9Dec}{}_3$Ti)$_2$]

Ligand **3^{9Dec}**-H_2 (50 mg, 0.17 mmol) is converted into the corresponding complex in methanol (50 mL). The product is obtained after removal of the solvent under reduced pressure as red solid (quantitative). **^{1}H NMR** (600 MHz, MeOH-d_4): Only dimer: δ = 7.10 (dd, J = 6.9, 2.8 Hz, 1H, $H_{arom.}$), 6.58-6.55 (m, 2H, $H_{arom.}$), 3.60-3.58 (m, 1H, OCH_2), 3.54 (t, J = 6.7 Hz, 1H, CH), 3.05-3.02 (m, 1H, OCH_2), 2.18-2.11 (m, 2H, CH_2), 1.53-1.47 (m, 4H, 2×CH_2), 1.43-1.40 (m, 2H, CH_2), 1.39-1.31 (m, 4H, 2×CH_2), 1.28-1.24 (m, 2H, CH_2) ppm. **^{1}H NMR** (600 MHz, DMSO-d_6): Dimer (major component): δ = 6.94 (dd, J = 7.9, 1.6 Hz, 1H, $H_{arom.}$), 6.49 (t, J = 7.9 Hz, 1H, $H_{arom.}$), 6.40 (dd, J = 7.9, 1.6 Hz, 1H, $H_{arom.}$), 3.47-3.44 (m, 1H, OCH_2), 2.89-2.85 (m, 1H, OCH_2) ppm. Monomer (minor component): δ = 6.74 (dd, J = 7.9, 1.6 Hz, 1H, $H_{arom.}$), 6.23 (t, J = 7.9 Hz, 1H, $H_{arom.}$), 6.10 (dd, J = 7.9, 1.6 Hz, 1H, $H_{arom.}$), 4.31 (t, J = 5.3 Hz, 2H, OCH_2) ppm. Signals not listed are overlapping and cannot be assigned. **MS** (negative ESI-MS, MeOH): m/z (%) = 1845.7567 (50, [M_D-Li^+], $C_{102}H_{120}O_{24}Li_3Ti_2^-$, calcd. 1845.7608). **IR** (KBr): $\tilde{\nu}$ (cm^{-1}) = 3301, 3068, 2929, 2856, 2686, 2389, 2156, 2117, 2041, 1968, 1895, 1679, 1595, 1558, 1518, 1445, 1346, 1298, 1253, 1216, 1154, 1063, 1012, 919, 858, 806, 744, 685. **Elemental analysis:** $C_{102}H_{120}Li_4O_{24}Ti_2 \cdot 12\ H_2O$: calcd. C = 59.19 %, H = 7.01 %; found C = 59.07 %, H = 7.90 %.

Li[Li_3($3^{10Undec}{}_3$Ti)$_2$]

Ligand **$3^{10Undec}$**-H_2 (50 mg, 0.16 mmol) is converted into the corresponding complex in methanol (50 mL). The product is obtained after removal of the solvent under reduced pressure as red solid (quantitative). **^{1}H NMR** (400 MHz, MeOH-d_4): Only dimer: δ = 7.08 (dd, J = 7.8, 1.6 Hz, 1H, $H_{arom.}$), 6.63-6.53 (m, 2H, $H_{arom.}$), 3.60-3.56 (m, 1H, OCH_2), 3.04-3.01 (m, 1H, OCH_2), 2.15-2.11 (m, 2H, CH_2), 1.45-1.37 (m, 8H, 4×CH_2), 1.25-1.20 (m, 8H, 4×CH_2) ppm. **^{1}H NMR** (400 MHz, DMSO-d_6): Dimer (major component): δ = 6.92 (dd, J = 7.8, 1.5 Hz, 1H, $H_{arom.}$), 6.46 (t, J = 7.8 Hz, 1H, $H_{arom.}$), 6.38 (dd, J = 7.8, 1.5 Hz, 1H, $H_{arom.}$), 3.47-3.45 (m, 1H, OCH_2), 2.86-2.83 (m, 1H, OCH_2) ppm. Monomer (minor component): δ = 6.73 (dd, J = 7.9, 1.5 Hz, 1H, $H_{arom.}$), 6.23 (t, J = 7.9 Hz, 1H, $H_{arom.}$), 6.09 (dd, J = 7.9, 1.5 Hz, 1H, $H_{arom.}$), 4.03 (t, J = 6.6 Hz, 2H, OCH_2) ppm. Signals not listed are overlapping and cannot be assigned. **MS** (negative ESI-MS, MeOH): m/z (%) = 1930.8656 (100, [M_D-Li^+], $C_{108}H_{132}O_{24}Li_3Ti_2^-$, calcd. 1930.8581). **IR** (KBr): $\tilde{\nu}$ (cm^{-1}) = 3295, 3068, 2926, 2855, 2681, 2501, 2295, 2117, 2033, 1974, 1674, 1595, 1556, 1444, 1391, 1295, 1251, 1208, 1064, 1001, 895, 853, 805, 740, 680. **Elemental analysis:** $C_{108}H_{132}O_{24}Li_4Ti_2 \cdot 4\ H_2O$: calcd. C = 64.54 %, H = 7.02 %; found C = 64.02 %, H = 6.83 %.

Ligands 4-H_2

Benzyl-2,3-dihydroxybenzoate (4^{Bn}-H_2):

The ligand is synthesized with benzyl alcohol (1.62 g, 15.0 mmol) according to the general procedure. Column chromatography (pentane/ethyl acetat 6:1, R_f = 0.50) results in the product as a colorless solid (77 %, 562 mg, 2.30 mmol). **M.p.**: 63 °C – 69 °C (last solvent used: DCM). **^{1}H NMR** (400 MHz, $CDCl_3$): δ = 10.87 (s, 1H, OH), 7.46-7.36 (m, 6H, $H_{arom.}$), 7.11 (dd, J = 8.0, 1.4 Hz, 1H, $H_{arom.}$), 6.79 (t, J = 8.0 Hz, 1H, $H_{arom.}$), 5.63 (s, 1H, OH), 5.39 (s, 2H, OCH_2) ppm. **^{13}C NMR** (151 MHz, $CDCl_3$): δ = 170.10 (CO$_2$CH$_2$), 148.91 ($C_{arom.}$), 144.99 ($C_{arom.}$), 135.13 ($C_{arom.}$), 128.70 ($C_{arom.}$), 128.58 ($C_{arom.}$), 128.24 ($C_{arom.}$), 120.66 ($C_{arom.}$), 119.88 ($C_{arom.}$), 119.22 ($C_{arom.}$), 112.38 ($C_{arom.}$), 67.14 (OCH$_2$) ppm. **MS** (positive ESI-MS, MeOH): m/z (%) = 245.0805 (10, [M+H$^+$], $C_{14}H_{13}O_4^+$, calcd. 245.0808). **IR** (KBr): $\tilde{\nu}$ (cm^{-1}) = 3222, 2927, 1690, 1496, 1392, 1309, 1235, 1138, 1072, 855, 736. **Elemental analysis** $C_{14}H_{12}O_4$: calcd. C = 68.85 %, H = 4.95 %; found C = 68.94 %, H = 5.01 %.

2-Methylbenzyl-2,3-dihydroxybenzoate (4^{2Me}-H_2):

The ligand is synthesized with 2-methylbenzyl alcohol (1.84 g, 15.1 mmol) according to the general procedure. Column chromatography (pentane/ethyl acetat 6:1, R_f = 0.53) affords the product as a colorless solid (70 %, 545 mg, 2.10 mmol). **M.p.**: 90 °C – 95 °C (last solvent used: DCM). **^{1}H NMR** (400 MHz, $CDCl_3$): δ = 10.90 (s, 1H, O*H*), 7.40 (td, *J* = 8.0, 1.8 Hz, 2H, $H_{arom.}$), 7.32-7.27 (m, 1H, $H_{arom.}$), 7.25-7.20 (m, 2H, $H_{arom.}$), 7.11 (d, *J* = 8.0 Hz, 1H, $H_{arom.}$), 6.78 (t, *J* = 8.0 Hz, 1H, $H_{arom.}$), 5.63 (s, 1H, O*H*), 5.41 (s, 2H, OCH_2), 2.42 (s, 3H, CH_3) ppm. **^{13}C NMR** (151 MHz, $CDCl_3$): δ = 170.13 (CO_2CH_2), 148.93 ($C_{arom.}$), 145.02 ($C_{arom.}$), 137.18 ($C_{arom.}$), 133.08 ($C_{arom.}$), 130.53 ($C_{arom.}$), 129.44 ($C_{arom.}$), 128.91 ($C_{arom.}$), 126.12 ($C_{arom.}$), 120.60 ($C_{arom.}$), 119.88 ($C_{arom.}$), 119.23 ($C_{arom.}$), 112.40 ($C_{arom.}$), 65.70 (OCH_2), 19.02 (CH_3) ppm. **MS** (positive ESI-MS, MeOH): m/z (%) = 281.0775 (80, [M+Na^+], $C_{15}H_{14}NaO_4^+$, calcd. 281.0784). **IR** (KBr): $\tilde{\nu}$ (cm^{-1}) = 3482, 3143, 1666, 1465, 1379, 1301, 1251, 1144, 1062, 966, 833, 753, 694. **Elemental analysis** $C_{15}H_{14}O_4$: calcd. C = 69.76 %, H = 5.46 %; found C = 69.88 %, H = 5.49 %.

3-Methylbenzyl-2,3-dihydroxybenzoate (4^{3Me}-H_2):

The ligand is synthesized with 3-methylbenzyl alcohol (3.99 g, 32.7 mmol) according to the general procedure. Column chromatography (pentane/ethyl acetat 6:1, R_f = 0.55) yields the product as a colorless solid (57 %, 961 mg, 3.72 mmol). **M.p.**: 85 °C – 90 °C (last solvent used: DCM). **^{1}H NMR** (600 MHz, $CDCl_3$): δ = 10.90 (s, 1H, O*H*), 7.42 (dd, *J* = 8.0, 1.5 Hz, 1H, $H_{arom.}$), 7.32-7.27 (m, 1H, $H_{arom.}$), 7.25 (s, 1H, $H_{arom.}$), 7.24 (d, *J* = 7.2 Hz, 1H, $H_{arom.}$), 7.18 (d, *J* = 7.2 Hz, 1H, $H_{arom.}$), 7.11 (dd, *J* = 8.0, 1.5 Hz, 1H, $H_{arom.}$), 6.79 (t, *J* = 8.0 Hz, 1H, $H_{arom.}$), 5.65 (s, 1H, O*H*), 5.35 (s, 2H, OCH_2), 2.39 (s, 3H, CH_3) ppm. **^{13}C NMR** (151 MHz, $CDCl_3$): δ = 170.28 (CO_2CH_2), 149.06 ($C_{arom.}$), 145.13 ($C_{arom.}$), 138.59 ($C_{arom.}$), 135.19 ($C_{arom.}$), 129.48 ($C_{arom.}$), 129.12 ($C_{arom.}$), 128.75 ($C_{arom.}$), 125.47 ($C_{arom.}$), 120.84 ($C_{arom.}$), 120.01 ($C_{arom.}$), 119.35 ($C_{arom.}$), 112.59 ($C_{arom.}$), 67.36 (OCH_2), 21.54 (CH_3) ppm. **MS** (negative ESI-MS, MeOH): m/z (%) = 257.0847 (100, [M−H^+], $C_{15}H_{13}O_4^-$, calcd. 257.0819). **IR** (KBr): $\tilde{\nu}$ (cm^{-1}) = 3467, 3185, 2958, 2457, 1942, 1860, 1672, 1611, 1468, 1382, 1304, 1149, 1069, 982, 903, 842, 753, 696, 590, 497. **Elemental analysis** $C_{15}H_{14}O_4$: calcd. C = 69.76 %, H = 5.46 %; found C = 69.76 %, H = 5.25 %.

4-Methylbenzyl-2,3-dihydroxybenzoate (4^{4Me}-H_2):

The ligand is synthesized with 4-methylbenzyl alcohol (4.01 g, 32.9 mmol) according to the general procedure. Column chromatography (pentane/ethyl acetat 5:1, R_f = 0.60) affords the product as a colorless solid (66 %, 1.12 g, 4.34 mmol). **M.p.**: 75 °C – 80 °C (last solvent used: DCM). **^{1}H NMR** (600 MHz, $CDCl_3$): δ = 10.90 (s, 1H, O*H*), 7.40 (dd, *J* = 8.0, 1.5 Hz, 1H, $H_{arom.}$), 7.34 (d, *J* = 7.9 Hz, 2H, $H_{arom.}$), 7.21 (d, *J* = 7.9 Hz, 2H, $H_{arom.}$), 7.10 (dd, *J* = 8.0, 1.5 Hz, 1H, $H_{arom.}$), 6.78 (t, *J* = 8.0 Hz, 1H, $H_{arom.}$), 5.65 (s, 1H, O*H*), 5.35 (s, 2H, OCH_2), 2.37 (s, 3H, CH_3) ppm. **^{13}C NMR** (151 MHz, $CDCl_3$): δ = 170.30 (CO_2CH_2), 149.04 ($C_{arom.}$), 145.11 ($C_{arom.}$), 138.66 ($C_{arom.}$), 132.26 ($C_{arom.}$), 129.51 ($C_{arom.}$), 128.58 ($C_{arom.}$), 120.83 ($C_{arom.}$), 119.97 ($C_{arom.}$), 119.32 ($C_{arom.}$), 112.62 ($C_{arom.}$), 67.30 (OCH_2), 21.40 (CH_3) ppm. **MS** (negative ESI-MS, MeOH): m/z (%) = 257.0825 (100, [M−H^+], $C_{15}H_{13}O_4^-$, calcd. 257.0819). **IR** (KBr): $\tilde{\nu}$ (cm^{-1}) = 3479, 2954, 2172, 2118, 1663, 1519, 1461, 1382, 1302, 1268, 1141, 1062, 977, 919, 860, 835, 796, 757, 710. **Elemental analysis** $C_{15}H_{14}O_4$: calcd. C = 69.76 %, H = 5.46 %; found C = 69.81 %, H = 5.77 %.

2,4-Dimethylbenzyl-2,3-dihydroxybenzoate ($4^{2,4Me}$-H_2):

The ligand is synthesized with 2,4-dimethylbenzyl alcohol (1.67 g, 12.2 mmol) according to the general procedure. Column chromatography (pentane/ethyl acetat 6:1, R_f = 0.50) yields the product as a colorless solid (63 %, 420 mg, 1.54 mmol). **M.p.**: 90 °C – 95 °C (last solvent used: DCM). **^{1}H NMR** (600 MHz, $CDCl_3$): δ = 10.93 (s, 1H, O*H*), 7.37 (dd, *J* = 8.0, 1.6 Hz, 1H, $H_{arom.}$), 7.29 (d, *J* = 7.6 Hz, 1H, $H_{arom.}$), 7.10 (dd, *J* = 8.0, 1.6 Hz, 1H, $H_{arom.}$), 7.06 (s, 1H, $H_{arom.}$), 7.04 (d, *J* = 7.6 Hz, 1H, $H_{arom.}$), 6.77 (t, *J* = 8.0 Hz, 1H, $H_{arom.}$), 5.66 (s, 1H, O*H*), 5.37 (s, 2H, OCH_2), 2.39 (s, 3H, CH_3), 2.34 (s, 3H, CH_3). **^{13}C NMR** (151 MHz, $CDCl_3$): δ = 170.34 (CO_2CH_2), 149.05 ($C_{arom.}$), 145.15 ($C_{arom.}$), 139.03 ($C_{arom.}$), 137.57 ($C_{arom.}$), 131.53 ($C_{arom.}$), 130.24 ($C_{arom.}$), 129.94 ($C_{arom.}$), 128.56 ($C_{arom.}$), 126.87 ($C_{arom.}$), 120.78 ($C_{arom.}$), 119.94 ($C_{arom.}$), 119.32 ($C_{arom.}$), 65.85 (OCH_2), 21.29 (CH_3), 19.10 (CH_3) ppm. **MS** (negatives ESI-MS, MeOH): m/z (%) = 271.1001 (44, [M−H$^+$], $C_{16}H_{15}O_4^-$, calcd. 271.0976). **IR** (KBr): $\tilde{\nu}$ (cm^{-1}) = 3417, 3208, 2920, 1904, 1663, 1605, 1467, 1393, 1302, 1232, 1147, 1068, 977, 853, 815, 745, 676. **Elemental analysis** $C_{16}H_{16}O_4$: calcd. C = 70.58 %, H = 5.92 %; found C = 70.43 %, H = 5.93 %.

2,5-Dimethylbenzyl-2,3-dihydroxybenzoate ($4^{2,5Me}$-H_2):

The ligand is synthesized with 2,5-dimethylbenzyl alcohol (2.50 g, 18.3 mmol) according to the general procedure. Column chromatography (pentane/ethyl acetat 5:1, R_f = 0.55) affords the product as a colorless solid (44 %, 439 mg, 1.61 mmol). **M.p.**: 85 °C – 90 °C (last solvent used: DCM). **^{1}H NMR** (600 MHz, $CDCl_3$): δ = 10.90 (s, 1H, O*H*), 7.38 (dd, *J* = 8.1, 1.4 Hz, 1H, $H_{arom.}$), 7.21 (s, 1H, $H_{arom.}$), 7.15-7.08 (m, 3H, $H_{arom.}$), 6.78 (t, *J* = 8.1 Hz, 1H, $H_{arom.}$), 5.65 (s, 1H, O*H*), 5.37 (s, 2H, OCH_2), 2.38 (s, 3H, CH_3), 2.35 (s, 3H, CH_3) ppm. **^{13}C NMR** (151 MHz, $CDCl_3$): δ = 170.29 (CO_2CH_2), 149.06 ($C_{arom.}$), 145.15 ($C_{arom.}$), 135.78 ($C_{arom.}$), 134.23 ($C_{arom.}$), 132.96 ($C_{arom.}$), 130.60 ($C_{arom.}$), 130.46 ($C_{arom.}$), 129.73 ($C_{arom.}$), 120.77 ($C_{arom.}$), 119.98 ($C_{arom.}$), 119.34 ($C_{arom.}$), 112.59 ($C_{arom.}$), 65.98 (OCH_2), 21.06 (CH_3), 18.69 (CH_3) ppm. **MS** (negative ESI-MS, MeOH): m/z (%) = 271.0978 (100, [M−H$^+$], $C_{16}H_{15}O_4^-$, calcd. 271.0976). **IR** (KBr): $\tilde{\nu}$ (cm^{-1}) = 3510, 3408, 3143, 2924, 2020, 1746, 1667, 1602, 1390, 1307, 1270, 1234, 1145, 1070, 1015, 976, 884, 849, 806, 744, 659. **Elemental analysis** $C_{16}H_{16}O_4$: calcd. C = 70.58 %, H = 5.92 %; found C = 69.97 %, H = 6.05 %.

2,6-Dimethylbenzyl-2,3-dihydroxybenzoate ($4^{2,6Me}$-H_2):

The ligand is synthesized with 2,6-dimethylbenzyl alcohol (1.03 g, 7.60 mmol) according to the general procedure. Column chromatography (pentane/ethyl acetat 7:1, R_f = 0.45) affords the product as a colorless solid (22 %, 91 mg, 0.33 mmol). **M.p.**: 110 °C – 116 °C (last used solvent: DCM). **^{1}H NMR** (400 MHz, $CDCl_3$): δ = 10.95 (s, 1H, O*H*), 7.31 (dd, *J* = 8.0, 1.5 Hz, 1H, $H_{arom.}$), 7.19 (t, *J* = 7.4 Hz, 1H, $H_{arom.}$), 7.09 (m, 3H, H_{arom}), 6.75 (t, *J* = 8.0 Hz, 1H, $H_{arom.}$), 5.63 (s, 1H, O*H*), 5.48 (s, 2H, OCH_2), 2.45 (s, 6H, 2xCH_3) ppm. **^{13}C NMR** (151 MHz, $CDCl_3$): δ = 170.33 (CO_2CH_2), 148.86 ($C_{arom.}$), 144.98 ($C_{arom.}$), 138.46 ($C_{arom.}$), 131.22 ($C_{arom.}$), 129.02 ($C_{arom.}$), 128.40 ($C_{arom.}$), 120.67 ($C_{arom.}$), 119.77 ($C_{arom.}$), 119.15 ($C_{arom.}$), 112.39 ($C_{arom.}$), 62.18 (OCH_2), 19.73 (2xCH_3) ppm. **MS** (positive ESI-MS, MeOH): m/z (%) = 279.1222 (10, [M+Li$^+$], $C_{16}H_{16}LiO_4^+$, calcd. 279.1203). **IR** (KBr): $\tilde{\nu}$ (cm^{-1}) = 3468, 2960, 1665, 1464, 1378, 1265, 1144, 1066, 969, 923, 833, 747. **Elemental analysis** $C_{16}H_{16}O_4$: calcd. C = 70.58 %, H = 5.92 %; found C = 69.95 %, H = 5.92 %.

3,5-Dimethylbenzyl-2,3-dihydroxybenzoate ($4^{3,5Me}$-H_2):

The ligand is synthesized with 3,5-dimethylbenzyl alcohol (2.03 g, 14.89 mmol) according to the general procedure. Column chromatography (pentane/ethyl acetat 7:1, R_f = 0.45) affords the product as a colorless solid (36 %, 292 mg, 1.07 mmol). **M.p.**: 69 °C – 75 °C (last solvent used: DCM). **^{1}H NMR**

(600 MHz, $CDCl_3$): δ = 10.91 (s, 1H, O*H*), 7.42 (dd, *J* = 8.0, 1.6 Hz, 1H, $H_{arom.}$), 7.11 (m, 1H, $H_{arom.}$), 7.04 (s, 2H, $H_{arom.}$), 7.00 (s, 1H, $H_{arom.}$), 6.79 (t, *J* = 8.0 Hz, 1H, $H_{arom.}$), 5.63 (s, 1H, O*H*), 5.32 (s, 2H, OCH_2), 2.34 (s, 6H, 2xCH_3) ppm. **^{13}C NMR** (151 MHz, $CDCl_3$): δ = 170.14 (CO_2CH_2), 148.89 ($C_{arom.}$), 144.97 ($C_{arom.}$), 138.34 ($C_{arom.}$), 134.97 ($C_{arom.}$), 130.21 ($C_{arom.}$), 126.06 ($C_{arom.}$), 120.72 ($C_{arom.}$), 119.80 ($C_{arom.}$), 119.17 ($C_{arom.}$), 112.47 ($C_{arom.}$), 67.25 (OCH_2), 21.26 (2xCH_3) ppm. **MS** (positive ESI-MS, MeOH): m/z (%) = 295.0955 (55, [$M+Na^+$], $C_{16}H_{16}NaO_4^+$, calcd. 295.0941). **IR** (KBr): $\tilde{\nu}$ (cm^{-1}) = 3522, 3119, 2922, 1663, 1605, 1463, 1382, 1306, 1261, 1151, 1067, 1005, 841, 748, 694. **Elemental analysis** $C_{16}H_{16}O_4$: calcd. C = 70.58 %, H = 5.92 %; found C = 70.50 %, H = 6.17 %.

2,4,6-Trimethylbenzyl-2,3-dihydroxybenzoate ($4^{2,4,6Me}$-H_2):

The ligand is synthesized with 2,4,6-trimethylbenzyl alcohol (2.31 g, 15.4 mmol) according to the general procedure. Column chromatography (pentane/ethyl acetat 5:1, R_f = 0.55) yields the product as a colorless solid (17 %, 150 mg, 0.52 mmol). **M.p.**: 173 °C – 180 °C (last solvent used: DCM). **^{1}H NMR** (300 MHz, $CDCl_3$): δ = 10.96 (s, 1H, O*H*), 7.31 (dd, *J* = 8.0, 1.6 Hz, 1H, $H_{arom.}$), 7.08 (m, 1H, $H_{arom.}$), 6.91 (s, 2H, $H_{arom.}$), 6.74 (t, *J* = 8.0 Hz, 1H, $H_{arom.}$), 5.61 (s, 1H, O*H*), 5.44 (s, 2H, OCH_2), 2.41 (s, 6H, 2xCH_3), 2.30 (s, 3H, CH_3) ppm. **^{13}C NMR** (151 MHz, $CDCl_3$): δ = 170.75 (CO_2CH_2), 149.21 ($C_{arom.}$), 145.33 ($C_{arom.}$), 139.22 ($C_{arom.}$), 138.74 ($C_{arom.}$), 129.52 ($C_{arom.}$), 128.64 ($C_{arom.}$), 121.06 ($C_{arom.}$), 120.09 ($C_{arom.}$), 119.47 ($C_{arom.}$), 112.83 ($C_{arom.}$), 62.49 (OCH_2), 21.42 (CH_3), 19.99 (2xCH_3) ppm. **MS** (positive ESI-MS, MeOH): m/z (%) = 309.1096 (100, [$M+Na^+$], $C_{17}H_{18}NaO_4^+$, calcd. 309.1097). **IR** (KBr): $\tilde{\nu}$ (cm^{-1}) = 3501, 2918, 1664, 1465, 1373, 1271, 1141, 1067, 970, 915, 846, 753. **Elemental analysis** $C_{17}H_{18}O_4$: calcd. C = 71.31 %, H = 6.34 %; found C = 71.20 %, H = 6.35 %.

2,3,5,6-Tetramethylbenzyl-2,3-dihydroxybenzoate ($4^{2,3,5,6Me}$-H_2):

The ligand is synthesized with 2,3,5,6-tetramethylbenzyl alcohol (1.64 g, 10.0 mmol) according to the general procedure. Column chromatography (pentane/ethyl acetat 6:1, R_f = 0.50) affords the product as a colorless solid (36 %, 216 mg, 0.72 mmol). **M.p.**: 130 °C – 135 °C (last solvent used: DCM). **^{1}H NMR** (600 MHz, $CDCl_3$): δ = 10.90 (s, 1H, O*H*), 7.31 (dd, *J* = 8.0, 1.5 Hz, 1H, $H_{arom.}$), 7.09 (dd, *J* = 8.0, 1.5 Hz, 1H, $H_{arom.}$), 7.03 (s, 1H, $H_{arom.}$) 6.74 (t, *J* = 8.0 Hz, 1H, $H_{arom.}$), 5.65 (s, 1H, O*H*), 5.52 (s, 2H, OCH_2), 2.31 (s, 6H, 2xCH_3), 2.27 (s, 6H, 2xCH_3) ppm. **^{13}C NMR** (151 MHz, $CDCl_3$): δ = 170.54 (CO_2CH_2), 148.99 ($C_{arom.}$), 145.11 ($C_{arom.}$), 134.58 ($C_{arom.}$), 134.26 ($C_{arom.}$), 132.75 ($C_{arom.}$), 131.17 ($C_{arom.}$), 120.92 ($C_{arom.}$), 119.88 ($C_{arom.}$), 119.25 ($C_{arom.}$), 112.64 ($C_{arom.}$), 62.90 (OCH_2), 20.55 (2xCH_3), 15.63 (2xCH_3) ppm. **MS** (negative ESI-MS, MeOH): m/z (%) = 299.1298 (100, [$M-H^+$], $C_{18}H_{19}O_4^-$, calcd. 299.1289). **IR** (KBr): $\tilde{\nu}$ (cm^{-1}) = 3425, 3125, 2966, 2919, 2102, 1664, 1604, 1467, 1399, 1303, 1268, 1150, 1064, 1016, 971, 932, 870, 848, 797, 744, 683. **Elemental analysis** $C_{18}H_{20}O_4$: calcd. C = 71.98 %, H = 6.71 %; found C = 71.81 %, H = 6.78 %.

2,3,4,5,6-Pentamethylbenzyl-2,3-dihydroxy-benzoate (4^{Me5}-H_2):

The ligand is synthesized with 2,3,4,5,6-pentamethylbenzyl alcohol (1.63 g, 9.16 mmol) according to the general procedure. Column chromatography (pentane/ethyl acetat 7:1, R_f = 0.45) yields the product as a colorless solid (37 %, 213 mg, 0.68 mmol). **M.p.**: 133 °C – 138 °C (last solvent used: DCM). **^{1}H NMR** (600 MHz, $CDCl_3$): δ = 10.90 (s, 1H, O*H*), 7.32 (dd, *J* = 8.0, 1.5 Hz, 1H, $H_{arom.}$), 7.08 (dd, *J* = 8.0, 1.5 Hz, 1H, $H_{arom.}$), 6.73 (t, *J* = 8.0 Hz, 1H, $H_{arom.}$), 5.65 (s, 1H, O*H*), 5.53 (s, 2H, OCH_2), 2.36 (s, 6H, 2xCH_3), 2.28 (s, 3H, CH_3), 2.26 (s, 6H, 2xCH_3) ppm. **^{13}C NMR** (151 MHz, $CDCl_3$): δ = 170.59 (CO_2CH_2), 148.98 ($C_{arom.}$), 145.09 ($C_{arom.}$), 136.50 ($C_{arom.}$), 134.23 ($C_{arom.}$), 133.13 ($C_{arom.}$), 128.60 ($C_{arom.}$),

120.97 ($C_{arom.}$), 119.85 ($C_{arom.}$), 119.20 ($C_{arom.}$), 112.69 ($C_{arom.}$), 63.44 (O*C*H_2), 17.35 (1x*C*H_3), 16.92 (2x*C*H_3), 16.63 (2x*C*H_3) ppm. **MS** (negative ESI-MS, MeOH): m/z (%) = 313.1451 (30, $[M-H^+]$, $C_{19}H_{21}O_4^-$, calcd. 313.1445). **IR** (KBr): ṽ (cm^{-1}) = 3485, 2922, 1662, 1606, 1464, 1381, 1302, 1269, 1147, 1065, 973, 924, 852, 819, 756, 709. **Elemental analysis** $C_{19}H_{22}O_4$: calcd. C = 72.59 %, H = 7.05 %; found C = 72.94 %, H = 7.30 %.

4-Isopropylbenzyl-2,3-dihydroxybenzoate (4^{4iPr}-H_2):

The ligand is synthesized with 4-isopropylbenzyl alcohol (2.41 g, 16.07 mmol) according to the general procedure. Column chromatography (pentane/ethyl acetat 10:1, R_f = 0.40) results in the product as a colorless solid (59 %, 543 mg, 1.90 mmol). **M.p.**: 115 °C – 120 °C (last solvent used: DCM). **^{1}H NMR** (600 MHz, $CDCl_3$): δ = 10.90 (s, 1H, O*H*), 7.41 (dd, *J* = 8.0, 1.5 Hz, 1H, $H_{arom.}$), 7.37 (d, *J* = 8.1 Hz, 2H, $H_{arom.}$), 7.26 (d, *J* = 8.1 Hz, 2H, $H_{arom.}$), 7.10 (dd, *J* = 8.0, 1.5 Hz, 1H, $H_{arom.}$), 6.78 (t, *J* = 8.0 Hz, 1H, $H_{arom.}$), 5.65 (s, 1H, O*H*), 5.36 (s, 2H, OCH_2), 2.93 (hept, *J* = 6.9 Hz, 1H, C*H*), 1.26 (d, *J* = 6.9 Hz, 6H, 2xCH_3) ppm. **^{13}C NMR** (151 MHz, $CDCl_3$): δ = 170.32 (*C*O_2CH_2), 149.60 ($C_{arom.}$), 149.05 ($C_{arom.}$), 145.12 ($C_{arom.}$), 132.63 ($C_{arom.}$), 128.65 ($C_{arom.}$), 126.92 ($C_{arom.}$), 120.85 ($C_{arom.}$), 119.97 ($C_{arom.}$), 119.33 ($C_{arom.}$), 112.63 ($C_{arom.}$), 67.28 (O*C*H_2), 34.07 (*C*H), 24.09 (2x*C*H_3) ppm. **MS** (negative ESI-MS, MeOH): m/z (%) = 285.1125 (100, $[M-H^+]$, $C_{17}H_{17}O_4^-$, calcd. 285.1132). **IR** (KBr): ṽ (cm^{-1}) = 3765, 3482, 3054, 2961, 2457, 1910, 1672, 1615, 1468, 1385, 1304, 1267, 1148, 1066, 978, 826, 753, 701, 600. **Elemental analysis** $C_{17}H_{18}O_4$: calcd. C = 71.31 %, H = 6.34 %; found C = 71.28 %, H = 6.25 %.

Complexes $Li[Li_3(4_3Ti)_2]$

$Li[Li_3(4^{Bn}{}_3Ti)_2]$

The complex is obtained from the corresponding ligand **4^{Bn}**-H_2 (100.0 mg, 0.41 mmol) in methanol (25 mL) following the general procedure as a red solid (quantitative). **^{1}H NMR** (300 MHz, DMSO-*d_6*): Dimer (minor component): δ = 7.05 (dd, *J* = 8.0, 1.8 Hz, 1H, $H_{arom.}$), 6.53 (dd, *J* = 8.0, 1.8 Hz, 1H, $H_{arom.}$), 6.47 (t, *J* = 8.0 Hz, 1H, $H_{arom.}$), 4.59 (d, *J* = 12.7 Hz, 1H, OCH_2), 3.97 (d, *J* = 12.7 Hz, 1H, OCH_2) ppm. Monomer (major component): δ = 6.83 (dd, *J* = 8.0, 1.6 Hz, 1H, $H_{arom.}$), 6.30 (t, *J* = 8.0 Hz, 1H, $H_{arom.}$), 6.18 (dd, *J* = 8.0, 1.6 Hz, 1H, $H_{arom.}$), 5.19 (s, 2H, OCH_2) ppm. Signals not listed are overlapping and cannot be assigned. **MS** (positive ESI-MS, MeOH): m/z (%) = 1583.3228 (70, $[M+Li^+]$, $C_{84}H_{60}O_{24}Li_5Ti_2^+$, calcd. 1583.3228). **IR** (KBr): ṽ (cm^{-1}) = 3875, 3360, 2306, 2104, 1675, 1592, 1383, 1293, 1212, 1067, 1000, 841, 787, 742, 682. **Elemental analysis** $C_{84}H_{60}O_{24}Li_4Ti_2 \cdot 4\ H_2O$: calcd. C = 61.19 %, H = 4.16 %; found C = 60.90 %, H = 4.30 %.

$Li[Li_3(4^{2Me}{}_3Ti)_2]$

The complex is obtained from the corresponding ligand **4^{2Me}**-H_2 (100.0 mg, 0.39 mmol) in methanol (25 mL) using the general procedure as a red solid (quantitative). **^{1}H NMR** (400 MHz, DMSO-*d_6*): Dimer (minor component): δ = 7.00 (d, *J* = 7.3 Hz, 1H, $H_{arom.}$), 4.58 (d, *J* = 13.0 Hz, 1H, OCH_2), 3.95 (d, *J* = 13.0 Hz, 1H, OCH_2), 2.11 (s, 3H, CH_3) ppm. Monomer (major component): δ = 6.80 (dd, *J* = 8.0, 1.6 Hz, 1H, $H_{arom.}$), 6.28 (t, *J* = 8.0 Hz, 1H, $H_{arom.}$), 6.16 (m, 1H, $H_{arom.}$), 5.15 (s, 2H, OCH_2), 2.29 (s, 3H, CH_3) ppm. Signals not listed are overlapping and cannot be assigned. **MS** (positive ESI-MS, MeOH): m/z (%) = 1667.4221 (100, $[M+Li^+]$, $C_{90}H_{72}O_{24}Li_5Ti_2^+$, calcd. 1667.4167). **IR** (KBr): ṽ (cm^{-1}) = 3786, 3372, 2288, 2119, 2039, 1744, 1675, 1594, 1443, 1381, 1293, 1213, 1151, 1066, 999, 804, 742. **Elemental analysis** $C_{90}H_{72}O_{24}Li_4Ti_2 \cdot 4\ H_2O$: calcd. C = 62.37 %; H = 4.65 %; found C = 62.19 %; H = 4.76 %.

Li[Li$_3$(4^{3Me}$_3$Ti)$_2$]

The complex is obtained from the corresponding ligand **4^{3Me}**-H$_2$ (100.0 mg, 0.39 mmol) in methanol (25 mL) utilizing the general procedure as a red solid (quantitative). **^{1}H NMR** (300 MHz, DMSO-d_6): Dimer (minor component): δ = 4.54 (d, J = 12.7 Hz, 1H, OCH_2), 3.92 (d, J = 12.7 Hz, 1H, OCH_2), 2.23 (s, 3H, CH_3) ppm. Monomer (major component): δ = 6.81 (dd, J = 7.8, 1.4 Hz, 1H, H$_{arom.}$), 6.28 (t, J = 7.8 Hz, 1H, H$_{arom.}$), 6.16 (dd, J = 7.8, 1.4 Hz, 1H, H$_{arom.}$), 5.14 (s, 2H, OCH_2), 2.27 (s, 3H, CH_3) ppm. Signals not listed are overlapping and cannot be assigned. **MS** (negative ESI-MS, MeOH): m/z (%) = 1653.4002 (100, [M−Li$^+$], $C_{90}H_{72}O_{24}Li_3Ti_2^-$, calcd. 1653.3853). **IR** (KBr): ṽ (cm^{-1}) = 3357, 2951, 2327, 2110, 1846, 1675, 1592, 1559, 1491, 1442, 1377, 1342, 1292, 1251, 1213, 1152, 1064, 997, 922, 886, 853, 813, 738, 680. **Elemental analysis** $C_{90}H_{72}O_{24}Li_4Ti_2 \cdot 4\ H_2O$: calcd. C = 62.37 %, H = 4.65 %; found C = 62.59 %, H = 4.68 %.

Li[Li$_3$(4^{4Me}$_3$Ti)$_2$]

The complex is obtained from ligand **4^{4Me}**-H$_2$ (100.0 mg, 0.39 mmol) in methanol (25 mL) using the general procedure as a red solid (quantitative). **^{1}H NMR** (300 MHz, DMSO-d_6): Dimer (minor component): δ = 4.54 (d, J = 12.6 Hz, 1H, OCH_2), 3.92 (d, J = 12.6 Hz, 1H, OCH_2), 2.24 (s, 3H, CH_3) ppm. Monomer (major component): δ = 6.80 (dd, J = 7.8, 1.6 Hz, 1H, H$_{arom.}$), 6.29 (t, J = 7.8 Hz, 1H, H$_{arom.}$), 6.17 (dd, J = 7.8, 1.6 Hz, 1H, H$_{arom.}$), 5.13 (s, 2H, OCH_2), 2.23 (s, 3H, CH_3) ppm. Signals not listed are overlapping and cannot be assigned. **MS** (negative ESI-MS, MeOH): m/z (%) = 1653.3943 (100, [M−Li$^+$], $C_{90}H_{72}O_{24}Li_3Ti_2^-$, calcd. 1653.3853). **IR** (KBr): ṽ (cm^{-1}) = 3358, 2922, 2323, 2091, 1898, 1675, 1592, 1519, 1442, 1378, 1292, 1251, 1213, 1152, 1063, 997, 846, 800, 741, 678. **Elemental analysis** $C_{90}H_{72}O_{24}Li_4Ti_2 \cdot 5\ H_2O$: calcd. C = 61.73 %, H = 4.72 %; found C = 61.97 %, H = 4.63 %.

Li[Li$_3$(4^{2,4Me}$_3$Ti)$_2$]

The complex is obtained from the corresponding ligand **4^{2,4Me}**-H$_2$ (100.0 mg, 0.37 mmol) in methanol (25 mL) with the general procedure as a red solid (quantitative). **^{1}H NMR** (300 MHz, DMSO-d_6): Dimer (minor component): δ = 6.71 (d, J = 7.9 Hz, 1H, H$_{arom.}$), 4.56 (d, J = 12.8 Hz, 1H, OCH_2), 3.90 (d, J = 12.8 Hz, 1H, OCH_2) ppm. Monomer (major component): δ = 7.39 (d, J = 7.9 Hz, 1H, H$_{arom.}$), 6.78 (dd, J = 7.9, 1.7 Hz, 1H, H$_{arom.}$), 6.28 (t, J = 7.9 Hz, 1H, H$_{arom.}$), 6.15 (d, J = 7.3 Hz, 1H, H$_{arom.}$), 5.10 (s, 2H, OCH_2), 2.25 (s, 3H, CH_3), 2.21 (s, 3H, CH_3) ppm. Signals not listed are overlapping and cannot be assigned. **MS** (negative ESI-MS, MeOH): m/z (%) = 1737.4872 (100, [M−Li$^+$], $C_{96}H_{84}O_{24}Li_3Ti_2^-$, calcd. 1737.4792). **IR** (KBr): ṽ (cm^{-1}) = 3362, 2920, 2311, 2078, 1895, 1677, 1593, 1562, 1506, 1443, 1378, 1292, 1252, 1214, 1186, 1151, 1063, 999, 806, 744, 680. **Elemental analysis** $C_{96}H_{84}O_{24}Li_4Ti_2 \cdot 3\ H_2O$: calcd. C = 64.09 %, H = 5.04 %; found C = 63.74 %, H = 4.93 %.

Li[Li$_3$(4^{2,5Me}$_3$Ti)$_2$]

The complex is obtained from the corresponding ligand **4^{2,5Me}**-H$_2$ (100.0 mg, 0.37 mmol) in methanol (25 mL) applying the general procedure as a red solid (quantitative). **^{1}H NMR** (300 MHz, DMSO-d_6): Dimer (minor component): δ = 4.54 (d, J = 12.6 Hz, 1H, OCH_2), 3.89 (d, J = 12.6 Hz, 1H, OCH_2), 2.14 (s, 3H, CH_3), 2.05 (s, 3H, CH_3) ppm. Monomer (major component): δ = 7.21 (s, 1H, H$_{arom.}$), 6.77 (dd, J = 7.7, 1.5 Hz, 1H, H$_{arom.}$), 6.26 (t, J = 7.7 Hz, 1H, H$_{arom.}$), 6.12 (dd, J = 7.7, 1.5 Hz, 1H, H$_{arom.}$), 5.11 (s, 2H,

OCH_2), 2.25 (s, 3H, CH_3), 2.23 (s, 3H, CH_3) ppm. Signals not listed are overlapping and cannot be assigned. **MS** (negative ESI-MS, MeOH): m/z (%) = 1737.4932 (100, [M−Li$^+$], $C_{96}H_{84}O_{24}Li_3Ti_2^-$, calcd. 1737.4792). **IR** (KBr): ṽ (cm^{-1}) = 3364, 2920, 2323, 2113, 1889, 1677, 1593, 1505, 1443, 1376, 1293, 1252, 1214, 1153, 1064, 998, 851, 810, 742, 681. **Elemental analysis** $C_{96}H_{84}O_{24}Li_4Ti_2 \cdot 5\ H_2O$: calcd. C = 62.83 %, H = 5.16 %; found C = 62.74 %, H = 5.10 %.

Li[Li$_3$(4^{2,6Me}$_3$Ti)$_2$]

The complex is obtained from the corresponding ligand **4^{2,6Me}**-H_2 (60.0 mg, 0.22 mmol) in methanol (25 mL) as a red solid (quantitative). **^{1}H NMR** (400 MHz, DMSO-d_6): Dimer (minor component): δ = 6.58 (t, J = 7.8 Hz, 1H, $H_{arom.}$), 6.52 (dd, J = 7.8, 1.7 Hz, 1H, $H_{arom.}$), 4.86 (d, J = 12.2 Hz, 1H, OCH_2), 3.98 (d, J = 12.2 Hz, 1H, OCH_2), 2.11 (s, 6H, 2xCH_3) ppm. Monomer (major component): δ = 6.71 (dd, J = 8.0, 1.6 Hz, 1H, $H_{arom.}$), 6.24 (t, J = 8.0 Hz, 1H, $H_{arom.}$), 6.12 (dd, J = 8.0 Hz, 1.6 Hz, 1H, $H_{arom.}$), 5.19 (s, 2H, OCH_2), 2.37 (s, 6H, 2xCH_3) ppm. Signals not listed are overlapping and cannot be assigned. **MS** (negative ESI-MS, MeOH): m/z (%) = 1737.5082 (100, [M-Li$^+$], $C_{96}H_{84}O_{24}Li_3Ti_2^-$, calcd. 1737.4797). **IR** (KBr): ṽ (cm^{-1}) = 3380, 1679, 1443, 1380, 1294, 1250, 1213, 1151, 1062, 987, 792, 744, 682. **Elemental analysis** $C_{96}H_{84}O_{24}Li_4Ti_2 \cdot 6\ H_2O$: calcd. C = 62.22 %, H = 5.22 %; found C = 62.16 %, H = 5.31 %.

Li[Li$_3$(4^{3,5Me}$_3$Ti)$_2$]

The complex is obtained from the corresponding ligand **4^{3,5Me}**-H_2 (100.0 mg, 0.37 mmol) in methanol (25 mL) as a red solid (quantitative). **^{1}H NMR** (400 MHz, DMSO-d_6): Dimer (minor component): δ = 4.49 (d, J = 12.5 Hz, 1H, OCH_2), 3.86 (d, J = 12.5 Hz, 1H, OCH_2), 2.18 (s, 6H, 2xCH_3) ppm. Monomer (major component): δ = 6.27 (t, J = 7.4 Hz, 1H, $H_{arom.}$), 6.14 (dd, J = 7.4, 1.6 Hz, 1H, $H_{arom.}$), 5.09 (s, 2H, OCH_2), 2.23 (s, 6H, 2xCH_3) ppm. Signals not listed are overlapping and cannot be assigned. **MS** (negative ESI-MS, MeOH): m/z (%) = 1737.5149 (100, [M-Li$^+$], $C_{96}H_{84}O_{24}Li_3Ti_2^-$, calcd. 1737.4797). **IR** (KBr): ṽ (cm^{-1}) = 3369, 2919, 2049, 1676, 1598, 1443, 1379, 1443, 1379, 1293, 1212, 1066, 990, 842, 810, 685. **Elemental analysis** $C_{96}H_{84}O_{24}Li_4Ti_2 \cdot 4\ H_2O$: calcd. C = 63.45 %, H = 5.10 %; found C = 63.47 %, H = 5.21 %.

Li[Li$_3$(4^{2,4,6Me}$_3$Ti)$_2$]

The complex is obtained from **4^{2,4,6Me}**-H_2 (100.0 mg, 0.35 mmol) in methanol (25 mL) as a red solid (quantitative). **^{1}H NMR** (400 MHz, DMSO-d_6): Dimer (minor component): δ = 6.89 (s, 2H, $H_{arom.}$), 6.56 (t, J = 8.0 Hz, 1H, $H_{arom.}$), 6.50 (d, J = 8.0 Hz, 1H, $H_{arom.}$), 4.83 (d, J = 13.4 Hz, 1H, OCH_2), 3.91 (d, J = 13.4 Hz, 1H, OCH_2), 2.05 (s, 6H, 2xCH_3) ppm. Monomer (major component): δ = 6.79 (s, 2H, $H_{arom.}$), 6.69 (dd, J = 8.0, 1.6 Hz, 1H, $H_{arom.}$), 6.23 (t, J = 8.0 Hz, 1H, $H_{arom.}$), 6.11 (d, J = 8.0 Hz, 1H, $H_{arom.}$), 5.15 (s, 2H, OCH_2), 2.33 (s, 6H, 2xCH_3), 2.19 (s, 3H, CH_3) ppm. Signals not listed are overlapping and cannot be assigned. **MS** (negative ESI-MS, MeOH): m/z (%) = 1821.5764 (100, [M-Li$^+$], $C_{102}H_{96}O_{24}Li_3Ti_2^-$, calcd. 1821.5736). **IR** (KBr): ṽ (cm^{-1}) = 3359, 2920, 1675, 1595, 1560, 1443, 1377, 1291, 1214, 1152, 1066, 986, 848, 746, 679. **Elemental analysis** $C_{102}H_{96}O_{24}Li_4Ti_2 \cdot 4\ H_2O$: calcd. C = 64.43 %, H = 5.51 %; found C = 64.62 %, H = 5.63 %.

Li[Li$_3$(4^{2,3,5,6Me}$_3$Ti)$_2$]

The complex is obtained from $\mathbf{4}^{2,3,5,6Me}$-H_2 (100.0 mg, 0.33 mmol) in methanol (25 mL) as a red solid (quantitative). **^{1}H NMR** (400 MHz, DMSO-d_6): Dimer (minor component): δ = 6.95 (dd, J = 7.8, 1.5 Hz, 1H, $H_{arom.}$), 6.87 (s, 1H, $H_{arom.}$), 6.57 (t, J = 7.8 Hz, 1H, $H_{arom.}$), 6.51 (dd, J = 7.8, 1.5 Hz, 1H, $H_{arom.}$), 4.92 (d, J = 12.3 Hz, 1H, OCH_2), 3.98 (d, J = 12.3 Hz, 1H, OCH_2), 2.08 (s, 6H, 2xCH_3), 1.94 (s, 6H, 2xCH_3) ppm. Monomer (major component): δ = 6.90 (s, 1H, $H_{arom.}$), 6.69 (dd, J = 7.8, 1.6 Hz, 1H, $H_{arom.}$), 6.23 (t, J = 7.8 Hz, 1H, $H_{arom.}$), 6.11 (dd, J = 7.8, 1.6 Hz, 1H, $H_{arom.}$), 5.22 (s, 2H, OCH_2), 2.22 (s, 6H, 2xCH_3), 2.13 (s, 6H, 2xCH_3) ppm. MS (negative ESI-MS, MeOH): m/z (%) = 1905.6804 (100, [M−Li^+], $C_{108}H_{108}O_{24}Li_3Ti_2^-$, calcd. 1905.6675). **IR** (KBr): $\tilde{\nu}$ (cm^{-1}) = 3362, 2922, 1679, 1594, 1444, 1375, 1216, 1151, 1061, 993, 789, 742, 684. **Elemental analysis** $C_{108}H_{108}O_{24}Li_4Ti_2 \cdot 4\ H_2O$: calcd. C = 65.33 %, H = 5.89 %; found C = 65.03 %, H = 5.80 %.

Li[Li$_3$(4^{Me5}$_3$Ti)$_2$]

The complex is obtained from $\mathbf{4}^{Me5}$-H_2 (100.0 mg, 0.32 mmol) in methanol (25 mL) as a red solid (quantitative). **^{1}H NMR** (300 MHz, DMSO-d_6): Dimer (minor component): δ = 6.93 (dd, J = 7.8, 1.8 Hz, 1H, $H_{arom.}$), 6.57 (t, J = 7.8 Hz, 1H, $H_{arom.}$), 6.50 (dd, J = 7.8, 1.8 Hz, 1H, $H_{arom.}$), 4.99 (d, J = 12.5 Hz, 1H, OCH_2), 3.96 (d, J = 12.5 Hz, 1H, OCH_2) ppm. Monomer (major component): δ = 6.68 (dd, J = 7.8, 1.7 Hz, 1H, $H_{arom.}$), 6.22 (t, J = 7.8 Hz, 1H, $H_{arom.}$), 6.11 (dd, J = 7.8, 1.7 Hz, 1H, $H_{arom.}$), 5.21 (s, 2H, OCH_2) ppm. Signals not listed are overlapping and cannot be assigned. **MS** (negative ESI-MS, MeOH): m/z (%) = 1989.7716 (92, [M−Li^+], $C_{114}H_{120}O_{24}Li_3Ti_2^-$, calcd. 1989.7614). **IR** (KBr): $\tilde{\nu}$ (cm^{-1}) = 3359, 2920, 2319, 2118, 1896, 1677, 1593, 1527, 1444, 1376, 1216, 1151, 1063, 993, 789, 743, 685. **Elemental analysis** $C_{114}H_{120}O_{24}Li_4Ti_2 \cdot 5\ H_2O$: calcd. C = 65.58 %, H = 6.28 %; found C = 65.58 %, H = 6.43 %.

Li[Li$_3$(4^{4iPr}$_3$Ti)$_2$]

The complex is obtained from $\mathbf{4}^{4iPr}$-H_2 (100.0 mg, 0.32 mmol) in methanol (25 mL) as a red solid (quantitative). **^{1}H NMR** (300 MHz, DMSO-d_6): Dimer (minor component): δ = 7.04 (dd, J = 7.9, 1.7 Hz, 1H, $H_{arom.}$), 6.57-6.43 (m, 2H, $H_{arom.}$), 4.62 (d, J = 12.5 Hz, 1H, OCH_2), 3.93 (d, J = 12.5 Hz, 1H, OCH_2) ppm. Monomer (major component): δ = 7.39 (d, J = 8.5 Hz, 2H, $H_{arom.}$), 7.10 (d, J = 8.5 Hz, 2H, $H_{arom.}$), 6.82 (dd, J = 7.8, 1.7 Hz, 1H, $H_{arom.}$), 6.29 (t, J = 7.8 Hz, 1H, $H_{arom.}$), 6.18 (dd, J = 7.8, 1.7 Hz, 1H, $H_{arom.}$), 5.13 (s, 2H, OCH_2) ppm. Signals not listed are overlapping and cannot be assigned. **MS** (negative ESI-MS, MeOH): m/z (%) = 1821.5790 (100, [M−Li^+], $C_{102}H_{96}O_{24}Li_3Ti_2^-$, calcd. 1821.5736). **IR** (KBr): $\tilde{\nu}$ (cm^{-1}) = 3358, 2959, 2296, 2115, 1900, 1676, 1593, 1560, 1515, 1443, 1381, 1292, 1252, 1214, 1152, 1061, 1003, 845, 795, 742, 678. **Elemental analysis** $C_{102}H_{96}O_{24}Li_4Ti_2 \cdot 4\ H_2O$: calcd. C = 64.43 %, H = 5.51 %; found C = 64.24 %, H = 5.43 %.

3. X-ray Crystallographic studies

X-ray crystallography of [Li$_3$(3^{2Bu}$_3$Ti)$_2$]$^-$ and [Li$_3$(3^{3Bu}$_3$Ti)$_2$]$^-$

Single-crystal X-ray data for [Li$_3$(**3**^{2Bu}$_3$Ti)$_2$]$^-$ and [Li$_3$(**3**^{3Bu}$_3$Ti)$_2$]$^-$ were measured using a Rigaku SuperNova dual-source Oxford diffractometer equipped with an Atlas detector using mirror-monochromated Cu-*K*α (λ = 1.54184 Å) radiation. The data collection and reduction were performed using the program *CrysAlisPro*[1] and Gaussian face index absorption correction method was applied.[1]

The structures were solved with direct methods (*SHELXS*)[2] or intrinsic phasing (SHELXT)[3] and refined by full-matrix least squares on F^2 using the *OLEX2* software,[4] which utilizes the *SHELXL*-2015 module.[2]

Crystal data for $[Li_3(\mathbf{2}^{\mathbf{All}}{}_3Ti)_2]^-$: CCDC-1919186, $C_{62}H_{56}Li_3NaO_{26}Ti_2$, M = 1356.67, orange block, 0.181 x 0.089 x 0.053 mm^3, monoclinic, space group *C2/c*, a = 16.3779(1) Å, b = 24.7920(3) Å, c = 16.0382(2) Å , *β* = 97.504(1)°, *V* = 6456.39(12) Å^3, Z = 4, D_{calc} = 1.396 g/cm^3, F000 = 2800, μ= 2.88 mm^{-1} , T = 120.0(1) K, θ_{max} = 76.7°, 16731 total reflections, 6446 with $I_o > 2\sigma(I_o)$, R_{int} = 0.020, 6631 data, 618 parameters, 684 restraints, GooF = 1.17, R = 0.131 and wR= 0.309 [$I_o > 2\sigma(I_o)$], R = 0.133 and wR= 0.308 (all reflections), 0.63 <dΔρ < -0.65 e/Å^3.

Crystal data for $[Li_3(\mathbf{3}^{\mathbf{2Bu}}{}_3Ti)_2]^-$: CCDC-1919187, $C_{137}H_{120}Li_8O_{55}Ti_4$, M = 2893.44, red block, 0.113 x 0.094 x 0.056 mm^3, tetragonal, space group *I*$4_1$22, a = 20.35612(19) Å, b = 20.35612(19) Å, c = 37.2292(4) Å, *β* = 90°, *V* = 15426.7(3) Å^3, Z = 4, D_{calc} = 1.246 g/cm^3, F000 = 5976, μ= 2.405 mm^{-1} , T = 120.0(1) K, θ_{max} = 66.745°, 68695 total reflections, 6414 with $I_o > 2\sigma(I_o)$, R_{int} = 0.0501, 6839 data, 525 parameters, 52 restraints, GooF = 1.091, R = 0.0522 and wR= 0.1478 [$I_o > 2\sigma(I_o)$], R = 0.0550 and wR= 0.1506 (all reflections), 0.796 <dΔρ < -0.417 e/Å^3.

Crystal data for $[Li_3(\mathbf{3}^{\mathbf{3Bu}}{}_3Ti)_2]^-$: CCDC-1919188, $C_{68}H_{58}Li_4O_{27}Ti_2$, M = 1430.70, orange block, 0.129 x 0.064 x 0.045 mm^3, monoclinic, space group *P*2_1/c, a = 13.4281(3) Å, b = 19.9504(3) Å, c = 25.6237(6) Å, *β* = 103.962(2)°, *V* = 6661.7(2) Å^3, Z = 4, D_{calc} = 1.427 g/cm^3, F000 = 2952, μ= 2.773 mm^{-1} , T = 120.01(10) K, θ_{max} = 66.749°, 25360 total reflections, 8619 with $I_o > 2\sigma(I_o)$, R_{int} = 0.0388, 11747 data, 961 parameters, 40 restraints, GooF = 1.096, R = 0.0641 and wR= 0.1682 [$I_o > 2\sigma(I_o)$], R = 0.0883 and wR= 0.1866 (all reflections), -0.872 <dΔρ < 1.096 e/Å^3.

X-ray crystallography of aromatic complexes

Exceptions and special features: Several badly disordered solvent molecules were found in all structures in the asymmetrical unit and could not be satisfactorily refined. The program SQUEEZE[8] was therefore used to remove mathematically the effect of the solvent. The quoted formula and derived parameters are not including the squeezed solvent molecules.

X-ray crystal structure analysis of $Li[Li_3(\mathbf{4}^{\mathbf{Bz}}{}_3Ti)_2]$:

For compound $Li[Li_3(\mathbf{4}^{\mathbf{Bz}}{}_3Ti)_2]$ data sets were collected with a Nonius Kappa CCD diffractometer. Programs used: data collection, COLLECT[5]; data reduction Denzo-SMN[6]; absorption correction, Denzo[7]; structure solution *SHELXT-2015*[3]; structure refinement *SHELXL-2015*[2]. *R*-values are given for observed reflections, and wR^2 values are given for all reflections.

A red needle-like specimen of $C_{84}H_{60}Li_3O_{24}Ti_2$, approximate dimensions 0.070 mm x 0.100 mm x 0.500 mm, was used for the X-ray crystallographic analysis. The X-ray intensity data were measured. The integration of the data using a monoclinic unit cell yielded a total of 57103 reflections to a maximum θ angle of 25.00° (0.84 Å resolution), of which 30206 were independent (average redundancy 1.890, completeness = 98.9%, R_{int} = 9.77%, R_{sig} = 11.48%) and 15761 (52.18%) were greater than $2\sigma(F^2)$. The final cell constants of a = 50.7797(6) Å, b = 32.5267(5) Å, c = 21.7023(4) Å, β = 104.7810(10)°, volume = 34659.4(9) Å^3, are based upon the refinement of the XYZ-centroids of reflections above 20 σ(I). Data were corrected for absorption effects using the multi-scan method (SADABS). The calculated

minimum and maximum transmission coefficients (based on crystal size) are 0.8830 and 0.9820. The structure was solved and refined using the Bruker SHELXTL Software Package, using the space group *C*2/*c*, with Z = 16 for the formula unit, $C_{84}H_{60}Li_3O_{24}Ti_2$. The final anisotropic full-matrix least-squares refinement on F^2 with 1891 variables converged at R1 = 11.17%, for the observed data and wR2 = 28.54% for all data. The goodness-of-fit was 1.046. The largest peak in the final difference electron density synthesis was 0.532 $e^-/Å^3$ and the largest hole was -0.406 $e^-/Å^3$ with an RMS deviation of 0.067 $e^-/Å^3$. On the basis of the final model, the calculated density was 1.203 g/cm^3 and F(000), 12944 e^-. CCDC number: 1950786.

X-ray crystallography of $[Li_3(4^{2Me}{}_3Ti)_2]^-$ and $[Li_3(4^{2,4,6Me}{}_3Ti)_2]^-$

Single crystal x-ray diffraction data were collected by using ω-scans on a Stoe Stadivari diffractometer with an Eulerian 4-circle geometry, equipped with a Cu-K_α micro focus source (GeniX 3D HF Cu) and a Pilatus 200K hybrid pixel detector (Dectris) at 100(2) K. Data collection and absorption correction were performed with the software package X-Area (STOE, X-Area 1.77, 2017), space group determination was performed with XPREP (1997). The structures were solved using a direct method with SHELXS (2013/1), structure refinement was carried out using SHELXL (2018/3) with a least squares procedure against F^2.

$Li[Li_3(4^{2,4,6Me}{}_3Ti)_2]$: CCDC-1917460, $C_{51}H_{48}Li_2O_{12}Ti$, M = 914.67, orange hexagonal block, 0.18 x 0.18 x 0.08 mm^3, trigonal, space group R-3, a = 28.361(4) Å, c = 11.297(2) Å, V = 7870(3) Å^3, Z = 6, D_{calc} = 1.158 g cm^{-3}, F(000) = 2868, m = 1.84 mm^{-1}, T = 100(2) K, q_{max} = 70.3°, 32946 total reflections, 3317 independent reflections, 2184 observed reflections [I > 2s(I)], 250 parameters, 72 restraints, R_{int} = 0.062, R[F^2 > 2s(F^2)] = 0.181, wR(F^2) = 0.474, S = 2.99, 1.43 < dDρ < -0.41 e/Å^3.

$Na[Li_3(4^{2Me}{}_3Ti)_2]$: CCDC-1917415, $C_{91}H_{76}Li_3NaO_{25}Ti_2$, 1709.12, orange block, 0.18 × 0.12 × 0.08 mm^3, monoclinic, space group $P2_1/n$, a = 15.375(3) Å, b = 21.115(4) Å, c = 25.156(5) Å, b = 91.40(3)°, V = 8164(3) Å^3, Z = 4, D_{calc} = 1.391 g cm^{-3}, F(000) = 3544, m = 2.39 mm^{-1}, T = 100(2) K, q_{max} = 71.0°, 42865 total reflections, 14327 independent reflections, 8177 observed reflections [I > 2s(I)], 785 parameter, 39 restraints, R_{int} = 0.053, R[F^2 > 2s(F^2)] = 0.117, wR(F^2) = 0.287, S = 1.40, 1.63 < dDρ < -0.56 e/Å^3.

4. Computational considerations

The structures of the helicates were optimized at the Hartree Fock level of ab initio theory employing the 6-31G* set of contracted gaussian functions. Based on the optimized structures dispersion interaction between the side chains of the helicates was then approximated using the London formula

$$E_{disp} = -\frac{3}{2}\cdot\frac{U_A\cdot U_B}{U_A+U_B}\sum_a\sum_b \alpha_a\cdot\alpha_b\cdot r_{ab}^{-6}$$

were U_A and U_B are the ionization potentials of the interacting substituents approximated by the Koopmans ionization potentials of the corresponding helicates. Since we study the interaction between side chains in one and the same molecule in our case we have $U_A = U_B$. Values between 5.00 and 5.08 eV were found for the compounds under consideration.

α_a and α_b are the polarizabilities of the atoms of substituents *a* and *b*, respectively, and r_{ab} is the corresponding interatomic distance. Only hydrogen and carbon atoms were include in these calculations. We used α_H = 0.43 and α_C = 1.51Å^3 which were derived from the increments in Table 2 of ref.[9] scaled by the correlation given in the caption to Table 3 of ref.[9].

In a first series of calculation we included only the atoms of the aliphatic substituents of the ester groups resulting in $E_{disp,a}$ while in a second run we included both the atoms of the aliphatic and aromatic residues which gives $E_{disp,b}$. From $E_{disp,b}$ - $E_{disp,a}$ = $E_{disp,c}$ we then obtain an approximation for the interaction between the aromatic and aliphatic substituents. The results are given in Table 1.

Table 1. All values in kcal/mol

Substituent	$E_{disp,a}$	$E_{disp,b}$	$E_{disp,c}$
$[Li_3(\mathbf{1}^{Me}{}_3Ti)_2]^-$	-0.10	-8.00	-7.90
$[Li_3(\mathbf{1}^{Et}{}_3Ti)_2]^-$	-0.49	-12.40	-11.91
$[Li_3(\mathbf{1}^{Pr}{}_3Ti)_2]^-$	-0.30	-11.56	-11.26
$[Li_3(\mathbf{1}^{Bu}{}_3Ti)_2]^-$	-0.36	-12.17	-11.81
$[Li_3(\mathbf{1}^{Pent}{}_3Ti)_2]^-$	-0.40	-12.38	-11.98
$[Li_3(\mathbf{1}^{Hex}{}_3Ti)_2]^-$	-0.45	-12.83	-12.38
$[Li_3(\mathbf{1}^{Hept}{}_3Ti)_2]^-$	-0.45	-12.55	-12.10
$[Li_3(\mathbf{1}^{Oct}{}_3Ti)_2]^-$	-0.46	-12.57	-12.11
$[Li_3(\mathbf{1}^{Non}{}_3Ti)_2]^-$	-0.47	-12.60	-12.13
$[Li_3(\mathbf{1}^{Dec}{}_3Ti)_2]^-$	-0.47	-12.61	-12.14
$[Li_3(\mathbf{1}^{Undec}{}_3Ti)_2]^-$	-0.48	-12.79	-12.31
$[Li_3(\mathbf{2}^{Z2Hex}{}_3Ti)_2]^-$	-0.38	-15.20	-14.82
$[Li_3(\mathbf{2}^{E2Hex}{}_3Ti)_2]^-$	-0.34	-11.64	-11.30
$[Li_3(\mathbf{3}^{2Hex}{}_3Ti)_2]^-$	-0.36	-10.57	-10.22

The results of our analysis are summarized as follows: The dispersion interaction between the unbranched hydrocarbon chains is weak. Branched and cyclic substituents give somewhat higher values between -1.0 and -2.1 and between -0.9 and -4.8 kcal/mol, respectively. Much stronger are the interactions between the aromatic rings and the aliphatic side chains which cover the range between -7.9 for methyl up to -14.8 kcal/mol for the *Z*-alken.

5. References

[1] Rigaku Oxford Diffraction, 2018, *CrysAlisPro* Software system, version 38.46, Rigaku Corporation, Oxford, UK

[2] G. M. Sheldrick, *Acta Crystallogr. Sect. C,* **2015**, *71*, 3–8

[3] G. M. Sheldrick, *Acta Crystallogr. Sect. A*, **2015**, *71*, 3–8
[4] O. V Dolomanov, L. J. Bourhis, R. J. Gildea, J. A. K. Howard, H. Puschmann, *J. Appl. Crystallogr.*, **2009**, *42*, 339–341.
[5] R. W. W. Hooft, Bruker AXS, **2008**, Delft, The Netherlands.
[6] Z. Otwinowski, W. Minor, *Methods Enzymol.* **1997**, *276*, 307-326.
[7] Z. Otwinowski, D. Borek, W. Majewski, W. Minor, *Acta Crystallogr.* **2003**, *A59*, 228-234.
[8] A. L. Spek, *Acta Cryst. Sect C.* **2015**, *71*, 9-18.
[9] G. Raabe, E. Zobel, R. Kock, J. P. Souren, *Z. Naturforsch. A* **1997**, *52a*, 665-674.
